# Evaluation of Prompts to Simplify Cardiovascular Disease Information Generated Using a Large Language Model: Cross-Sectional Study

**DOI:** 10.2196/55388

**Published:** 2024-04-22

**Authors:** Vishala Mishra, Ashish Sarraju, Neil M Kalwani, Joseph P Dexter

**Affiliations:** 1 Department of Biostatistics and Bioinformatics Duke University School of Medicine Durham, NC United States; 2 Department of Cardiovascular Medicine Cleveland Clinic Cleveland, OH United States; 3 Veterans Affairs Palo Alto Health Care System Palo Alto, CA United States; 4 Division of Cardiovascular Medicine and the Cardiovascular Institute, Department of Medicine Stanford University School of Medicine Stanford, CA United States; 5 Data Science Initiative Harvard University Allston, MA United States; 6 Department of Human Evolutionary Biology Harvard University Cambridge, MA United States; 7 Institute of Collaborative Innovation University of Macau Taipa Macao

**Keywords:** artificial intelligence, ChatGPT, GPT, digital health, large language model, NLP, language model, language models, prompt engineering, health communication, generative, health literacy, natural language processing, patient-physician communication, health communication, prevention, cardiology, cardiovascular, heart, education, educational, human-in-the-loop, machine learning

## Abstract

In this cross-sectional study, we evaluated the completeness, readability, and syntactic complexity of cardiovascular disease prevention information produced by GPT-4 in response to 4 kinds of prompts.

## Introduction

Many web-based patient educational materials about cardiovascular disease (CVD) are inaccessible for the general public [1]. Artificial intelligence (AI) chatbots powered by large language models (LLMs) are a potential source of public-facing CVD information [2-4]. Generative language models present risks related to information quality but also opportunities for producing accessible information about CVD at scale, which could advance the American Heart Association’s 2020 impact goals related to health literacy [5]. Recent studies have used LLMs to simplify medical information in different contexts [3,6-8], but quantitative comparison of prompt engineering strategies is needed to assess and optimize performance and to ensure that the rapid deployment of clinical AI tools proceeds in an equitable manner [9]. In this cross-sectional study, we evaluated the completeness, readability, and syntactic complexity of CVD prevention information produced by GPT-4 in response to 4 kinds of prompts.

## Methods

A set of 25 questions about fundamental CVD prevention topics was drawn from a previous study, which found that the GPT 3.5 version of ChatGPT provided generally appropriate responses [2]. We devised 3 prompt strategies for generating simplified ChatGPT responses to these questions, including a zero-shot prompt to use plain and easy-to-understand language, a one-shot prompt with a sample simplified passage on an unrelated subject, and a combined prompt to use simplified language and cover specific key points (which we termed “rubric prompting”; Multimedia Appendix 1). Responses to these three prompts were compared to baseline responses for which the prompt contained only the question about CVD. The full set of responses is provided in Multimedia Appendix 2.

For each question and prompt type, 3 independent responses were generated between April and June 2023, using the GPT-4 version of ChatGPT with default parameters, which was available from OpenAI through a ChatGPT Plus subscription. Two authors, who are preventive cardiologists (AS and NWK), scored the responses as “complete,” “incomplete,” or “inconsistent” according to a custom rubric (Multimedia Appendix 3); disagreements were resolved by consensus. For all generated responses, we calculated 5 readability scores, using Readability Studio Professional (version 2019.3; Oleander Software), and 2 measures of syntactic complexity, using the L2 Syntactic Complexity Analyzer (version 3.3.3), as described previously [10].

Differences from baseline completeness were assessed using the Fisher exact test, and 2-sample readability and syntactic complexity comparisons were done using the Wilcoxon rank-sum test. Statistical significance was set as *P*<.05.

## Results

Baseline responses to 80% (20/25) of the questions were scored as “complete” (Table 1). Completeness was significantly lower for both the zero-shot (8/25, 32%; *P*=.001) and one-shot (8/25, 32%; *P*=.001) simplification prompts but significantly higher for the rubric prompts (25/25, 100%; *P*=.001). All 3 prompts significantly improved readability according to every metric and lowered 1 measure of syntactic complexity (Table 2).

**Table 1:** Evaluation of the completeness of cardiovascular disease information generated using 4 large language model prompt strategies.

Question	Consensus grade for each prompt^a^			
	Baseline	Plain language (zero-shot prompt)	Plain language (one-shot prompt)	Plain language (rubric prompt)
How can I prevent heart disease?	Complete	Complete	Complete	Complete
What is the best diet for the heart?	Complete	Complete	Complete	Complete
What is the best diet for high blood pressure and high cholesterol?	Complete	Complete	Complete	Complete
How much should I exercise to stay healthy?	Complete	Inconsistent	Incomplete	Complete
Should I do cardio or lift weights to prevent heart disease?	Complete	Inconsistent	Inconsistent	Complete
How can I lose weight?	Complete	Inconsistent	Inconsistent	Complete
How can I decrease LDL^b^?	Inconsistent	Incomplete	Incomplete	Complete
How can I decrease triglycerides?	Complete	Complete	Complete	Complete
What is lipoprotein(a)?	Complete	Incomplete	Incomplete	Complete
How can I quit smoking?	Complete	Complete	Inconsistent	Complete
What are the side effects of statins?	Complete	Inconsistent	Complete	Complete
I have muscle pain with a statin. What should I do?	Inconsistent	Inconsistent	Complete	Complete
My cholesterol is still high and I’m already on a statin. What should I do?	Inconsistent	Incomplete	Incomplete	Complete
What medications can reduce cholesterol other than statins?	Complete	Complete	Inconsistent	Complete
What is ezetimibe?	Complete	Inconsistent	Incomplete	Complete
What are Repatha and Praluent?	Complete	Incomplete	Incomplete	Complete
What is inclisiran?	Complete	Incomplete	Incomplete	Complete
What are the side effects of Repatha and Praluent?	Complete	Complete	Inconsistent	Complete
Should I take aspirin to prevent heart disease?	Complete	Complete	Complete	Complete
My cholesterol panel shows triglycerides 400 mg/dL. How should I interpret this?	Complete	Inconsistent	Complete	Complete
My LDL is 200 mg/dL. How should I interpret this?	Inconsistent	Incomplete	Incomplete	Complete
What does a coronary calcium score of 0 mean?	Complete	Incomplete	Incomplete	Complete
What does a coronary calcium score of 100 mean?	Inconsistent	Inconsistent	Incomplete	Complete
What does a coronary calcium score of 400 mean?	Complete	Incomplete	Incomplete	Complete
What genetic mutations can cause high cholesterol?	Complete	Inconsistent	Incomplete	Complete

^a^For every prompt strategy, we generated 3 responses to each of the 25 questions about cardiovascular disease prevention. “Complete” indicates that all 3 responses received a full score according to our coverage rubric, “Incomplete” indicates that all 3 responses received less than a full score, and “Inconsistent” indicates that some responses were “Complete” and others were “Incomplete.” Grades shown were determined by consensus between 2 reviewers.

^b^LDL: low-density lipoprotein.

**Table 2:** Comparison of the readability and syntactic complexity of cardiovascular disease information generated using 4 large language model prompt strategies.^a^

			Prompts												
			Baseline, median (IQR)		Plain language (zero-shot prompt)				Plain language (one-shot prompt)				Plain language (rubric prompt)		
					Value, median (IQR)		Difference from baseline^b^, median (IQR; *P* value)		Value, median (IQR)		Difference from baseline^c^, median (IQR; *P* value)		Value, median (IQR)		Difference from baseline^d^, median (IQR; *P* value)
**Readability formulas**															
	FKGL^e^	13.4 (12.3 to 15.4)		9.7 (7.6 to 11.1)		−4.2 (−5.7 to −3.1; <.001)		3.8 (2.9 to 5.3)		−9.4 (−11.1 to −8.3; <.001)		8.0 (7.3 to 9.5)		−5.3 (−6.6 to −4.0; <.001)	
	SMOG^f^	14.8 (13.7 to 16.5)		12.1 (10.2 to 13)		−3.6 (−4.5 to −2.4; <.001)		7.9 (7.2 to 9.2)		−7.1 (−8.2 to −5.7; <.001)		10.9 (10.4 to 11.9)		−4.1 (−5.4 to −3.0; <.001)	
	GFI^g^	14.0 (12.1 to 17)		11.3 (8.0 to 13)		−4.0 (−5.6 to −2.7; <.001)		6.3 (5.4 to 7.6)		−7.5 (−10.3 to −6.0; <.001)		10.2 (8.9 to 11.3)		−3.9 (−6.3 to −2.8; <.001)	
	FORCAST^h^	11.5 (11.2 to 11.9)		10.2 (9.8 to 10.7)		−1.3 (−1.8 to −0.9; <.001)		8.8 (8.2 to 9.4)		−2.7 (−3.4 to −2.3; <.001)		9.7 (9.3 to 10.2)		−1.9 (−2.3 to −1.4; <.001)	
	CLI^i^	13.8 (13.2 to 15.1)		10.4 (9.0 to 11.8)		−3.7 (−4.7 to −2.4; <.001)		6.2 (5.1 to 7.3)		−7.9 (−9.0 to −6.5; <.001)		9.4 (9.0 to 10.4)		−4.5 (−5.4 to −3.5; <.001)	
**Syntactic complexity** ^j^															
	MLC^k^	15.0 (12.7 to 16.6)		12.3 (10.5 to 15.5)		−1.8 (−4.4 to 0.9; .01)		8.7 (7.8 to 10.7)		−5.7 (−7.6 to −3.4; <.001)		9.6 (8.9 to 10.3)		−4.2 (−6.9 to −3.1; <.001)	
	DC/T^l^	0.3 (0.2 to 0.5)		0.3 (0.2 to 0.5)		0 (−0.2 to 0.1; .36)		0.2 (0.1 to 0.3)		−0.2 (−0.3 to −0.1; <.001)		0.6 (0.4 to 0.7)		0.2 (0.1 to 0.4; >.99)	

^a^For every prompt strategy, we generated 3 responses to each of the 25 questions about cardiovascular disease prevention. Lower scores indicate higher readability.

^b^Difference between responses to the baseline prompts and prompts for plain language. *P* values are from a 1-tailed Wilcoxon signed rank test.

^c^Difference between responses to the baseline prompts and prompts for plain language with an example. *P* values are from a 1-tailed Wilcoxon signed rank test.

^d^Difference between responses to the baseline prompts and prompts for plain language with coverage. *P* values are from a 1-tailed Wilcoxon signed rank test.

^e^FKGL: Flesch-Kincaid Grade Level.

^f^SMOG: Simple Measure of Gobbledygook.

^g^GFI: Gunning Fog Index.

^h^FORCAST: Ford, Caylor, Sticht formula.

^i^CLI: Coleman-Liau Index.

^j^MLC is a measure of elaboration at the clause level (ie, number of words per clause), and DC/T is a measure of subordination.

^k^MLC: mean length of clause.

^l^DC/T: dependent clauses/T-unit.

## Discussion

We found that zero- and one-shot prompting of GPT-4 to produce simplified information about CVD generated more readable but less comprehensive responses. This loss of information, however, could be averted by combining a zero-shot simplification prompt with a short reminder to include critical information (rubric prompting). Our findings highlight the importance of optimizing prompts and incorporating expert clinical judgment when considering the use of LLMs to produce patient education materials, including AI-drafted replies to patient messages [3,6,7]. Accordingly, prospective guidelines for the use of AI in medicine should address best practices for prompt engineering, standardized evaluation of model outputs, and outreach to clinicians and the public to cultivate relevant skills [11]. Such guidelines will provide important parameters for clinician-in-the-loop information simplification systems [6,12,13], which have already been deployed to improve the accessibility of surgical consent forms [14].

The limitations of this study include the evaluation of a single model at a specific point in time and the absence of reading comprehension data from patients. Since the prompt strategies developed herein are not model specific, it should be straightforward to extend these strategies to other LLMs. Future research should further evaluate trade-offs between prompt engineering and fine-tuning of LLMs for medical applications using multiple models. It would also be useful to integrate ongoing user testing with structured health literacy assessment of generated responses to identify types of simplification that are especially important for improving patient understanding.

## Appendix

Multimedia Appendix 1 Example prompt types.

Multimedia Appendix 2 Full ChatGPT responses.

Multimedia Appendix 3 Custom scoring rubric.

## Abbreviations

AI: artificial intelligence
CVD: cardiovascular disease
LLM: large language model
