# Supplementary material for: Evaluation of Prompts to Simplify Cardiovascular Disease Information Generated Using a Large Language Model: Cross-Sectional Study
Source: J Med Internet Res. 2024 Apr 22;26:e55388. doi: 10.2196/55388 (PMC11074888; doi:10.2196/55388)
Supplement: Multimedia Appendix 1 [file jmir_v26i1e55388_app1.docx]

**Multimedia Appendix 1: Example Prompt Types**

| **Prompt Type** | **Example** |
| --- | --- |
| Baseline | How can I prevent heart disease? |
| Plain Language (Zero-Shot) | How can I prevent heart disease?  Answer this question using plain and easy-to-understand language in your response. |
| Plain Language (One-Shot) | How can I prevent heart disease? Answer this question using plain and easy-to-understand language in your response. This text about COVID-19 is a good example of the writing style (not content) you should use.  What is COVID-19?  • It is a new illness spreading around the world.  • It’s nickname is coronavirus.  How do you get it?  • Someone with COVID-19 gives you their germs.  • When they cough or sneeze, their germs get in the air, on you, and on things.  • Germs get into your body through your mouth, nose, and your eyes. |
| Plain Language (Rubric) | How can I prevent heart disease?  Answer this question using plain and easy-to-understand language in your response. Your response should mention diet, exercise, and risk factor control and should also recommend talking with a healthcare professional. |
