# Supplementary material for: Evaluation of Prompts to Simplify Cardiovascular Disease Information Generated Using a Large Language Model: Cross-Sectional Study
Source: J Med Internet Res. 2024 Apr 22;26:e55388. doi: 10.2196/55388 (PMC11074888; doi:10.2196/55388)
Supplement: Multimedia Appendix 2 [file jmir_v26i1e55388_app2.docx]

**Multimedia Appendix 2: Full ChatGPT Responses**

**Baseline**

**How can I prevent heart disease?**

***

I'm not a doctor, but I can provide you with some general suggestions for reducing your risk of heart disease. It's important to consult a healthcare professional for personalized advice. That being said, here are some lifestyle changes that may help:

1. Eat a healthy diet: Aim for a balanced diet that includes whole grains, fruits, vegetables, lean proteins, and healthy fats. Limit saturated and trans fats, added sugars, and excessive salt intake.
2. Exercise regularly: Engage in at least 150 minutes of moderate-intensity aerobic exercise or 75 minutes of vigorous-intensity exercise per week, spread across several days. Include muscle-strengthening activities at least twice a week.
3. Maintain a healthy weight: Obesity can increase your risk of heart disease. Aim for a body mass index (BMI) within the normal range (18.5-24.9).
4. Quit smoking: If you smoke, quitting is one of the most significant steps you can take to reduce your risk of heart disease.
5. Limit alcohol intake: Consume alcohol in moderation, which generally means up to one drink per day for women and up to two drinks per day for men.
6. Manage stress: Find healthy ways to cope with stress, such as through relaxation techniques, meditation, or physical activity.
7. Monitor and control blood pressure: Regularly check your blood pressure and, if it's high, work with your healthcare provider to manage it through lifestyle changes or medication, if necessary.
8. Manage diabetes: If you have diabetes, work with your healthcare provider to maintain healthy blood sugar levels through diet, exercise, and medication, if necessary.
9. Control cholesterol: Aim to maintain healthy cholesterol levels by adopting a heart-healthy diet and exercising regularly. If needed, your doctor may prescribe medication to help manage your cholesterol.
10. Get regular check-ups: Visit your healthcare provider regularly for check-ups and screenings to help detect potential issues early on.

Remember to consult with a healthcare professional for personalized advice and to discuss any concerns you may have about heart disease prevention.

***

I am not a doctor, but I can offer some general advice on how to reduce the risk of heart disease based on common recommendations. To improve your heart health, consider the following lifestyle changes:

1. Eat a healthy diet: Opt for a diet rich in fruits, vegetables, whole grains, lean protein, and healthy fats. Limit your intake of processed foods, saturated fats, trans fats, added sugars, and sodium. Aim to consume more fiber and include omega-3 fatty acids in your diet.
2. Exercise regularly: Engage in at least 150 minutes of moderate-intensity aerobic activity or 75 minutes of vigorous-intensity aerobic activity per week. Incorporate strength training exercises into your routine at least twice a week.
3. Maintain a healthy weight: Excess weight can increase your risk of heart disease. Aim to maintain a healthy weight by balancing your calorie intake with your physical activity level.
4. Don't smoke: Smoking is a significant risk factor for heart disease. If you're a smoker, quitting is one of the best things you can do for your heart health.
5. Limit alcohol consumption: Moderate alcohol intake is generally considered safe for most people, but excessive drinking can lead to various health problems, including heart disease. Stick to the recommended guidelines, which typically advise no more than one drink per day for women and two drinks per day for men.
6. Manage stress: Chronic stress can contribute to heart disease. Find healthy ways to cope with stress, such as exercise, meditation, or seeking support from friends, family, or a mental health professional.
7. Monitor your blood pressure and cholesterol levels: High blood pressure and high cholesterol can increase your risk of heart disease. Get regular check-ups and work with your healthcare provider to manage these risk factors.
8. Manage diabetes: If you have diabetes, it's essential to keep your blood sugar levels under control to reduce the risk of heart disease.
9. Get enough sleep: Aim for 7-9 hours of quality sleep per night. Poor sleep can contribute to heart disease risk factors such as obesity, high blood pressure, and diabetes.

Always consult with your healthcare provider before making significant lifestyle changes or if you have concerns about your heart health. They can offer personalized advice based on your specific medical history and needs.

***

I am not a doctor, but I can provide you with some general information on how to prevent heart disease. It's important to consult with a healthcare professional for personalized advice. Here are some lifestyle changes that may help reduce the risk of heart disease:

1. Maintain a healthy weight: Obesity can increase your risk of heart disease, so it's important to maintain a healthy weight. A balanced diet and regular exercise can help you achieve this goal.
2. Eat a heart-healthy diet: Consume a diet rich in fruits, vegetables, whole grains, lean proteins, and healthy fats. Limit saturated and trans fats, added sugars, and salt. Include foods high in omega-3 fatty acids, like fish, nuts, and seeds, in your diet.
3. Exercise regularly: Aim for at least 150 minutes of moderate-intensity aerobic exercise or 75 minutes of vigorous-intensity aerobic exercise per week. Also, include strength training exercises at least twice a week.
4. Don't smoke: Smoking increases the risk of heart disease. If you smoke, quitting is one of the best things you can do for your heart health.
5. Limit alcohol intake: If you choose to drink alcohol, do so in moderation. This generally means up to one drink per day for women and up to two drinks per day for men.
6. Manage stress: Chronic stress can contribute to heart disease. Find healthy ways to cope with stress, such as meditation, exercise, or talking to a professional.
7. Maintain good dental hygiene: There is evidence linking poor dental health to heart disease. Brush and floss regularly, and visit your dentist for regular checkups.
8. Control blood pressure and cholesterol: High blood pressure and high cholesterol can increase your risk of heart disease. Keep these under control through a healthy diet, exercise, and medication, if necessary.
9. Manage diabetes: If you have diabetes, keeping your blood sugar levels in check can help prevent heart disease.
10. Get regular checkups: Regular checkups with your healthcare provider can help you stay on top of your heart health and address any potential issues early.

Remember to consult with a healthcare professional for personalized advice and guidance.

***

**What is the best diet for the heart?**

***

I am not a doctor, but I can provide some general information on heart-healthy diets. It is essential to consult with a healthcare professional before making any changes to your diet or lifestyle.

A heart-healthy diet typically emphasizes the following components:

1. Fruits and vegetables: Aim for a variety of colors and types, as they are rich in vitamins, minerals, and antioxidants.
2. Whole grains: Opt for whole grains like brown rice, quinoa, whole wheat, and oats, as they provide fiber and help control blood sugar levels.
3. Lean proteins: Choose lean protein sources like fish, poultry, beans, legumes, and low-fat dairy products. Fish, particularly fatty fish like salmon, mackerel, and sardines, contain heart-healthy omega-3 fatty acids.
4. Healthy fats: Include healthy fats from sources like avocados, nuts, seeds, and olive oil. These fats can help lower "bad" LDL cholesterol and raise "good" HDL cholesterol.
5. Limited saturated and trans fats: Reduce intake of saturated fats found in fatty meats, full-fat dairy products, and baked goods. Avoid trans fats, which are typically found in processed foods and partially hydrogenated oils.
6. Low sodium: Limit salt intake to help control blood pressure. Aim for less than 2,300 mg of sodium per day or as recommended by your healthcare professional.
7. Moderate alcohol consumption: If you choose to drink alcohol, do so in moderation. This means up to one drink per day for women and up to two drinks per day for men.

Some popular heart-healthy diets that incorporate these principles include the Mediterranean diet, the DASH diet (Dietary Approaches to Stop Hypertension), and the AHA (American Heart Association) diet.

Remember, it is essential to consult with a healthcare professional before making any changes to your diet or lifestyle, as individual needs may vary.

***

I am not a doctor, but I can provide some general information about heart-healthy diets. The best diet for heart health is typically one that is rich in fruits, vegetables, whole grains, lean proteins, and healthy fats while low in saturated and trans fats, sodium, and added sugars. A few well-known heart-healthy diets include:

1. Mediterranean diet: This diet emphasizes fruits, vegetables, whole grains, legumes, nuts, seeds, fish, and olive oil. It encourages moderate consumption of dairy, poultry, and eggs, and limited intake of red meat and sweets.
2. DASH (Dietary Approaches to Stop Hypertension) diet: Designed to lower high blood pressure, the DASH diet promotes consumption of fruits, vegetables, whole grains, lean proteins, and low-fat dairy while limiting sodium, sweets, and red meat.
3. Plant-based diets (e.g., vegetarian, vegan): These diets focus on consuming plant-based foods like fruits, vegetables, whole grains, legumes, nuts, and seeds, and they can also be beneficial for heart health when well-planned and balanced.

Remember that individual needs and preferences can vary, so it's essential to consult with a healthcare professional or registered dietitian before making significant changes to your diet. They can help tailor a heart-healthy diet that works best for you.

***

I am not a doctor, but I can provide you with some general information on heart-healthy diets. It's important to consult with a healthcare professional before making significant changes to your diet, as individual needs can vary.

In general, a heart-healthy diet emphasizes:

1. Fruits and vegetables: Aim for a variety of colorful fruits and vegetables, as they are rich in vitamins, minerals, and antioxidants.
2. Whole grains: Choose whole grains like whole wheat, brown rice, quinoa, and oats, as they can help to lower bad cholesterol and provide essential nutrients.
3. Lean proteins: Opt for lean protein sources such as fish, poultry, beans, and legumes. Fish like salmon and mackerel are high in omega-3 fatty acids, which have been shown to support heart health.
4. Healthy fats: Focus on healthy fats found in nuts, seeds, avocados, and olive oil, which can help to manage cholesterol levels and provide essential nutrients.
5. Low-fat dairy: Choose low-fat or fat-free dairy products to limit saturated fat intake.
6. Limit processed and red meats: Processed and red meats can be high in saturated fats and contribute to higher cholesterol levels.
7. Limit sodium intake: Excess sodium can contribute to high blood pressure, so it's important to monitor your intake and opt for lower-sodium alternatives when possible.
8. Limit added sugars and refined carbohydrates: These can contribute to weight gain, higher cholesterol levels, and increased risk of heart disease.

Some popular heart-healthy diet plans include the Mediterranean Diet, DASH (Dietary Approaches to Stop Hypertension) Diet, and the American Heart Association's Diet and Lifestyle Recommendations. Remember to consult with a healthcare professional to determine the best diet plan for your individual needs and circumstances.

***

**What is the best diet for high blood pressure and high cholesterol?**

***

I am not a doctor, but I can provide some general advice for managing high blood pressure and high cholesterol. It's always best to consult a healthcare professional for personalized advice.

A heart-healthy diet is typically recommended for individuals with high blood pressure and high cholesterol. The DASH (Dietary Approaches to Stop Hypertension) diet and the Mediterranean diet are two examples that have been shown to help manage these conditions.

Here are some general guidelines to consider:

1. Eat more fruits and vegetables: Aim for at least 5 servings per day, as they are rich in fiber, vitamins, minerals, and antioxidants.
2. Choose whole grains: Opt for whole wheat bread, brown rice, quinoa, and other whole grains, as they contain more fiber and nutrients compared to refined grains.
3. Include healthy fats: Consume monounsaturated and polyunsaturated fats found in olive oil, avocados, nuts, and fatty fish like salmon or mackerel, which can help lower cholesterol levels.
4. Limit saturated and trans fats: Reduce intake of fatty meats, full-fat dairy products, and processed foods, as these can raise cholesterol levels.
5. Consume lean proteins: Choose lean meats like poultry, fish, and plant-based proteins like legumes, beans, tofu, and tempeh.
6. Reduce sodium intake: Aim for less than 2,300 mg of sodium per day (or lower, depending on your doctor's advice). This can help lower blood pressure.
7. Limit added sugars: Avoid sugary drinks and processed foods high in added sugars.
8. Moderate alcohol consumption: If you drink alcohol, do so in moderation – up to one drink per day for women and up to two drinks per day for men.
9. Maintain a healthy weight: Achieving and maintaining a healthy weight can help lower blood pressure and cholesterol levels.
10. Stay physically active: Regular exercise can help lower blood pressure, improve cholesterol levels, and maintain a healthy weight.

Remember to consult your healthcare professional before making any significant changes to your diet or lifestyle, as they can provide tailored advice based on your specific needs and conditions.

***

I am not a doctor, but I can provide some general information on diets that may be helpful for managing high blood pressure and high cholesterol. It's important to consult with a healthcare professional before making any significant changes to your diet.

1. DASH Diet (Dietary Approaches to Stop Hypertension): The DASH diet is designed specifically to help lower blood pressure. It emphasizes fruits, vegetables, whole grains, low-fat dairy products, lean protein sources, nuts, seeds, and legumes. It is low in sodium, saturated fat, and added sugars.
2. Mediterranean Diet: This diet is based on the traditional eating patterns of people living in the Mediterranean region. It emphasizes fruits, vegetables, whole grains, legumes, nuts, seeds, and healthy fats, such as olive oil. Fish and seafood are the primary sources of animal protein, while poultry, eggs, and dairy are consumed in moderation. Red meat and added sugars are limited.
3. Plant-Based Diet: A plant-based diet focuses on consuming whole, minimally processed plant foods, such as fruits, vegetables, whole grains, legumes, nuts, and seeds. This diet tends to be high in fiber and low in saturated fat, which may be beneficial for managing high blood pressure and high cholesterol.

General tips for managing high blood pressure and high cholesterol:

- Limit sodium intake: Aim to consume no more than 2,300 mg of sodium per day, or even less if advised by your healthcare professional.
- Reduce saturated and trans fats: Limit your intake of foods high in saturated fat, such as fatty cuts of meat, full-fat dairy products, and processed foods. Avoid trans fats, which are found in some packaged and fried foods.
- Increase dietary fiber: Consume plenty of fruits, vegetables, whole grains, legumes, nuts, and seeds to increase your fiber intake.
- Maintain a healthy weight: Losing excess weight may help lower both blood pressure and cholesterol levels.
- Engage in regular physical activity: Aim for at least 150 minutes of moderate-intensity aerobic exercise or 75 minutes of vigorous-intensity aerobic exercise per week.
- Limit alcohol consumption: Moderate alcohol intake to no more than one drink per day for women and two drinks per day for men.

Remember to consult a healthcare professional before making any significant changes to your diet or lifestyle, as individual needs and circumstances can vary.

***

I am not a doctor, but I can provide some general information on diets that may help with high blood pressure and high cholesterol. It's essential to consult with a healthcare professional before making significant changes to your diet.

1. DASH Diet (Dietary Approaches to Stop Hypertension): This diet focuses on consuming fruits, vegetables, whole grains, low-fat dairy, lean proteins, and nuts. It emphasizes reducing sodium intake, added sugars, and unhealthy fats.
2. Mediterranean Diet: This diet is rich in fruits, vegetables, whole grains, legumes, nuts, and healthy fats like olive oil. It also includes moderate amounts of fish, poultry, dairy, and red wine. The Mediterranean diet emphasizes limiting red and processed meats, added sugars, and unhealthy fats.
3. Plant-Based Diet: A plant-based diet is primarily composed of fruits, vegetables, whole grains, legumes, nuts, and seeds, while minimizing animal products. This diet can also be helpful for managing high blood pressure and cholesterol.

General recommendations:

- Limit sodium intake to under 2,300 mg per day (or even lower, depending on your healthcare professional's advice).
- Consume a variety of fruits and vegetables.
- Choose whole grains over refined grains.
- Opt for lean proteins, such as fish, poultry, and plant-based sources like legumes and tofu.
- Avoid trans fats and limit saturated fats.
- Include healthy fats, such as those found in avocados, nuts, and olive oil.
- Limit alcohol consumption and avoid tobacco use.
- Maintain a healthy weight and engage in regular physical activity.

Always consult with a healthcare professional to determine the best dietary approach for your specific needs and conditions.

***

**How much should I exercise to stay healthy?**

***

I'm not a doctor or a certified fitness professional, but I can provide some general guidelines to help you understand how much exercise you might need to stay healthy. The American Heart Association and the World Health Organization recommend the following for adults:

1. At least 150 minutes of moderate-intensity aerobic activity (e.g., brisk walking, swimming, or cycling) or 75 minutes of vigorous-intensity aerobic activity (e.g., running, HIIT, or fast cycling) per week. This can be broken down into sessions of at least 10 minutes each.
2. Muscle-strengthening activities that involve all major muscle groups should be done on 2 or more days per week. These activities can include resistance training, bodyweight exercises, or yoga.
3. Flexibility exercises, such as stretching or Pilates, can be done on a regular basis to improve range of motion and prevent injury.
4. Balance exercises, such as tai chi or standing on one leg, can be done regularly, especially for older adults or those at risk for falls.

It's essential to remember that these are general guidelines, and individual needs may vary. Please consult with a healthcare professional or a certified fitness expert before starting a new exercise program, especially if you have pre-existing medical conditions or concerns.

***

While I am not a healthcare professional, I can provide you with some general guidelines that are commonly recommended for maintaining a healthy lifestyle through exercise.

The American Heart Association (AHA) and the Centers for Disease Control and Prevention (CDC) typically recommend the following for adults:

1. At least 150 minutes of moderate-intensity aerobic exercise or 75 minutes of vigorous-intensity aerobic exercise per week, spread out across three or more days per week. You can also combine moderate and vigorous activities to meet this goal.
   - Moderate-intensity activities: brisk walking, swimming, or biking on flat terrain.
   - Vigorous-intensity activities: running, fast cycling, or swimming laps.
2. Muscle-strengthening activities on two or more days per week that involve all major muscle groups (legs, hips, back, chest, shoulders, and arms).
   - Examples: weightlifting, resistance bands, bodyweight exercises (push-ups, squats, lunges), or yoga.
3. Flexibility exercises, like stretching or yoga, should be incorporated into your routine to maintain or improve your range of motion.

These are general recommendations, and individual needs may vary depending on factors such as age, sex, weight, and existing health conditions. It is always best to consult with a healthcare professional, like a doctor or a physical therapist, to develop an exercise plan tailored to your specific needs and goals.

***

I am not a doctor, but I can provide general recommendations on exercise for maintaining good health. The American Heart Association and the Centers for Disease Control and Prevention (CDC) suggest that adults should aim for:

1. At least 150 minutes of moderate-intensity aerobic exercise (such as brisk walking) per week, or 75 minutes of vigorous-intensity aerobic exercise (such as running) per week, or a combination of both. These can be broken down into shorter sessions, such as 30 minutes a day for 5 days a week.
2. Muscle-strengthening activities that involve all major muscle groups (legs, hips, back, abdomen, chest, shoulders, and arms) should be done at least two days per week.

Remember that these are general guidelines and individual needs may vary depending on factors like age, fitness level, and health conditions. Always consult with a healthcare professional before starting a new exercise program, especially if you have any medical concerns or if you are not used to physical activity.

***

**Should I do cardio or lift weights to prevent heart disease?**

***

I am not a doctor, but I can provide you with some general information on the topic. To prevent heart disease, it is generally recommended to engage in both cardio and resistance training exercises as part of a well-rounded exercise program.

Cardiovascular exercises, such as brisk walking, running, swimming, or cycling, help improve heart and lung function, increase good cholesterol (HDL), lower blood pressure, and decrease the risk of heart disease.

Resistance training, or lifting weights, can also have positive effects on heart health. It can help lower blood pressure, improve cholesterol levels, and increase insulin sensitivity, among other benefits.

Ultimately, it's essential to consult with your healthcare provider before starting any exercise program, especially if you have pre-existing conditions or concerns. They can help you design a workout routine tailored to your specific needs and goals, which may include a combination of cardio and resistance training.

***

I am not a doctor, but I can provide you with general information on exercise and heart health. Both cardio and resistance training have been shown to be beneficial for heart health, so incorporating both types of exercise into your routine is likely a good idea.

Cardiovascular (cardio) exercises, such as walking, jogging, swimming, or cycling, increase your heart rate and improve blood flow. They can help improve your heart's efficiency, lower blood pressure, and reduce your risk of heart disease.

Resistance (weight) training, such as lifting weights or doing bodyweight exercises, can also contribute to better heart health. It can help increase muscle strength, improve blood sugar control, and boost HDL (good) cholesterol.

For optimal heart health benefits, the American Heart Association recommends at least 150 minutes of moderate-intensity aerobic activity or 75 minutes of vigorous aerobic activity per week, or a combination of both, preferably spread throughout the week. Additionally, they recommend engaging in moderate to high-intensity muscle-strengthening activities at least two days per week.

Always consult with a healthcare professional before starting a new exercise program, especially if you have any pre-existing conditions or concerns. They can help you create a personalized exercise plan that is safe and effective for your specific needs.

***

I'm not a doctor, but I can provide some general information on exercise and heart disease prevention. Both cardio and weightlifting can provide benefits for heart health, but they work in different ways.

Cardio, or aerobic exercise, is often recommended for preventing heart disease because it helps improve cardiovascular endurance, lowers blood pressure, increases good cholesterol (HDL), and can help with weight management. Some examples of cardio exercises are running, swimming, cycling, or brisk walking.

Weightlifting, or resistance training, can also provide benefits for heart health. It helps increase muscle mass, which can boost metabolism and aid in weight management. It also improves muscular strength and endurance, which can contribute to better overall cardiovascular health.

Incorporating both types of exercise into a well-rounded fitness routine may be the most effective way to prevent heart disease. The American Heart Association recommends at least 150 minutes of moderate-intensity aerobic exercise or 75 minutes of vigorous aerobic exercise per week, along with muscle-strengthening activities at least two days per week.

However, it's important to consult with your healthcare professional before starting any new exercise program, especially if you have a history of heart problems or other health concerns. They can provide personalized guidance on the best approach for your specific situation.

***

**How can I lose weight?**

***

Losing weight typically involves a combination of healthy eating, regular physical activity, and lifestyle changes. Here are some tips to help you lose weight:

1. Set realistic goals: Start with achievable targets, such as losing 1-2 pounds per week. Gradual weight loss is more sustainable and healthier than trying to lose weight quickly.
2. Monitor your calorie intake: Keep track of the calories you consume daily. To lose weight, you need to create a calorie deficit by burning more calories than you consume. Use an online calculator to determine your daily calorie needs based on your age, sex, weight, and activity level.
3. Eat a balanced diet: Focus on consuming nutrient-dense foods, such as fruits, vegetables, lean proteins, whole grains, and healthy fats. Avoid or minimize the intake of processed foods, sugary drinks, and unhealthy snacks.
4. Control portion sizes: Overeating, even healthy foods, can lead to weight gain. Use smaller plates, measure servings, and listen to your hunger cues to avoid overeating.
5. Stay hydrated: Drink plenty of water throughout the day. Staying hydrated can help control your appetite and improve digestion.
6. Increase physical activity: Aim for at least 150 minutes of moderate-intensity aerobic exercise or 75 minutes of vigorous-intensity aerobic exercise per week. Include strength training exercises at least two days per week.
7. Get enough sleep: Aim for 7-9 hours of sleep per night. Poor sleep can lead to weight gain by affecting your hunger hormones and increasing cravings for unhealthy foods.
8. Manage stress: High stress levels can lead to emotional eating and weight gain. Practice stress management techniques, such as meditation, yoga, or deep breathing exercises.
9. Stay consistent: Weight loss takes time, and consistency is key. Stick to your healthy habits, and don't get discouraged by minor setbacks.
10. Seek support: Share your weight loss goals with friends, family, or a weight loss support group. Having a support system can help keep you motivated and accountable.

Remember, it's important to consult a healthcare professional before starting any weight loss plan, especially if you have any pre-existing health conditions or concerns.

***

Losing weight generally involves a combination of a healthy diet, regular physical activity, and lifestyle modifications. Here are some tips to help you lose weight safely and effectively:

1. Set realistic goals: Start by setting achievable weight loss goals, such as losing 1-2 pounds per week. Gradual weight loss is more sustainable and healthier than drastic measures.
2. Eat a balanced diet: Focus on consuming nutrient-dense foods, including fruits, vegetables, whole grains, lean protein, and healthy fats. Limit processed foods, added sugars, and excessive amounts of unhealthy fats.
3. Control portion sizes: Be mindful of portion sizes, as overeating even healthy foods can contribute to weight gain. Use smaller plates, measure out servings, and listen to your body's hunger and fullness cues.
4. Exercise regularly: Aim for at least 150 minutes of moderate-intensity aerobic exercise or 75 minutes of vigorous-intensity aerobic exercise per week, along with muscle-strengthening activities on two or more days per week.
5. Stay hydrated: Drink water throughout the day to stay hydrated and help control your appetite.
6. Get enough sleep: Aim for 7-9 hours of sleep per night, as inadequate sleep can negatively impact weight loss efforts.
7. Manage stress: Find healthy ways to cope with stress, such as meditation, yoga, or deep breathing exercises, as stress can contribute to weight gain.
8. Keep a food and exercise diary: Tracking your food intake and physical activity can help you stay accountable and identify patterns that may be hindering your weight loss.
9. Be consistent: Maintain your healthy habits, even during weekends or special occasions, to help ensure long-term success.
10. Seek support: Enlist the help of friends, family, or a professional, such as a registered dietitian or personal trainer, to support you in your weight loss journey.

Remember, it's essential to consult with a healthcare professional before starting any weight loss plan, as they can provide personalized guidance based on your specific needs and medical history.

***

Losing weight typically involves a combination of healthy eating, regular physical activity, and lifestyle modifications. Here are some general tips to help you lose weight:

1. Set realistic goals: Aim for a gradual, steady weight loss of about 1-2 pounds (0.5-1 kg) per week.
2. Eat a balanced diet: Focus on consuming whole, nutrient-dense foods like fruits, vegetables, lean proteins, whole grains, and healthy fats. Reduce your intake of added sugars, processed foods, and unhealthy fats.
3. Control portion sizes: Be mindful of portion sizes and serving recommendations to avoid overeating.
4. Stay hydrated: Drink plenty of water throughout the day to stay hydrated and help control hunger.
5. Exercise regularly: Aim for at least 150 minutes of moderate-intensity aerobic exercise or 75 minutes of vigorous-intensity aerobic exercise per week, along with muscle-strengthening activities on two or more days per week.
6. Sleep well: Aim for 7-9 hours of quality sleep each night, as poor sleep can contribute to weight gain.
7. Manage stress: Practice stress reduction techniques like meditation, yoga, or deep breathing exercises to help manage emotional eating.
8. Track your progress: Keep a food diary or use a mobile app to track your food intake, exercise, and weight changes. Monitoring your habits can help you make adjustments as needed.
9. Seek support: Consider joining a weight loss group or enlisting the help of friends, family, or a professional (e.g., dietitian, personal trainer) for support and accountability.
10. Be patient and persistent: Weight loss takes time, and it's important to stay consistent with your efforts. Remember that setbacks are a normal part of the process and don't let them discourage you.

Always consult with a healthcare professional before beginning any weight loss plan, especially if you have any pre-existing medical conditions or concerns.

***

**How can I decrease LDL?**

***

Lowering your LDL (low-density lipoprotein), often referred to as "bad cholesterol," can help reduce your risk of heart disease and stroke. Here are some lifestyle changes and approaches to help decrease your LDL levels:

1. Improve your diet: Adopt a heart-healthy diet that includes:
   - Eating more fruits, vegetables, whole grains, and legumes
   - Consuming lean protein sources such as fish, poultry, and plant-based proteins
   - Limiting saturated and trans fats found in red meat, full-fat dairy products, and processed foods
   - Choosing healthier fats such as those found in olive oil, avocado, and nuts
   - Reducing added sugars and refined carbohydrates
2. Exercise regularly: Aim for at least 150 minutes of moderate-intensity aerobic exercise or 75 minutes of vigorous-intensity aerobic exercise per week, along with muscle-strengthening activities on two or more days a week.
3. Maintain a healthy weight: Losing excess weight can help lower your LDL cholesterol levels. A combination of a healthy diet and regular physical activity is key to achieving and maintaining a healthy weight.
4. Stop smoking: If you smoke, quitting can help improve your cholesterol levels and overall heart health.
5. Limit alcohol consumption: Moderate alcohol intake (one drink per day for women and up to two drinks per day for men) can have some heart benefits, but excessive alcohol consumption can raise your LDL levels and harm your heart.
6. Manage stress: Chronic stress can contribute to unhealthy habits that may increase your LDL cholesterol. Develop healthy stress management techniques such as exercise, meditation, or seeking support from friends, family, or a mental health professional.

If lifestyle changes alone aren't enough to lower your LDL cholesterol to a safe level, your healthcare provider may recommend medications such as statins, bile acid sequestrants, cholesterol absorption inhibitors, or PCSK9 inhibitors. It's essential to follow your healthcare provider's recommendations and maintain regular check-ups to monitor your cholesterol levels.

***

I am not a doctor, but I can provide you with some general information on how to decrease LDL (low-density lipoprotein) cholesterol, commonly known as "bad" cholesterol. High levels of LDL cholesterol can contribute to plaque buildup in your arteries, increasing the risk of heart disease and stroke. Here are some lifestyle changes that may help lower your LDL cholesterol:

1. Diet:
   - Eat a heart-healthy diet, focusing on fruits, vegetables, whole grains, and legumes.
   - Limit saturated and trans fats, found in fatty meats, full-fat dairy products, and many processed foods.
   - Choose healthier fats like monounsaturated and polyunsaturated fats, found in olive oil, canola oil, nuts, and fatty fish.
   - Increase your fiber intake, especially soluble fiber, found in foods like oats, beans, and apples.
   - Consume plant-based protein sources like beans, lentils, and tofu instead of animal protein.
2. Exercise:
   - Engage in regular physical activity, such as brisk walking, swimming, or cycling, for at least 150 minutes per week.
   - Include strength training exercises in your routine, like lifting weights or doing bodyweight exercises, at least twice a week.
   - Consider incorporating high-intensity interval training (HIIT) workouts to help improve your cardiovascular health.
3. Maintain a healthy weight:
   - Losing excess weight can help lower LDL cholesterol levels.
   - Focus on a balanced diet and regular exercise to achieve and maintain a healthy weight.
4. Quit smoking:
   - Smoking can increase LDL cholesterol levels and harm your cardiovascular health. Quitting smoking can help improve your cholesterol profile and overall heart health.
5. Limit alcohol consumption:
   - Moderate alcohol intake is generally considered safe, but excessive drinking can raise LDL cholesterol levels. Limit your alcohol consumption to one drink per day for women and two drinks per day for men.
6. Manage stress:
   - Chronic stress can negatively impact your cholesterol levels. Practice stress management techniques like meditation, deep breathing, or yoga.
7. Consider medication:
   - If lifestyle changes are not sufficient to lower your LDL cholesterol levels, consult your healthcare provider. They may recommend medication, such as statins, to help manage your cholesterol.

It is essential to consult a healthcare professional for personalized advice, as they can provide recommendations based on your individual needs and health history.

***

I'm not a doctor, but I can offer some general advice on how to decrease LDL (low-density lipoprotein), often referred to as "bad" cholesterol. High levels of LDL cholesterol are associated with an increased risk of heart disease and stroke. Here are some lifestyle changes that might help lower your LDL levels:

1. Eat a heart-healthy diet:
   - Consume more fiber-rich foods such as fruits, vegetables, whole grains, and legumes.
   - Opt for healthy fats, like those found in olive oil, nuts, seeds, and avocados.
   - Limit saturated fats found in red meat, full-fat dairy products, and processed foods.
   - Avoid trans fats, which are found in some fried and packaged foods.
2. Maintain a healthy weight:
   - Losing excess weight can help lower your LDL levels.
   - Aim for a gradual weight loss through a balanced diet and regular exercise.
3. Exercise regularly:
   - Aim for at least 150 minutes of moderate-intensity aerobic exercise or 75 minutes of vigorous-intensity aerobic exercise per week.
   - Incorporate resistance training, such as weight lifting, into your routine.
4. Limit alcohol intake:
   - Moderate alcohol consumption is defined as up to one drink per day for women and up to two drinks per day for men.
   - Excessive alcohol consumption can raise LDL levels and increase the risk of heart disease.
5. Don't smoke:
   - Quitting smoking can help improve your lipid profile, including LDL levels.
6. Consider dietary supplements:
   - Some supplements, such as omega-3 fatty acids, niacin, and plant sterols, may help lower LDL levels. However, it's important to consult your healthcare provider before starting any new supplement regimen.
7. Monitor and manage stress:
   - Chronic stress can negatively affect your overall health, including your cholesterol levels. Practice stress-reducing techniques like meditation, yoga, or deep breathing exercises.

Always consult your healthcare provider before making any significant changes to your diet or exercise routine, especially if you have any pre-existing conditions or are on medication. Your healthcare provider can help you develop a personalized plan to lower your LDL levels and maintain overall heart health.

***

**How can I decrease triglycerides?**

***

I'm not a doctor, but I can provide you with some general advice on how to potentially decrease triglycerides. It's important to consult with a healthcare professional before making any significant changes to your lifestyle or diet. That being said, here are some tips that might help:

1. Maintain a healthy weight: Excess body fat may contribute to high triglycerides. Losing weight through a combination of diet and exercise can help reduce triglyceride levels.
2. Eat a balanced diet: Focus on whole foods, such as fruits, vegetables, whole grains, lean proteins, and healthy fats like those found in nuts, seeds, and fish. Limit your intake of refined carbohydrates, added sugars, and trans fats, as these can contribute to high triglyceride levels.
3. Limit sugar intake: Consuming too much sugar can lead to increased triglyceride levels. Aim to limit your intake of sugary beverages, snacks, and processed foods.
4. Choose healthier fats: Opt for unsaturated fats found in foods like olive oil, avocados, nuts, and fatty fish, and limit saturated fats found in red meat, full-fat dairy products, and processed foods.
5. Increase your intake of omega-3 fatty acids: Consuming omega-3 fatty acids, found in fish like salmon, mackerel, and sardines, as well as in flaxseeds, chia seeds, and walnuts, can help lower triglyceride levels.
6. Exercise regularly: Engaging in regular physical activity can help reduce triglyceride levels. Aim for at least 150 minutes of moderate-intensity aerobic exercise or 75 minutes of vigorous-intensity aerobic exercise per week, along with muscle-strengthening activities on two or more days per week.
7. Limit alcohol consumption: Alcohol can raise triglyceride levels, so it's important to moderate your intake or avoid it altogether.
8. Quit smoking: Smoking can contribute to higher triglyceride levels and other health problems. Quitting smoking can improve your overall health and potentially lower your triglyceride levels.
9. Manage stress: Chronic stress may contribute to high triglyceride levels. Practicing stress management techniques such as deep breathing exercises, meditation, and yoga can help you better manage stress and potentially improve your triglyceride levels.
10. Consider medication: In some cases, lifestyle changes may not be enough to reduce triglyceride levels. Your doctor may prescribe medication to help lower your triglycerides if necessary.

Remember, it's crucial to consult with a healthcare professional to discuss your personal situation and determine the most appropriate course of action.

***

I'm not a doctor, but I can provide you with some general information on how to potentially decrease triglycerides. Always consult a healthcare professional before making any significant changes to your lifestyle or diet.

1. Eat a healthy diet:
   - Focus on consuming whole foods, such as fruits, vegetables, whole grains, lean proteins, and healthy fats.
   - Reduce intake of simple sugars, refined carbohydrates, and unhealthy fats (trans and saturated fats).
   - Include foods rich in omega-3 fatty acids, such as fatty fish (e.g., salmon, mackerel, sardines), flaxseeds, chia seeds, and walnuts.
2. Exercise regularly:
   - Aim for at least 150 minutes of moderate-intensity aerobic activity or 75 minutes of vigorous-intensity aerobic activity per week.
   - Include strength training exercises for all major muscle groups at least two days per week.
3. Maintain a healthy weight:
   - If you are overweight or obese, losing even a small amount of weight can help lower triglyceride levels.
4. Limit alcohol consumption:
   - Alcohol can raise triglyceride levels, so it's important to moderate your intake or avoid it altogether if necessary.
5. Quit smoking:
   - Smoking can contribute to elevated triglyceride levels and an increased risk of heart disease.
6. Control blood sugar levels:
   - If you have diabetes or prediabetes, managing your blood sugar can help reduce triglyceride levels.
7. Consider medication or supplements:
   - In some cases, your healthcare provider may recommend medications or supplements, such as niacin, fibrates, or fish oil, to help lower triglyceride levels. Always consult a healthcare professional before starting any new medications or supplements.

Remember to consult with a healthcare professional for personalized advice and recommendations tailored to your specific needs and health conditions.

***

I'm not a doctor, but I can offer some general advice on how to potentially decrease triglycerides. It's important to consult with a healthcare professional for personalized recommendations. That being said, here are some lifestyle changes you might consider:

1. Improve your diet:
   - Choose healthier fats: Opt for monounsaturated and polyunsaturated fats found in olive oil, canola oil, and nuts, while avoiding trans fats and saturated fats.
   - Increase fiber intake: Eat more whole grains, fruits, vegetables, and legumes.
   - Limit sugar intake: Reduce consumption of added sugars and refined carbohydrates.
   - Focus on lean protein: Consume lean protein sources like fish, poultry, beans, and low-fat dairy products.
   - Avoid alcohol: Reduce or eliminate alcohol consumption.
2. Exercise regularly: Aim for at least 150 minutes of moderate-intensity aerobic exercise or 75 minutes of vigorous-intensity exercise per week, along with strength training exercises at least twice a week.
3. Lose weight: If you're overweight or obese, losing even a modest amount of weight can help lower triglyceride levels.
4. Manage existing health conditions: If you have other health conditions like diabetes or hypothyroidism, work closely with your healthcare provider to manage them, as they can contribute to elevated triglyceride levels.
5. Quit smoking: If you smoke, quitting can help improve your overall cardiovascular health, including lowering triglyceride levels.
6. Consider medications: In some cases, your healthcare provider may recommend medications to help lower triglyceride levels, especially if lifestyle changes aren't enough or if you have other risk factors for heart disease.

Remember to consult with a healthcare professional before making any significant changes to your diet or exercise routine. They can help you develop a personalized plan tailored to your needs and monitor your progress.

***

**What is lipoprotein(a)?**

***

Lipoprotein(a), abbreviated as Lp(a), is a type of lipoprotein found in the blood. It is a complex particle made up of a low-density lipoprotein (LDL) particle and a specific protein called apolipoprotein(a). The structure of Lp(a) is similar to that of LDL, which is often referred to as "bad" cholesterol because high levels of LDL in the blood can lead to the buildup of plaque in the arteries and increase the risk of cardiovascular diseases.

Lp(a) is believed to have atherogenic properties, meaning it can contribute to the development of atherosclerosis, a condition where plaque builds up inside the arteries, narrowing them and restricting blood flow. Elevated Lp(a) levels are associated with an increased risk of coronary heart disease, stroke, and peripheral artery disease.

The concentration of Lp(a) in the blood is largely determined by genetics, and it can vary significantly among individuals. There is currently no specific treatment for high Lp(a) levels, but lifestyle modifications, such as a healthy diet, regular exercise, and maintaining a healthy weight, can help lower the overall risk of cardiovascular diseases. In some cases, medications like statins or PCSK9 inhibitors may be prescribed to help lower LDL cholesterol levels and reduce cardiovascular risk, but their effects on Lp(a) are limited.

***

Lipoprotein(a), often abbreviated as Lp(a), is a type of lipoprotein found in the blood. It is a complex molecule made up of a low-density lipoprotein (LDL) particle and an additional protein called apolipoprotein(a) bound to it. LDL is commonly known as "bad cholesterol" because high levels of it can lead to plaque buildup in the arteries, increasing the risk of cardiovascular diseases like heart attack and stroke.

Lp(a) shares structural similarities with LDL, but its specific role in the body and contribution to cardiovascular risk is not completely understood. Elevated Lp(a) levels have been associated with an increased risk of cardiovascular diseases, such as coronary artery disease, atherosclerosis, and stroke. Some studies suggest that Lp(a) may promote the formation of blood clots and contribute to the development of atherosclerotic plaques.

Lp(a) levels in the blood are largely determined by genetics, and there is no specific dietary or lifestyle intervention known to substantially lower Lp(a) levels. However, maintaining a healthy lifestyle, including a balanced diet, regular exercise, and avoiding tobacco products, can help manage overall cardiovascular risk. In some cases, doctors may prescribe medications like niacin or PCSK9 inhibitors to reduce Lp(a) levels in high-risk patients.

***

Lipoprotein(a), often abbreviated as Lp(a), is a type of lipoprotein found in the blood. It is structurally similar to low-density lipoprotein (LDL), which is commonly referred to as "bad cholesterol." Lipoproteins are complex particles composed of lipids (fats) and proteins, and they play a critical role in transporting cholesterol and other lipids through the bloodstream.

Lp(a) consists of an LDL-like particle bound to a specific protein called apolipoprotein(a) or apo(a). The primary function of Lp(a) is not completely understood, but it is thought to be involved in various physiological processes such as blood clotting and wound healing.

Elevated levels of Lp(a) have been associated with an increased risk of cardiovascular diseases, including coronary artery disease, heart attacks, and strokes. High Lp(a) levels can be influenced by both genetic factors and lifestyle factors, although the genetic component is considered to be the primary determinant.

There is no specific treatment to lower Lp(a) levels, but managing other cardiovascular risk factors, such as maintaining a healthy diet, exercising regularly, and controlling blood pressure and cholesterol levels, can help reduce the overall risk of cardiovascular disease. In some cases, physicians may prescribe medications like statins, PCSK9 inhibitors, or niacin to help manage the risk associated with high Lp(a) levels, although their effectiveness in directly lowering Lp(a) is limited.

***

**How can I quit smoking?**

***

Quitting smoking is a great decision for your health and well-being, but it can be challenging. Here are some strategies to help you quit:

1. Make a plan: Set a quit date, and stick to it. This gives you time to mentally prepare and gather resources for support.
2. Identify your triggers: Recognize situations or emotions that make you want to smoke, and develop strategies to deal with them.
3. Get support: Reach out to friends, family, or support groups to help you stay motivated and accountable.
4. Use nicotine replacement therapy (NRT): Products like nicotine gum, patches, lozenges, and inhalers can help manage withdrawal symptoms and cravings.
5. Consider prescription medications: Talk to your doctor about prescription medications like varenicline (Chantix) or bupropion (Zyban), which can help reduce cravings and withdrawal symptoms.
6. Stay active: Exercise can help distract you from cravings and improve your mood. It also has health benefits that can counteract the negative effects of smoking.
7. Practice stress management: Find healthier ways to deal with stress, such as deep breathing, meditation, or yoga.
8. Change your routines: Alter habits or routines that are associated with smoking, such as taking a different route to work or avoiding places where you used to smoke.
9. Stay away from alcohol and other triggers: Alcohol and certain situations can weaken your resolve to quit. Avoid them, especially in the early stages of quitting.
10. Reward yourself: Set milestones for quitting, and reward yourself for reaching them. This will help keep you motivated.
11. Stay patient and persistent: Quitting smoking can be difficult, and relapses are common. Don't get discouraged; just keep trying.

Remember, everyone's journey to quitting smoking is different. Be patient, and don't hesitate to seek professional help if needed.

***

Quitting smoking is a challenging but highly rewarding endeavor. Here are some steps and strategies to help you quit:

1. Make a plan: Set a quit date, and stick to it. This gives you time to mentally prepare and gather the necessary resources and support.
2. Identify your triggers: Recognize the situations, places, or feelings that make you want to smoke. By understanding your triggers, you can better avoid or manage them.
3. Seek support: Talk to friends, family, or a support group about your decision to quit. They can offer encouragement and help keep you accountable.
4. Consider nicotine replacement therapy (NRT): Nicotine gum, patches, lozenges, inhalers, or nasal sprays can help ease withdrawal symptoms and cravings. Consult with a healthcare professional before starting NRT.
5. Medications: Certain prescription medications, such as bupropion (Zyban) and varenicline (Chantix), can help reduce cravings and withdrawal symptoms. Talk to your doctor about whether these options are suitable for you.
6. Behavioral therapy: Counseling, either individually or in a group setting, can help you develop coping strategies and provide support during your quit journey.
7. Stay active: Regular exercise can help reduce cravings and improve your mood, making it easier to resist the urge to smoke.
8. Manage stress: Find healthier ways to cope with stress, such as meditation, deep breathing exercises, or yoga.
9. Create new habits: Replace smoking with healthier habits, like drinking water, chewing gum, or engaging in a hobby when cravings strike.
10. Reward yourself: Celebrate your milestones, no matter how small. Use the money you save from not buying cigarettes to treat yourself to something special.
11. Be prepared for setbacks: Understand that quitting is a process, and it's normal to have setbacks. Don't be too hard on yourself if you slip up. Instead, learn from the experience and keep pushing forward.

Remember, quitting smoking is a journey, and it may take several attempts before you successfully quit for good. Stay persistent and keep trying different strategies until you find what works best for you.

***

Quitting smoking is a challenging but highly rewarding process. It can greatly improve your health and overall quality of life. Here are some strategies to help you quit smoking:

1. Make a plan: Set a quit date and stick to it. Tell friends and family about your decision and ask for their support. Remove all smoking-related items (cigarettes, lighters, ashtrays) from your environment.
2. Consider nicotine replacement therapy (NRT): NRT helps to reduce nicotine withdrawal symptoms and cravings. Options include nicotine patches, gum, lozenges, inhalers, and nasal sprays. Consult your doctor before starting NRT to determine the best option for you.
3. Prescription medications: Talk to your doctor about prescription medications like varenicline (Chantix) or bupropion (Zyban) that can help reduce cravings and withdrawal symptoms.
4. Behavioral therapy: Cognitive-behavioral therapy (CBT) can help you identify and change your thought patterns and behaviors related to smoking. A trained counselor or therapist can help you develop coping strategies to deal with cravings and other triggers.
5. Support groups and helplines: Join a local or online support group for people trying to quit smoking, or call a quitline for advice and encouragement.
6. Exercise: Regular physical activity can help reduce nicotine cravings and improve your mood.
7. Stay busy: Fill your time with activities that make it difficult to smoke, such as spending time with non-smoking friends and family, engaging in hobbies, or pursuing new interests.
8. Manage stress: Find healthy ways to cope with stress, such as meditation, yoga, or deep breathing exercises.
9. Reward yourself: Celebrate milestones and reward yourself with treats or experiences to reinforce your commitment to quitting.
10. Stay persistent: Quitting smoking is tough, and relapses can happen. If you slip up, don't be too hard on yourself. Learn from your mistakes and refocus on your goal to quit smoking.

Remember, quitting smoking is a process, and it may take several attempts before you succeed. Stay patient and persistent, and don't hesitate to seek help from professionals or support networks.

***

**What are the side effects of statins?**

***

Statins are a class of medications used to lower cholesterol levels in the blood, particularly low-density lipoprotein (LDL) cholesterol, which is often referred to as "bad cholesterol." They can help reduce the risk of heart attacks, strokes, and other cardiovascular events. While statins are generally well-tolerated, some people may experience side effects. Common and less common side effects include:

1. Muscle pain and weakness: Some people experience muscle aches, tenderness, or weakness while taking statins. In rare cases, statins can cause a severe form of muscle damage called rhabdomyolysis.
2. Liver damage: Statins can cause elevated liver enzymes in some people, which may indicate liver damage. Serious liver injury is rare, but your doctor may monitor your liver function periodically.
3. Increased blood sugar or type 2 diabetes: Statins can slightly raise blood sugar levels, potentially increasing the risk of developing type 2 diabetes in some individuals.
4. Memory issues or confusion: Some people report memory loss, confusion, or other cognitive issues while taking statins, though these side effects are rare and not well-established.
5. Digestive issues: Some individuals may experience gastrointestinal side effects, such as nausea, diarrhea, or constipation.
6. Rash or flushing: A skin rash or flushing may occur in some people taking statins.
7. Headaches: Headaches are another possible side effect of statin use.
8. Sleep disturbances: Some people report difficulty sleeping or experiencing vivid dreams while on statins.

It's important to remember that the benefits of statins in reducing cardiovascular risk usually outweigh the potential side effects for most people. However, if you experience any concerning symptoms while taking statins, you should discuss them with your healthcare provider. They may adjust your dosage or consider switching you to a different medication.

***

Statins are a class of medications used to lower cholesterol levels in the blood, reducing the risk of cardiovascular diseases such as heart attack and stroke. While statins are generally safe and well-tolerated, they can cause side effects in some people. Common side effects include:

1. Muscle pain and weakness: This is the most common side effect of statins, and it can range from mild muscle aches to more severe muscle pain and weakness (myopathy). In rare cases, statins can cause a serious, life-threatening muscle breakdown called rhabdomyolysis.
2. Liver damage: Statins may cause an increase in liver enzymes, indicating potential liver damage. This is usually mild and temporary, but in rare cases, it can lead to liver failure.
3. Increased blood sugar levels: Statins may slightly raise blood sugar levels, which can increase the risk of developing type 2 diabetes.
4. Gastrointestinal issues: Some people may experience nausea, gas, diarrhea, or constipation while taking statins.
5. Headaches and dizziness: These are less common side effects but can occur in some people.
6. Skin reactions: Rash, itchiness, or flushing may occur in some individuals.
7. Memory problems and confusion: Some studies have suggested a possible link between statins and cognitive issues, but the evidence is inconclusive.
8. Peripheral neuropathy: This is a rare side effect characterized by nerve damage that can cause tingling, numbness, or pain in the hands and feet.

It's important to note that the risk of side effects varies depending on the specific statin, the dose, and individual factors. If you are concerned about side effects or believe you may be experiencing them, speak with your healthcare provider. They can help determine whether the benefits of the medication outweigh the risks for you and may adjust your treatment plan accordingly.

***

Statins are a class of drugs commonly prescribed to lower cholesterol levels, particularly low-density lipoprotein (LDL) cholesterol, which is associated with an increased risk of heart disease and stroke. While statins can be effective in reducing cardiovascular risk, they may also have some side effects. It's important to note that not everyone experiences these side effects, and for many people, the benefits of statins outweigh the risks.

Some potential side effects of statins include:

1. Muscle pain and weakness (myalgia): This is the most common side effect of statins. It can range from mild discomfort to severe pain and may interfere with daily activities.
2. Increased liver enzymes: Statins can cause an increase in liver enzymes, which could indicate liver damage. However, this is usually temporary and reversible. Your doctor may monitor your liver function periodically.
3. Memory problems and confusion: Some people have reported memory loss or confusion while taking statins. However, this side effect is not well understood, and more research is needed to establish a clear connection.
4. Type 2 diabetes: Statin use has been associated with a slightly increased risk of developing type 2 diabetes, especially in people who already have risk factors for the disease.
5. Gastrointestinal issues: Some people may experience stomach pain, constipation, diarrhea, or nausea while taking statins.
6. Rash or flushing: Statins can occasionally cause a skin rash or flushing, usually mild and temporary.
7. Headaches and dizziness: These symptoms are relatively rare but can occur in some people taking statins.
8. Increased risk of myopathy and rhabdomyolysis: In rare cases, statins can cause myopathy, which is muscle inflammation and damage, and rhabdomyolysis, a severe form of myopathy that can lead to kidney damage and failure. The risk of these conditions is higher with higher doses of statins or when taken in combination with certain other medications.

If you experience any side effects while taking statins, it's important to discuss them with your healthcare provider. They may adjust your dosage, recommend a different type of statin, or consider alternative treatment options if necessary. Remember that the benefits of statins in reducing the risk of heart disease and stroke often outweigh the potential side effects for many people.

***

**I have muscle pain with a statin. What should I do?**

***

I am not a doctor, but I can offer some general advice if you're experiencing muscle pain while taking a statin. It's important to consult with your healthcare provider to discuss your symptoms, as they can provide personalized advice based on your medical history and current health.

In the meantime, here are a few general suggestions:

1. Inform your doctor: Contact your healthcare provider to discuss the muscle pain you're experiencing. They may want to adjust your medication or recommend an alternative.
2. Monitor your symptoms: Keep track of when the pain occurs, its intensity, and any other accompanying symptoms. This information can be helpful for your doctor in determining the cause and possible solutions.
3. Consider lifestyle changes: Eating a healthy diet, exercising regularly, and maintaining a healthy weight may help improve your cholesterol levels and reduce your need for statin medications. Talk to your doctor about which lifestyle changes may be most effective for you.
4. Over-the-counter pain relief: With your doctor's approval, you may be able to take over-the-counter pain relievers to help manage muscle pain. Always follow the recommended dosage and talk to your doctor before starting any new medications.
5. Stay hydrated: Drinking plenty of water can help reduce muscle pain and cramps. Make sure you're getting enough fluids throughout the day.

Remember, it's crucial to consult with your healthcare provider about any concerns you have while taking statin medications. They can offer the most appropriate guidance and recommendations based on your unique circumstances.

***

I'm not a doctor, but I can try to help you with some general information. If you're experiencing muscle pain while taking a statin, it's important to speak with your healthcare provider as soon as possible. They can assess your symptoms, determine if the statin is the cause, and discuss potential solutions. These may include adjusting the dosage, switching to a different statin, or considering alternative treatments.

Do not stop taking your medication without consulting your healthcare provider, as this can have serious consequences.

Remember, it's important to discuss your symptoms and concerns with a healthcare professional who can provide personalized advice based on your medical history and current medications.

***

I am not a doctor, but I can provide some general advice if you're experiencing muscle pain while taking a statin. It's important to consult with your healthcare professional for personalized advice, as they can best assess your individual situation and provide appropriate recommendations. Here are some general suggestions:

1. Report your symptoms to your doctor: Inform your healthcare provider about the muscle pain you're experiencing. They may decide to adjust your dosage or switch to a different statin or medication.
2. Temporarily stop the statin: Your doctor might advise you to stop taking the statin temporarily to see if your muscle pain improves. Always follow your doctor's instructions regarding stopping or starting any medication.
3. Take a lower dosage: In some cases, taking a lower dosage of the statin may help reduce muscle pain. Consult with your healthcare provider about adjusting your dosage.
4. Switch to another statin: Different statins have varying potencies and side effect profiles. Your doctor might recommend trying a different statin that may be better tolerated.
5. Consider alternative medications: If statins continue to cause muscle pain, your doctor may recommend alternative cholesterol-lowering medications, such as ezetimibe or PCSK9 inhibitors.
6. Maintain a healthy lifestyle: Eating a balanced diet, exercising regularly, maintaining a healthy weight, and not smoking can help lower your cholesterol and reduce your risk of heart disease.

Remember, always consult with your healthcare provider before making any changes to your medication or lifestyle.

***

**My cholesterol is still high and I’m already on a statin. What should I do?**

***

I am not a doctor, but I can provide some general advice that you might consider discussing with a healthcare professional. If your cholesterol levels remain high despite being on a statin, there are several potential steps you could take:

1. Consult your doctor: Speak with your healthcare provider about your current statin dose, any side effects you're experiencing, and other potential treatments. They may adjust your medication, prescribe an additional medication, or recommend lifestyle changes.
2. Lifestyle modifications: Adopting a heart-healthy lifestyle can help improve your cholesterol levels. This may include:
   - Eating a healthy diet, rich in fruits, vegetables, whole grains, and lean proteins.
   - Reducing saturated and trans fats, as well as added sugars in your diet.
   - Increasing soluble fiber and omega-3 fatty acids consumption.
   - Engaging in regular physical activity, such as at least 150 minutes of moderate-intensity aerobic exercise per week.
   - Maintaining a healthy weight.
   - Quitting smoking, if you're a smoker.
3. Consider other medications: If your cholesterol levels don't improve with lifestyle changes and statins, your doctor may recommend additional medications, such as ezetimibe, bile acid sequestrants, or PCSK9 inhibitors.
4. Monitor your progress: Keep track of your cholesterol levels and discuss your progress with your doctor during regular checkups. This will help you and your doctor determine if further adjustments to your treatment plan are needed.

Please consult with your healthcare provider before making any changes to your medication or lifestyle, as they can provide personalized guidance based on your medical history and current health situation.

***

I'm not a doctor, but I can offer you some general suggestions to consider if you have high cholesterol despite being on a statin. It's important to discuss your specific situation with a healthcare professional, as they can give you tailored advice.

1. Reevaluate your diet: Focus on consuming a heart-healthy diet that's low in saturated fat, trans fat, and cholesterol. Incorporate more fruits, vegetables, whole grains, legumes, and healthy fats from sources like olive oil, avocados, and nuts.
2. Increase physical activity: Aim for at least 150 minutes of moderate-intensity aerobic exercise per week, or 75 minutes of vigorous-intensity aerobic exercise, spread across most days of the week.
3. Maintain a healthy weight: If you're overweight, losing weight can help lower your cholesterol levels. Aim for gradual, sustainable weight loss through a balanced diet and regular physical activity.
4. Stop smoking: Smoking can decrease your HDL (good) cholesterol and increase your LDL (bad) cholesterol. Quitting can improve your cholesterol levels and overall heart health.
5. Limit alcohol consumption: Moderate alcohol consumption may have some health benefits, but excessive alcohol intake can lead to higher cholesterol levels. Stick to the recommended guidelines, which are typically no more than one drink per day for women and two drinks per day for men.
6. Manage stress: Chronic stress may contribute to high cholesterol levels. Practice stress reduction techniques like mindfulness, meditation, or yoga to help manage stress.
7. Review your medications: Consult with your doctor about your current statin medication. They may consider adjusting the dose, switching to a different statin, or adding another cholesterol-lowering medication to your treatment plan.

Remember, it's crucial to consult with your healthcare provider before making any changes to your medication, diet, or exercise routine. They can provide personalized guidance and monitor your progress.

***

I'm not a doctor, but I can try to provide some general information that might help you. If your cholesterol remains high despite being on a statin, there are several potential options to consider. You should discuss these with your healthcare professional before making any changes to your treatment plan.

1. Lifestyle changes: In addition to taking medication, adopting a heart-healthy lifestyle can help lower cholesterol levels. This can include:
   - Eating a balanced diet rich in fruits, vegetables, whole grains, and lean protein sources
   - Limiting saturated and trans fats, cholesterol, and added sugars
   - Engaging in regular physical activity
   - Maintaining a healthy weight
   - Not smoking or using tobacco products
   - Limiting alcohol consumption
2. Adjusting your statin therapy: Your doctor might consider adjusting your statin dosage or switching to a different statin medication to find the most effective option for you.
3. Combination therapy: In some cases, healthcare providers may recommend combining a statin with other cholesterol-lowering medications, such as ezetimibe, bile acid sequestrants, or PCSK9 inhibitors. These can work together to help lower cholesterol levels more effectively.
4. Investigating underlying causes: High cholesterol can sometimes be caused by other health conditions or medications. Your healthcare provider may want to explore these possibilities and treat any underlying issues.

Remember, it's essential to consult with your healthcare provider before making any changes to your treatment plan, as they can provide personalized advice based on your medical history and current health.

***

**What medications can reduce cholesterol other than statins?**

***

I am not a doctor, but I can provide you with some general information on medications that can reduce cholesterol other than statins. It's important to consult with a healthcare professional for personalized advice and recommendations.

1. Bile acid sequestrants: These medications bind to bile acids in the intestine, which are then eliminated from the body. This prompts the liver to produce more bile acids from cholesterol, leading to a reduction in cholesterol levels. Examples include cholestyramine, colestipol, and colesevelam.
2. Fibrates: These drugs work by reducing triglyceride levels and increasing high-density lipoprotein (HDL) cholesterol levels. Examples include gemfibrozil, fenofibrate, and bezafibrate.
3. Cholesterol absorption inhibitors: This class of medications reduces cholesterol absorption in the small intestine, which can help lower LDL cholesterol levels. An example of this is ezetimibe.
4. Nicotinic acid (Niacin): Niacin, a form of vitamin B3, can help reduce LDL cholesterol and triglyceride levels while raising HDL cholesterol levels. However, it may cause side effects like flushing and itching, and high doses can potentially lead to liver toxicity.
5. PCSK9 inhibitors: These are a newer class of injectable medications that can help lower LDL cholesterol by increasing the liver's ability to remove it from the blood. Examples include alirocumab and evolocumab.
6. Omega-3 fatty acids: Prescription-strength omega-3 fatty acid supplements (e.g., icosapent ethyl) can help lower triglyceride levels, but they do not have a significant impact on LDL cholesterol.

Lifestyle changes, such as adopting a healthy diet, exercising regularly, quitting smoking, and losing weight if necessary, can also help improve cholesterol levels. It is important to consult with a healthcare professional before starting any new medication or making significant lifestyle changes.

***

I am not a doctor, but I can offer some information on cholesterol-lowering medications that are not statins. It's important to talk to your healthcare provider before starting any new medications. Some alternatives to statins include:

1. Bile acid sequestrants: These drugs work by binding to bile acids in the intestines, which are needed for digestion. The body then uses cholesterol to produce more bile acids, leading to a decrease in cholesterol levels. Examples include cholestyramine, colesevelam, and colestipol.
2. Fibrates: These medications lower triglyceride levels and can raise HDL ("good") cholesterol. They work by reducing the production of triglycerides in the liver. Examples include fenofibrate, gemfibrozil, and bezafibrate.
3. Niacin (vitamin B3): Niacin can help lower LDL ("bad") cholesterol and triglycerides and raise HDL cholesterol. However, it can cause side effects such as flushing and gastrointestinal issues. It is important to use niacin under the supervision of a healthcare provider.
4. Cholesterol absorption inhibitors: These drugs block the absorption of cholesterol in the small intestine, which can help lower LDL cholesterol levels. The most common example is ezetimibe.
5. PCSK9 inhibitors: These are a newer class of injectable medications that help lower LDL cholesterol levels by increasing the liver's ability to remove LDL cholesterol from the blood. Examples include alirocumab and evolocumab.
6. Omega-3 fatty acid supplements: Some research suggests that omega-3 fatty acids, found in fish oil, can help lower triglyceride levels. These supplements are available over-the-counter, but it's essential to discuss their use with a healthcare provider.

Please consult with your healthcare provider to determine the most appropriate treatment plan for your individual needs, as they will be able to consider your medical history, risk factors, and any potential drug interactions.

***

I am not a doctor, but I can provide some general information about cholesterol-lowering medications other than statins. You should always consult with a healthcare professional before starting any new medication.

Some non-statin cholesterol-lowering medications include:

1. Bile acid sequestrants: These drugs bind to bile acids in the intestine, preventing their reabsorption and promoting their excretion. This leads to reduced cholesterol levels in the blood. Examples include cholestyramine, colestipol, and colesevelam.
2. Cholesterol absorption inhibitors: Ezetimibe is the most common drug in this class. It works by reducing the absorption of dietary cholesterol in the small intestine, which results in decreased blood cholesterol levels.
3. Fibrates: These drugs help lower triglyceride levels and can modestly increase HDL ("good") cholesterol. Examples include gemfibrozil, fenofibrate, and clofibrate. They may be used in combination with statins in certain cases, but this must be done carefully due to an increased risk of side effects.
4. Niacin (Vitamin B3): Niacin, also known as nicotinic acid, can help lower LDL ("bad") cholesterol and triglycerides while raising HDL cholesterol. However, it may cause side effects like flushing, itching, and stomach upset. High doses of niacin should be taken under medical supervision.
5. PCSK9 inhibitors: These are a newer class of injectable medications that can significantly lower LDL cholesterol. Examples include evolocumab and alirocumab. They work by inhibiting the action of a protein called PCSK9, which results in increased clearance of LDL cholesterol from the blood.
6. Omega-3 fatty acids: Prescription-strength omega-3 fatty acid supplements, such as icosapent ethyl, can help lower triglyceride levels. They are typically derived from fish oil and can be used in combination with other cholesterol-lowering medications.

Remember, it is important to discuss your specific situation with a healthcare professional to determine the best course of action for managing your cholesterol levels.

***

**What is ezetimibe?**

***

Ezetimibe is a medication used to help lower blood cholesterol levels. It belongs to a class of drugs known as cholesterol absorption inhibitors. Ezetimibe works by blocking the absorption of cholesterol in the small intestine, which in turn reduces the amount of cholesterol that enters the bloodstream. This helps decrease low-density lipoprotein (LDL) cholesterol levels, often referred to as "bad" cholesterol, which can contribute to the development of atherosclerosis (plaque buildup in the arteries) and increase the risk of heart disease and stroke.

Ezetimibe is commonly prescribed either alone or in combination with statins, another class of cholesterol-lowering medications. Statins work by inhibiting the enzyme HMG-CoA reductase, which plays a crucial role in the production of cholesterol in the liver. The combination of ezetimibe and a statin can be more effective in lowering cholesterol levels than either drug alone.

As with any medication, there may be side effects associated with ezetimibe. Some common side effects include headache, diarrhea, and muscle pain. It's important to discuss any concerns with your healthcare provider and follow their recommendations for proper use and monitoring.

***

Ezetimibe is a medication used to help lower blood cholesterol levels. It is classified as a cholesterol absorption inhibitor, which means that it works by blocking the absorption of cholesterol from the intestines. By doing so, ezetimibe can help to reduce the total amount of cholesterol and low-density lipoprotein (LDL) cholesterol, also known as "bad" cholesterol, in the blood.

Ezetimibe is often prescribed in combination with statins, another class of cholesterol-lowering drugs, for patients who need additional help in managing their cholesterol levels or who cannot tolerate statins due to side effects. The combination of ezetimibe and a statin has been shown to be more effective at lowering LDL cholesterol than either drug alone.

Ezetimibe is usually taken as a tablet, once daily. It is important to follow your healthcare provider's instructions regarding dosage and any dietary or lifestyle changes that may be necessary while taking this medication.

***

Ezetimibe is a prescription medication used to help lower cholesterol levels in the blood. It belongs to a class of drugs called cholesterol absorption inhibitors, which work by reducing the absorption of cholesterol from the intestines into the bloodstream. This ultimately leads to a decrease in the amount of total cholesterol, low-density lipoprotein (LDL) cholesterol, and triglycerides in the blood, while modestly increasing high-density lipoprotein (HDL) cholesterol levels.

Ezetimibe is often used in combination with statins, another class of cholesterol-lowering medications, to enhance their effectiveness in reducing cholesterol levels. It may also be used alone in patients who cannot tolerate statins or when statins are contraindicated. The drug is typically prescribed as part of a comprehensive treatment plan that includes lifestyle changes such as a healthy diet, exercise, and weight management to help control cholesterol levels and reduce the risk of heart disease and stroke.

Common side effects of ezetimibe include headache, diarrhea, muscle pain, and upper respiratory infection, although many people taking the medication do not experience significant side effects. As with any medication, it is important to follow the prescribing doctor's instructions and report any unusual or severe side effects.

***

**What are Repatha and Praluent?**

***

Repatha (evolocumab) and Praluent (alirocumab) are two brand-name medications classified as PCSK9 inhibitors. These drugs are used to lower low-density lipoprotein cholesterol (LDL-C) levels in the blood, often referred to as "bad" cholesterol. Elevated LDL-C levels are associated with an increased risk of cardiovascular disease, including heart attacks and strokes.

Both Repatha and Praluent work by inhibiting the action of proprotein convertase subtilisin/kexin type 9 (PCSK9), a protein that regulates cholesterol levels. By inhibiting PCSK9, these drugs increase the number of LDL receptors on liver cells, which, in turn, helps remove more LDL cholesterol from the blood, resulting in lower LDL-C levels.

Repatha and Praluent are typically prescribed to patients who have not achieved adequate cholesterol control through diet and statin therapy alone or who have certain genetic conditions, such as familial hypercholesterolemia, that lead to very high cholesterol levels. These medications are administered as subcutaneous injections, usually once or twice a month, depending on the prescribed dosage.

It is important to note that while Repatha and Praluent can significantly reduce LDL-C levels, they are not intended as a replacement for a heart-healthy lifestyle, which includes a balanced diet, regular exercise, and maintaining a healthy weight.

***

Repatha (evolocumab) and Praluent (alirocumab) are two medications that belong to a class of drugs called proprotein convertase subtilisin/kexin type 9 (PCSK9) inhibitors. These drugs are designed to lower low-density lipoprotein (LDL) cholesterol, also known as "bad" cholesterol, in the blood. High levels of LDL cholesterol can lead to atherosclerosis, a condition in which fatty deposits build up in the arteries, increasing the risk of heart attack, stroke, and other cardiovascular events.

Both Repatha and Praluent work by blocking the action of PCSK9, a protein that reduces the liver's ability to remove LDL cholesterol from the bloodstream. By inhibiting PCSK9, these drugs increase the liver's capacity to clear LDL cholesterol, thereby lowering overall LDL cholesterol levels in the blood.

Repatha and Praluent are typically prescribed to patients who have not been able to achieve desired cholesterol levels through diet, exercise, and other cholesterol-lowering medications such as statins. They may also be prescribed for individuals with familial hypercholesterolemia, a genetic condition that causes extremely high cholesterol levels and increases the risk of early-onset heart disease. These drugs are administered through subcutaneous injections, usually once or twice a month.

***

Repatha (evolocumab) and Praluent (alirocumab) are two medications that belong to a class of drugs called proprotein convertase subtilisin/kexin type 9 (PCSK9) inhibitors. They are both monoclonal antibodies that work by inhibiting the action of PCSK9, a protein that plays a significant role in regulating the levels of low-density lipoprotein cholesterol (LDL-C) in the blood.

High levels of LDL-C, often referred to as "bad cholesterol," are associated with an increased risk of developing atherosclerosis and cardiovascular disease. By inhibiting the function of PCSK9, Repatha and Praluent help to lower LDL-C levels, which may reduce the risk of heart attacks, strokes, and other cardiovascular events.

Both Repatha and Praluent are approved for use in certain patients with high cholesterol levels who have not been able to lower their LDL-C enough through diet and statin therapy, or in patients with familial hypercholesterolemia (FH), a genetic condition characterized by extremely high cholesterol levels.

These drugs are administered by subcutaneous injection, usually every two to four weeks, depending on the specific drug and dosage. While they can be effective in reducing LDL-C levels, they may also cause some side effects, such as injection site reactions, cold or flu-like symptoms, and allergic reactions. It's important to discuss the potential benefits and risks of these medications with a healthcare provider.

***

**What is inclisiran?**

***

Inclisiran is a small interfering RNA (siRNA) molecule that has been developed as a treatment for high cholesterol, specifically targeting low-density lipoprotein cholesterol (LDL-C), also known as "bad" cholesterol. High levels of LDL-C are associated with an increased risk of cardiovascular diseases, such as heart attacks and strokes.

Inclisiran works by inhibiting the production of an enzyme called proprotein convertase subtilisin/kexin type 9 (PCSK9). PCSK9 plays a role in regulating cholesterol levels in the blood by promoting the degradation of LDL receptors on the surface of liver cells. When these receptors are reduced, the liver is less efficient at removing LDL-C from the blood, leading to higher cholesterol levels. By inhibiting PCSK9, inclisiran increases the number of LDL receptors on liver cells, which in turn helps to lower LDL-C levels in the blood.

Inclisiran has been studied in various clinical trials and has demonstrated promising results in reducing LDL-C levels, with a relatively low incidence of side effects. It is administered as a subcutaneous injection and is typically given every six months. Inclisiran may be prescribed as an add-on therapy to other cholesterol-lowering medications, such as statins, for patients who require further LDL-C reduction or who cannot tolerate other treatments.

***

Inclisiran is a novel cholesterol-lowering medication that belongs to a class of drugs called small interfering RNA (siRNA) therapeutics. It is designed to lower low-density lipoprotein cholesterol (LDL-C), commonly known as "bad cholesterol," in people with high cholesterol levels, particularly those who do not achieve optimal LDL-C levels with other treatments, such as statins.

Inclisiran works by inhibiting the synthesis of proprotein convertase subtilisin/kexin type 9 (PCSK9), a protein that plays a crucial role in regulating cholesterol levels in the bloodstream. By blocking PCSK9 production, inclisiran allows the liver to remove more LDL-C from the blood, thereby reducing the overall cholesterol levels.

Inclisiran has been shown to be effective in reducing LDL-C levels in clinical trials and has been approved for use in various countries. It is typically administered as a subcutaneous injection, often at six-month intervals. As with any medication, it is essential to consult a healthcare professional before starting inclisiran to ensure it is appropriate for your specific situation and to discuss potential side effects and interactions with other medications you may be taking.

***

Inclisiran is a novel small interfering RNA (siRNA) molecule that functions as a cholesterol-lowering drug. It was developed for the treatment of hypercholesterolemia, a condition characterized by high levels of cholesterol in the blood. Inclisiran works by targeting and inhibiting the production of an enzyme called proprotein convertase subtilisin/kexin type 9 (PCSK9).

PCSK9 is a protein that plays a role in regulating the amount of low-density lipoprotein cholesterol (LDL-C) in the bloodstream. Elevated levels of LDL-C, commonly known as "bad cholesterol," are associated with an increased risk of cardiovascular diseases such as heart attacks and strokes. By inhibiting PCSK9, inclisiran allows the liver to more effectively clear LDL-C from the bloodstream, ultimately lowering cholesterol levels.

Inclisiran is administered as a subcutaneous injection and is usually given in conjunction with other cholesterol-lowering treatments, such as statins or ezetimibe, when these medications alone do not adequately control cholesterol levels. The drug has shown promising results in clinical trials, and it has the potential to be a valuable addition to the arsenal of therapies for managing high cholesterol and reducing the risk of cardiovascular events.

***

**What are the side effects of Repatha and Praluent?**

***

Repatha (evolocumab) and Praluent (alirocumab) are two medications that belong to a class of drugs called PCSK9 inhibitors. They are used to lower LDL cholesterol (the "bad" cholesterol) levels in certain patients who have not been able to lower their cholesterol through diet and other cholesterol-lowering medications. Both drugs work by blocking the action of a protein called PCSK9, which helps the liver to remove LDL cholesterol from the bloodstream.

While these medications can be effective in lowering cholesterol levels, they can also cause some side effects. It's essential to discuss these with your healthcare provider before starting any new medication.

Common side effects of Repatha and Praluent include:

1. Injection site reactions: Redness, itching, swelling, or pain at the site of injection can occur with both medications, as they are administered via subcutaneous injection.
2. Flu-like symptoms: Patients may experience symptoms such as fever, chills, body aches, or fatigue.
3. Nasopharyngitis: Inflammation of the nasal passages and the back of the throat may cause symptoms like a stuffy or runny nose, sore throat, or sneezing.
4. Allergic reactions: Both drugs have the potential to cause allergic reactions, which may include rash, hives, difficulty breathing, or swelling of the face, lips, tongue, or throat. Seek immediate medical attention if you experience any signs of an allergic reaction.

Less common side effects may include:

1. Muscle pain or weakness: Some patients may experience muscle-related side effects, such as pain, stiffness, or weakness. In rare cases, a serious muscle condition called rhabdomyolysis may occur, which can lead to kidney damage.
2. Neurological symptoms: Some patients may experience headache, dizziness, or confusion while on these medications.

It's important to note that this list is not exhaustive, and other side effects may occur. If you experience any new or concerning symptoms while taking Repatha or Praluent, contact your healthcare provider promptly. They will be able to guide you on the best course of action and determine if the medication is right for you.

***

Repatha (evolocumab) and Praluent (alirocumab) are monoclonal antibodies that belong to a class of medications called PCSK9 inhibitors. They are used to lower low-density lipoprotein cholesterol (LDL-C, or "bad" cholesterol) in adults with heterozygous familial hypercholesterolemia, homozygous familial hypercholesterolemia, or clinical atherosclerotic cardiovascular disease when other treatments such as diet and statins are not enough.

As with any medication, both Repatha and Praluent may cause side effects. The most common side effects for these two drugs are generally mild to moderate and may include:

1. Injection site reactions: Redness, swelling, pain, or itching at the site of injection are common side effects. These symptoms usually resolve on their own within a few days.
2. Nasopharyngitis: Common cold-like symptoms, such as a runny or stuffy nose, sneezing, and sore throat, may occur.
3. Upper respiratory tract infection: Symptoms may include cough, sore throat, and fever.
4. Influenza: Flu-like symptoms, including fever, chills, body aches, and fatigue, may occur.
5. Back pain: Some patients may experience back pain while using these medications.
6. Allergic reactions: Although rare, serious allergic reactions can occur, including rash, difficulty breathing, and swelling of the face, lips, tongue, or throat. If you experience any of these symptoms, contact your healthcare provider immediately or seek emergency medical attention.

It is important to remember that this is not an exhaustive list of side effects. Some people may experience other side effects or have individual reactions to these medications. Always consult your healthcare provider if you have any concerns or questions about the side effects of your medications.

***

Repatha (evolocumab) and Praluent (alirocumab) are two monoclonal antibody drugs that belong to the class of medications known as PCSK9 inhibitors. They are used to lower LDL cholesterol levels in people with high cholesterol, particularly those who have not responded adequately to statins or other cholesterol-lowering therapies. Although these drugs can be effective, they may cause side effects in some individuals.

Common side effects of Repatha and Praluent include:

1. Injection site reactions: Redness, pain, itching, or swelling at the injection site are common with both drugs.
2. Nasopharyngitis: Patients may experience a runny or stuffy nose, sneezing, or sore throat.
3. Upper respiratory tract infections: Cough, congestion, or sinus infections may occur.
4. Influenza: Flu-like symptoms such as fever, chills, body aches, and fatigue are possible.
5. Back pain: Some patients may experience pain or discomfort in the back.
6. Allergic reactions: Although rare, some people may have allergic reactions to these medications, including rash, itching, swelling of the face or throat, difficulty breathing, or severe dizziness.

It is important to note that not everyone will experience these side effects, and many people tolerate Repatha and Praluent well. If you experience any side effects or have concerns about your medication, consult your healthcare provider for guidance. They can help determine if these medications are appropriate for your situation or if alternative treatment options should be considered.

***

**Should I take aspirin to prevent heart disease?**

***

I am not a doctor, but I can provide you with general information about aspirin and heart disease prevention. It is important to consult with a healthcare professional before starting any new medication or treatment.

Aspirin has been used for many years as a blood thinner to help prevent heart attacks and strokes in some individuals. It works by inhibiting the formation of blood clots, which can block blood flow to the heart or brain.

There are guidelines that recommend the use of low-dose aspirin for certain high-risk individuals, such as those with a history of heart attack or stroke, or those with certain risk factors for cardiovascular disease. However, the decision to take aspirin for heart disease prevention is not a one-size-fits-all approach and should be individualized based on your unique medical history and risk factors.

Some potential downsides of taking aspirin include an increased risk of bleeding, particularly in the gastrointestinal tract, and a possible increased risk of hemorrhagic stroke.

In summary, it is crucial to discuss your personal risk factors and medical history with a healthcare professional before deciding to take aspirin for heart disease prevention. They will be able to provide you with the best advice based on your individual circumstances.

***

I am not a doctor, but I can provide you with some general information on aspirin and heart disease prevention. Aspirin is a blood thinner and may help prevent blood clots that can lead to heart attacks and strokes. Some studies suggest that taking low-dose aspirin daily can reduce the risk of heart attacks, particularly in people with a history of heart disease or certain risk factors.

However, aspirin is not without risks. Regular use of aspirin can increase the risk of gastrointestinal bleeding and may cause other side effects. The decision to use aspirin for heart disease prevention should be made on an individual basis, taking into account your personal risk factors, medical history, and the potential benefits and risks.

It is important to consult with a healthcare professional before starting or stopping any medication, including aspirin. They will be able to provide personalized advice and recommendations based on your specific circumstances.

***

I am not a doctor, but I can provide you with some general information about aspirin and its potential role in heart disease prevention. Aspirin is sometimes recommended for certain individuals to help prevent heart attacks and strokes, particularly in those who have already experienced a cardiovascular event or are at high risk.

However, whether or not you should take aspirin for this purpose depends on your personal health history and risk factors, as well as potential side effects and drug interactions. It is crucial to consult with a healthcare professional before starting aspirin therapy, as they can help you determine if the benefits outweigh the risks in your specific case.

***

**My cholesterol panel shows triglycerides 400 mg/dL. How should I interpret this?**

***

I am not a doctor, but I can provide you with some general information about triglyceride levels. If you have any concerns, please consult a healthcare professional for personalized advice.

Triglycerides are a type of fat (lipid) found in your blood. They are used by the body as a source of energy. High triglyceride levels, also known as hypertriglyceridemia, can be a risk factor for heart disease and other health problems.

According to the American Heart Association, triglyceride levels are typically classified as follows:

- Normal: Less than 150 mg/dL
- Borderline high: 150 to 199 mg/dL
- High: 200 to 499 mg/dL
- Very high: 500 mg/dL or higher

With a triglyceride level of 400 mg/dL, your results would be considered high. High triglyceride levels can be caused by various factors, including obesity, lack of physical activity, smoking, excessive alcohol consumption, a high-carbohydrate diet, certain medications, and some medical conditions.

It is important to consult with a healthcare professional to discuss your results and determine the appropriate course of action. They may recommend lifestyle changes or medications to help lower your triglyceride levels and reduce your risk of health complications.

***

I am not a doctor, but I can provide you with some general information about triglyceride levels based on common guidelines. It's important to consult a healthcare professional for personalized advice.

A triglyceride level of 400 mg/dL is considered very high. Triglycerides are a type of fat found in the blood, and high levels can be a risk factor for heart disease and other health problems. Here's a general classification of triglyceride levels:

- Normal: Less than 150 mg/dL
- Borderline high: 150-199 mg/dL
- High: 200-499 mg/dL
- Very high: 500 mg/dL or higher

There are various factors that can contribute to elevated triglyceride levels, including obesity, physical inactivity, smoking, excess alcohol consumption, and a diet high in carbohydrates and unhealthy fats. Certain medical conditions and medications can also raise triglyceride levels.

To manage high triglycerides, healthcare professionals may recommend lifestyle changes, such as:

1. Adopting a healthier diet: Consume more fruits, vegetables, whole grains, lean proteins, and healthy fats (such as those found in fish, nuts, and olive oil). Limit saturated and trans fats, added sugars, and refined carbohydrates.
2. Engaging in regular physical activity: Aim for at least 150 minutes of moderate-intensity aerobic exercise or 75 minutes of vigorous-intensity aerobic exercise per week, along with muscle-strengthening activities on 2 or more days a week.
3. Losing weight if overweight or maintaining a healthy weight.
4. Quitting smoking.
5. Limiting alcohol consumption: Men should consume no more than two drinks per day, and women no more than one drink per day.

In some cases, medication may also be prescribed to help lower triglyceride levels. It's crucial to consult with a healthcare professional to determine the best course of action for your specific situation.

***

I am not a doctor, but I can provide some general information about cholesterol and triglyceride levels. It's important to consult with a healthcare professional for personalized advice.

Triglycerides are a type of fat found in the blood, and they play a role in energy storage and transport. A triglyceride level of 400 mg/dL is considered high according to the American Heart Association's guidelines:

- Normal: less than 150 mg/dL
- Borderline high: 150 to 199 mg/dL
- High: 200 to 499 mg/dL
- Very high: 500 mg/dL or higher

High triglyceride levels can increase your risk of developing cardiovascular disease, heart attack, stroke, and other health issues. There are several factors that can contribute to elevated triglyceride levels, including obesity, inactivity, excessive alcohol consumption, and a diet high in saturated fats and simple sugars.

To manage high triglyceride levels, it is usually recommended to make lifestyle changes, such as:

1. Adopting a heart-healthy diet: Emphasize fruits, vegetables, whole grains, lean proteins, and healthy fats (e.g., from fish, nuts, and olive oil).
2. Exercising regularly: Aim for at least 150 minutes of moderate-intensity aerobic activity or 75 minutes of vigorous aerobic activity per week.
3. Losing weight: If you're overweight, losing even a small amount of weight can help lower triglyceride levels.
4. Limiting alcohol: Alcohol can raise triglyceride levels, so it's important to moderate your intake or abstain entirely.
5. Quitting smoking: Smoking can negatively impact your cholesterol and triglyceride levels.

It is crucial to consult with a healthcare professional to discuss your test results, assess your risk factors, and develop a personalized plan to manage your triglyceride levels. In some cases, medication may be prescribed in addition to lifestyle changes.

***

**My LDL is 200 mg/dL. How should I interpret this?**

***

I am not a doctor, but I can provide you with some general information about LDL cholesterol levels.

LDL (Low-Density Lipoprotein) cholesterol is often referred to as "bad" cholesterol because high levels can contribute to the development of plaque in your arteries, increasing your risk for heart disease and stroke. The American Heart Association (AHA) and other health organizations have established guidelines for interpreting LDL cholesterol levels:

- Optimal: Less than 100 mg/dL
- Near optimal/above optimal: 100-129 mg/dL
- Borderline high: 130-159 mg/dL
- High: 160-189 mg/dL
- Very high: 190 mg/dL and above

With an LDL level of 200 mg/dL, your level would be considered very high according to these guidelines. It is important to discuss these results with your healthcare provider, as they can provide personalized recommendations based on your overall health and risk factors. They may suggest lifestyle changes, such as adopting a healthier diet, exercising regularly, and quitting smoking, as well as medications to help lower your cholesterol levels.

Remember to always consult a healthcare professional for personalized advice and guidance.

***

I am not a doctor, but I can provide some general information that may help you understand your LDL cholesterol level. LDL (low-density lipoprotein) cholesterol is often referred to as "bad cholesterol" because high levels of LDL can contribute to plaque buildup in your arteries, increasing the risk of heart disease and stroke.

A LDL level of 200 mg/dL is considered to be quite high. The American Heart Association provides the following general guidelines for interpreting LDL cholesterol levels for adults:

- Optimal: Less than 100 mg/dL
- Near optimal/above optimal: 100-129 mg/dL
- Borderline high: 130-159 mg/dL
- High: 160-189 mg/dL
- Very high: 190 mg/dL and above

It's important to remember that these are general guidelines and that individual factors such as age, sex, family history, and presence of other medical conditions can influence the interpretation of your LDL level. It's best to discuss your cholesterol levels with a healthcare professional, who can provide personalized recommendations based on your overall health, risk factors, and lifestyle.

To lower your LDL cholesterol, your doctor may suggest lifestyle changes such as eating a heart-healthy diet, increasing physical activity, maintaining a healthy weight, and quitting smoking. In some cases, medication may also be prescribed to help lower cholesterol levels.

***

I am not a doctor, but I can provide some general information about LDL cholesterol levels. LDL (low-density lipoprotein) cholesterol is often referred to as "bad" cholesterol because high levels can contribute to the buildup of plaque in your arteries, which can lead to heart disease and stroke.

Based on the American Heart Association and other health organizations' guidelines, here is a general breakdown of LDL cholesterol levels:

- Optimal: Less than 100 mg/dL
- Near optimal: 100-129 mg/dL
- Borderline high: 130-159 mg/dL
- High: 160-189 mg/dL
- Very high: 190 mg/dL and above

With an LDL level of 200 mg/dL, your LDL cholesterol would be considered very high. It is important to consult with a healthcare professional to discuss your individual risk factors, obtain a proper evaluation, and develop a personalized plan to lower your cholesterol if necessary. Lifestyle changes and medications may be recommended to help reduce your LDL levels and improve your overall heart health.

***

**What does a coronary calcium score of 0 mean?**

***

A coronary calcium score of 0 means that no calcified plaque was detected in the coronary arteries. This score is generally considered a good result, as it suggests a low risk of coronary artery disease or future cardiovascular events. However, it does not guarantee that a person is free from heart disease or that they will never experience a heart-related problem. It is important to maintain a healthy lifestyle and consult with a healthcare professional for personalized advice on cardiovascular risk factors and prevention.

***

A coronary calcium score of 0 means that there is no detectable calcification in the coronary arteries. The coronary calcium score, also known as the Agatston score, is a non-invasive test that measures the amount of calcium in the walls of the coronary arteries, which supply blood to the heart muscle. The presence of calcium is an indicator of coronary artery disease (CAD) and atherosclerosis.

A score of 0 suggests a very low risk of developing a heart-related event, such as a heart attack, in the next 5-10 years. However, it's important to note that the absence of calcium does not completely rule out the presence of plaque, as some plaques may be non-calcified. Therefore, it's essential to maintain a heart-healthy lifestyle and follow any recommendations provided by your healthcare provider to minimize the risk of heart disease.

***

A coronary calcium score of 0 means that there is no detectable calcified plaque in the coronary arteries. This test, also known as a coronary artery calcium (CAC) scan, measures the amount of calcified plaque in the arteries that supply blood to the heart. The score is derived from a CT scan and is reported in Agatston units.

A score of 0 is generally considered a favorable result, indicating a low risk of developing coronary artery disease (CAD) in the near future. However, it does not entirely eliminate the risk, as non-calcified plaques or other risk factors may still be present. It's important to maintain a heart-healthy lifestyle and follow recommendations from your healthcare provider to minimize the risk of developing heart disease in the long term.

***

**What does a coronary calcium score of 100 mean?**

***

A coronary calcium score, also known as the Agatston score, is a measurement of the amount of calcium in the walls of the coronary arteries. The score is an indicator of atherosclerosis, which is the buildup of plaque in the arteries that can lead to heart disease. The calcium score is derived from a CT scan of the heart and is given as a number ranging from 0 to several thousand.

A coronary calcium score of 100 means that there is a moderate amount of calcium in the coronary arteries, which may suggest some degree of plaque buildup. This places the individual at a higher risk of heart disease compared to someone with a lower score.

The calcium scores are usually categorized as follows:

1. 0: No identifiable plaque. Very low risk of a coronary event.
2. 1-10: Minimal plaque. Very low risk of a coronary event.
3. 11-100: Mild plaque. Low to moderate risk of a coronary event.
4. 101-400: Moderate plaque. Moderate to high risk of a coronary event.
5. Over 400: Extensive plaque. High risk of a coronary event.

It is important to note that a coronary calcium score is just one of many factors used to assess an individual's overall risk of heart disease. Other risk factors such as age, family history, blood pressure, cholesterol levels, diabetes, and lifestyle habits (e.g., smoking, physical activity, and diet) are also taken into consideration. If you have a coronary calcium score of 100, it is essential to discuss your overall risk and potential lifestyle changes or medical interventions with your healthcare provider.

***

A coronary calcium score, also known as a coronary artery calcium (CAC) score, is a non-invasive test that quantifies the amount of calcium in the walls of the coronary arteries, which supply blood to the heart muscle. This calcium buildup is indicative of plaque formation, a sign of coronary artery disease (CAD).

A coronary calcium score of 100 means that there is a moderate amount of calcium present in the coronary arteries. The scores are usually categorized as follows:

- 0: No detectable plaque or calcium
- 1-10: Minimal plaque burden
- 11-100: Mild plaque burden
- 101-400: Moderate plaque burden
- 400: Extensive plaque burden

A score of 100 typically indicates a mild to moderate risk of developing heart-related issues, such as a heart attack, in the future. However, it's important to note that the coronary calcium score is just one piece of the puzzle when assessing cardiovascular risk. Factors such as age, sex, cholesterol levels, blood pressure, smoking status, and family history should also be taken into consideration when determining an individual's overall risk.

It is important to consult with a healthcare professional to discuss the implications of a coronary calcium score of 100, as well as potential lifestyle changes, medications, or interventions to manage cardiovascular risk.

***

A coronary calcium score of 100 is a measure of the amount of calcification or calcium deposits in the coronary arteries, which supply blood to the heart muscle. The score is derived from a specialized computed tomography (CT) scan called a coronary calcium scan, which noninvasively evaluates the extent of calcification.

The coronary calcium score is an indicator of the risk of developing coronary artery disease (CAD) and having a cardiovascular event, such as a heart attack. A score of 100 is generally considered to be moderate. Here's a breakdown of the scores and their associated risks:

1. Score of 0: No detectable calcium deposits, very low risk of CAD.
2. Score of 1-10: Minimal calcium deposits, low risk of CAD.
3. Score of 11-100: Mild calcium deposits, mild to moderate risk of CAD.
4. Score of 101-400: Moderate calcium deposits, moderate to high risk of CAD.
5. Score of >400: Extensive calcium deposits, high risk of CAD.

If you have a coronary calcium score of 100, it means you have a moderate amount of calcification in your coronary arteries, which may indicate a higher risk of developing heart problems compared to someone with a lower score. It is important to discuss your results with your healthcare provider, who will consider other factors such as your age, sex, family history, and presence of other risk factors to determine your overall risk for CAD and suggest appropriate lifestyle changes or medical interventions to manage your risk.

***

**What does a coronary calcium score of 400 mean?**

***

A coronary calcium score, also known as a coronary artery calcium (CAC) score, is a non-invasive test that quantifies the amount of calcium in the coronary arteries. The presence of calcium is an indication of atherosclerosis or plaque buildup, which can increase the risk of heart disease.

A CAC score of 400 means that a significant amount of calcium has been detected in the coronary arteries, which suggests moderate to severe coronary artery disease. This score is typically categorized as a high risk, and individuals with such a score have an increased likelihood of experiencing a cardiac event, such as a heart attack or stroke.

However, it's important to note that the CAC score is just one part of assessing cardiovascular risk. Other factors, such as age, gender, family history, blood pressure, cholesterol levels, and lifestyle choices, should be considered when determining an individual's overall risk for heart disease.

If you or someone you know has a CAC score of 400, it's important to consult with a healthcare professional to discuss the appropriate steps for managing the risk of heart disease. This may include lifestyle changes, medications, or more aggressive interventions, depending on the individual's overall risk profile.

***

A coronary calcium score, also known as a coronary artery calcium (CAC) score, is a measure of the amount of calcified plaque present in the coronary arteries. The score is derived from a non-invasive imaging test called a coronary CT scan, which uses X-ray technology to visualize the heart's blood vessels.

A coronary calcium score of 400 indicates a significant amount of calcified plaque in the coronary arteries, which is associated with a higher risk of heart disease and coronary events such as heart attacks. In general, scores are interpreted as follows:

1. 0: No identifiable plaque, very low risk of heart disease
2. 1-10: Minimal plaque, low risk of heart disease
3. 11-100: Mild plaque, moderate risk of heart disease
4. 101-400: Moderate plaque, moderately high risk of heart disease
5. 400: Extensive plaque, high risk of heart disease

A score of 400 or higher is considered high and suggests a greater likelihood of a future coronary event. It's important to note that the coronary calcium score is just one factor in assessing cardiovascular risk, and it should be interpreted in the context of other risk factors such as age, sex, cholesterol levels, blood pressure, smoking status, and family history.

If you have a high coronary calcium score, your doctor may recommend lifestyle modifications, such as adopting a heart-healthy diet, exercising regularly, maintaining a healthy weight, and quitting smoking, as well as medications to manage blood pressure, cholesterol, or other risk factors. It's essential to consult with a healthcare professional to determine the best course of action for your specific situation.

***

A coronary calcium score of 400 is a measure of the amount of calcium in the coronary arteries, which are the blood vessels that supply oxygen and nutrients to the heart muscle. This score is derived from a non-invasive imaging test called a coronary artery calcium (CAC) scan, which uses computed tomography (CT) to detect and quantify the amount of calcium in the coronary arteries.

A score of 400 indicates a significant amount of calcification in the coronary arteries. Calcification is a sign of atherosclerosis, which is the build-up of plaque (consisting of fat, cholesterol, calcium, and other substances) in the arterial walls. This build-up can narrow the arteries and reduce blood flow to the heart, increasing the risk of heart attack and other cardiovascular events.

A coronary calcium score is generally categorized into the following risk levels:

1. Zero: No detectable calcium, very low risk of coronary artery disease (CAD)
2. 1-10: Minimal calcium, low risk of CAD
3. 11-100: Mild calcium, moderate risk of CAD
4. 101-400: Moderate calcium, moderately high risk of CAD
5. Above 400: Extensive calcium, high risk of CAD

A score of 400 or higher suggests a high risk of coronary artery disease, and it is important to consult with a healthcare professional for further evaluation and management of cardiovascular risk factors. This may include lifestyle modifications, such as diet, exercise, and smoking cessation, as well as medications to control blood pressure, cholesterol, and other risk factors.

***

**What genetic mutations can cause high cholesterol?**

***

High cholesterol can be caused by various genetic mutations, which can lead to different forms of familial hypercholesterolemia (FH). FH is an inherited disorder characterized by high levels of low-density lipoprotein cholesterol (LDL-C) in the blood, increasing the risk of premature cardiovascular disease. Some of the main genetic mutations associated with high cholesterol include:

1. LDL receptor (LDLR) gene mutations: The most common cause of FH, these mutations affect the LDLR protein's ability to remove LDL-C from the blood, resulting in elevated LDL-C levels. Over 1,700 different LDLR mutations have been identified, and they can cause varying degrees of FH severity.
2. Apolipoprotein B (APOB) gene mutations: APOB mutations affect the apolipoprotein B protein, a component of LDL-C particles that binds to the LDLR for clearance from the blood. A common APOB mutation, known as R3500Q, impairs the interaction between apolipoprotein B and the LDLR, leading to higher LDL-C levels.
3. Proprotein convertase subtilisin/kexin type 9 (PCSK9) gene mutations: PCSK9 is a protein involved in regulating LDLR levels. Some gain-of-function mutations in the PCSK9 gene increase the degradation of LDLR, leading to a reduction in the clearance of LDL-C from the blood and causing high cholesterol levels.
4. Low-density lipoprotein receptor adaptor protein 1 (LDLRAP1) gene mutations: Mutations in the LDLRAP1 gene can cause autosomal recessive hypercholesterolemia. The LDLRAP1 protein plays a role in the internalization and recycling of the LDLR. Mutations in this gene lead to impaired LDLR function and elevated LDL-C levels.
5. Other rare genetic mutations: Some less common mutations, such as those in the APOE, LIPA, and ABCG5/ABCG8 genes, can also lead to high cholesterol levels and increased risk of cardiovascular disease.

It's important to note that not all cases of high cholesterol are due to genetic mutations. Lifestyle factors, such as diet, exercise, and smoking, can also contribute to elevated cholesterol levels. If you are concerned about high cholesterol, it's important to consult a healthcare professional for appropriate evaluation, diagnosis, and treatment.

***

High cholesterol can be caused by a variety of factors, including genetic mutations. Some of the most common genetic mutations that can lead to high cholesterol levels include:

1. Familial hypercholesterolemia (FH): This is the most common genetic cause of high cholesterol. FH is caused by mutations in the LDLR (low-density lipoprotein receptor) gene, APOB (apolipoprotein B) gene, or PCSK9 (proprotein convertase subtilisin/kexin type 9) gene. These mutations lead to a reduced ability of the liver to remove LDL cholesterol (the "bad" cholesterol) from the bloodstream, resulting in significantly elevated LDL cholesterol levels.
2. Familial combined hyperlipidemia (FCHL): FCHL is characterized by elevated levels of total cholesterol, LDL cholesterol, and triglycerides. The genetic basis of FCHL is not well understood, but it is thought to be caused by mutations in multiple genes that interact with each other and with environmental factors.
3. Familial defective apolipoprotein B-100: This condition is caused by mutations in the APOB gene, which encodes for apolipoprotein B-100, a protein that is essential for the proper metabolism of LDL cholesterol. The mutations result in a form of apolipoprotein B-100 that is less effective at binding to the LDL receptor, leading to increased LDL cholesterol levels.
4. Sitosterolemia: This rare genetic disorder is caused by mutations in the ABCG5 and ABCG8 genes, which encode for proteins involved in the absorption and excretion of plant sterols. The mutations lead to increased absorption of dietary plant sterols and cholesterol, resulting in elevated blood cholesterol levels.
5. Polygenic hypercholesterolemia: This refers to high cholesterol levels caused by a combination of multiple genetic variants, each with a small effect on cholesterol metabolism. These variants, when combined with environmental factors like diet and lifestyle, can contribute to high cholesterol levels.

It is important to note that genetic mutations are not the only cause of high cholesterol. Lifestyle factors, such as diet, exercise, smoking, and alcohol consumption, can also significantly impact cholesterol levels.

***

High cholesterol can be caused by a variety of factors, including lifestyle choices, age, and genetic mutations. Several genetic mutations can contribute to high cholesterol levels, with some being more prevalent than others. Some of the most well-known genetic mutations associated with high cholesterol include:

1. Familial Hypercholesterolemia (FH): FH is the most common genetic cause of high cholesterol. It is an autosomal dominant disorder caused by mutations in the LDLR (low-density lipoprotein receptor), APOB (apolipoprotein B), or PCSK9 (proprotein convertase subtilisin/kexin type 9) genes. These mutations impair the ability of the body to clear LDL cholesterol (the "bad" cholesterol) from the bloodstream, leading to elevated levels of LDL and increased risk of cardiovascular diseases.
2. Familial Combined Hyperlipidemia (FCHL): FCHL is a common genetic disorder characterized by elevated levels of total cholesterol, LDL cholesterol, and triglycerides. The genetic causes of FCHL are not completely understood, but it is believed to be polygenic, involving multiple genes. The USF1 (upstream transcription factor 1) gene has been implicated in some cases of FCHL.
3. Familial Dysbetalipoproteinemia (Type III Hyperlipoproteinemia): This genetic disorder is caused by mutations in the APOE (apolipoprotein E) gene. The mutations lead to impaired clearance of chylomicron and VLDL (very low-density lipoprotein) remnants, causing high levels of cholesterol and triglycerides.
4. Sitosterolemia: This rare genetic disorder is caused by mutations in either the ABCG5 (ATP-binding cassette transporter G5) or ABCG8 (ATP-binding cassette transporter G8) genes. These genes are involved in the absorption and excretion of plant sterols, and their mutations lead to high levels of both cholesterol and plant sterols in the blood.

It is essential to consult with a healthcare professional if you suspect that you or a family member may have a genetic disorder related to high cholesterol. Early diagnosis and appropriate treatment can help manage cholesterol levels and reduce the risk of cardiovascular diseases.

**Plain Language (Zero-Shot)**

**How can I prevent heart disease?**

**Answer this question using plain and easy-to-understand language in your response.**

***

To prevent heart disease, follow these simple steps:

Eat healthy: Choose more fruits, vegetables, whole grains, lean meats, and low-fat dairy. Cut down on unhealthy fats, salt, and added sugars.

Be active: Aim for at least 150 minutes of moderate exercise or 75 minutes of intense exercise per week. This could be walking, swimming, cycling, or any activity you enjoy.

Don't smoke: If you smoke, quitting is the best thing you can do for your heart. Ask your doctor for help to quit.

Limit alcohol: Stick to one drink a day for women and up to two drinks a day for men. Drinking less or not at all is better for your heart.

Maintain a healthy weight: If you're overweight, losing even a small amount of weight can improve heart health. Aim for a body mass index (BMI) between 18.5 and 24.9.

Manage stress: Find healthy ways to cope with stress, like meditation, yoga, or spending time with loved ones.

Control blood pressure: Check your blood pressure regularly and follow your doctor's advice to keep it within a healthy range.

Manage diabetes: If you have diabetes, work with your doctor to control blood sugar levels, which can lower your risk of heart disease.

Get enough sleep: Aim for 7-9 hours of quality sleep per night. Good sleep helps your body repair and maintain a healthy heart.

Regular check-ups: Visit your doctor for regular check-ups and follow their advice to keep your heart healthy.

***

To prevent heart disease, follow these simple steps:

Eat healthy: Choose a diet rich in fruits, vegetables, whole grains, lean proteins, and healthy fats. Avoid processed foods, sugary drinks, and too much salt.

Exercise regularly: Aim for at least 150 minutes of moderate exercise or 75 minutes of vigorous exercise per week. Mix in activities like walking, swimming, or dancing.

Maintain a healthy weight: Find a balance between the calories you eat and the calories you burn through physical activity.

Don't smoke: If you smoke, quit. And avoid secondhand smoke as much as possible.

Limit alcohol intake: Stick to no more than one drink per day for women and two drinks per day for men.

Manage stress: Practice relaxation techniques, like deep breathing, meditation, or yoga, to help cope with stress.

Keep blood pressure in check: Get regular checkups and follow your doctor's advice to control high blood pressure.

Control cholesterol: Eat a low-fat, low-cholesterol diet and get regular checkups to monitor your cholesterol levels.

Manage diabetes: If you have diabetes, work with your healthcare team to keep your blood sugar levels in check.

Get regular checkups: Visit your doctor regularly for preventive care and early detection of potential problems.

***

To prevent heart disease, follow these simple steps:

Eat healthy: Choose a diet rich in fruits, vegetables, whole grains, lean proteins, and healthy fats. Avoid processed foods, excess salt, and trans fats.

Exercise regularly: Aim for at least 150 minutes of moderate exercise or 75 minutes of vigorous exercise each week. Mix in strength training and flexibility exercises too.

Maintain a healthy weight: Reach and keep a weight that's right for your height and age. This can lower your risk of heart disease.

Don't smoke: If you smoke, quit. Smoking increases your risk of heart disease. Avoid exposure to secondhand smoke too.

Limit alcohol: Drink alcohol in moderation, if at all. Stick to no more than one drink per day for women and two drinks per day for men.

Manage stress: Find healthy ways to cope with stress, like exercise, meditation, or talking to someone you trust.

Control blood pressure: Regularly check your blood pressure and take steps to keep it in a healthy range. This can help prevent damage to your heart.

Monitor cholesterol: Keep your cholesterol levels in check through a healthy diet and regular checkups.

Manage diabetes: If you have diabetes, work with your healthcare team to manage your blood sugar levels.

Get regular checkups: Visit your doctor regularly to stay on top of your heart health and address any potential issues early.

***

**What is the best diet for the heart?**

**Answer this question using plain and easy-to-understand language in your response.**

***

The best diet for a healthy heart is one that includes a variety of whole, unprocessed foods. Focus on eating plenty of fruits, vegetables, whole grains, lean proteins, and healthy fats. Here's a simple guide:

1. Fruits & veggies: Eat a colorful mix of fruits and vegetables, as they provide vitamins, minerals, and antioxidants to keep your heart healthy.
2. Whole grains: Choose whole grains like brown rice, quinoa, whole wheat bread, and oats instead of refined grains to get more fiber and nutrients.
3. Lean proteins: Include lean proteins like fish, skinless chicken, turkey, beans, and legumes in your diet, as they provide the necessary nutrients without too much unhealthy fat.
4. Healthy fats: Consume healthy fats from sources like olive oil, nuts, seeds, and avocados, which can help lower bad cholesterol.
5. Low-fat dairy: Opt for low-fat or non-fat dairy products to reduce saturated fat intake.
6. Limit salt: Keep your salt intake low to help control blood pressure.
7. Avoid processed foods: Stay away from processed and sugary foods, as they can contribute to weight gain and heart problems.

Remember to stay active, maintain a healthy weight, and drink plenty of water for overall heart health. Always talk to your doctor before making significant changes to your diet.

***

The best diet for a healthy heart is one that is rich in fruits, vegetables, whole grains, lean proteins, and healthy fats. Here's a simple explanation of what to include in your diet:

1. Fruits and vegetables: Try to eat a variety of colorful fruits and vegetables, as they provide essential vitamins, minerals, and fiber that help keep your heart healthy.

2. Whole grains: Choose whole grain foods like brown rice, whole wheat bread, and quinoa. They have more fiber and nutrients than refined grains, which is good for your heart.

3. Lean proteins: Include sources of lean protein such as fish, chicken, turkey, beans, and low-fat dairy products. These help build and repair body tissues without adding unhealthy fats.

4. Healthy fats: Eat foods with healthy fats like avocados, nuts, seeds, and olive oil. These fats can help lower bad cholesterol and are good for your heart.

5. Limit salt, sugar, and processed foods: Eating too much salt, sugar, and processed foods can increase your risk of heart problems. Try to limit these in your diet.

Remember, a balanced diet and regular exercise are key to keeping your heart healthy. Talk to your doctor or a dietitian for personalized advice.

***

The best diet for a healthy heart is one that includes a variety of whole, unprocessed foods. Focus on eating fruits, vegetables, whole grains, lean proteins, and healthy fats. Try to avoid too much salt, sugar, and unhealthy fats. Some key points for a heart-healthy diet are:

1. Eat plenty of colorful fruits and vegetables.

2. Choose whole grains like brown rice, quinoa, and whole wheat bread.

3. Pick lean proteins like fish, poultry, beans, and legumes.

4. Include healthy fats from sources like nuts, seeds, avocados, and olive oil.

5. Limit salty and sugary foods, as well as saturated and trans fats.

Remember, it's also important to watch your portion sizes and stay active for overall heart health.

***

**What is the best diet for high blood pressure and high cholesterol?**

**Answer this question using plain and easy-to-understand language in your response.**

***

A good diet for high blood pressure and high cholesterol includes:

1. Fruits and vegetables: Eat lots of them as they are low in calories and high in vitamins, minerals, and fiber.

2. Whole grains: Choose whole grains like brown rice, whole wheat bread, and quinoa over refined grains like white rice and white bread.

3. Low-fat dairy: Pick low-fat or fat-free milk, yogurt, and cheese to reduce saturated fat intake.

4. Lean protein: Opt for lean sources of protein like chicken, turkey, fish, and plant-based proteins like beans and lentils.

5. Healthy fats: Use healthy fats like olive oil, avocados, and nuts in moderation.

6. Limit salt: Lower your salt intake to help reduce blood pressure. Aim for less than 2,300 mg of sodium per day.

7. Cut back on sugar: Limit added sugars from sweets, sugary drinks, and processed foods.

8. Stay hydrated: Drink plenty of water throughout the day.

9. Watch portion sizes: Eat smaller portions to help control your weight.

10. Limit alcohol: If you drink, do so in moderation – one drink per day for women and two for men.

Following this type of diet can help lower your blood pressure and cholesterol levels, improving your overall health. Remember, it's also important to exercise regularly and maintain a healthy weight.

***

If you have high blood pressure and high cholesterol, a good diet can help manage these issues. Here's a simple guide to follow:

1. Eat more fruits and vegetables: They're low in calories and high in vitamins, minerals, and fiber.

2. Choose whole grains: Go for whole wheat, brown rice, quinoa, or oats instead of refined grains like white bread and pasta.

3. Pick lean proteins: Opt for fish, skinless poultry, beans, lentils, or tofu instead of fatty meats.

4. Include healthy fats: Use olive oil, avocado, and nuts instead of butter or lard.

5. Limit salt: Aim for less than 2,300 mg of sodium per day. Cut back on processed foods and don't add extra salt to your meals.

6. Avoid added sugars: Skip sugary drinks and snacks. Stick to natural sources of sugar like fruits.

7. Eat low-fat dairy: Choose low-fat or fat-free milk, yogurt, and cheese.

8. Watch portion sizes: Don't eat too much at once. Use smaller plates to control portions.

9. Stay hydrated: Drink plenty of water throughout the day.

Remember, it's important to talk to your doctor or a dietitian before making any major changes to your diet. They can help you create a plan that's best for your specific needs.

***

If you have high blood pressure and high cholesterol, it's important to eat a healthy diet to help lower these levels. The best diet for you is one that's low in salt, saturated fat, and cholesterol, and rich in fruits, vegetables, whole grains, and lean protein sources. This is often called a heart-healthy diet.

Here are some easy tips for a heart-healthy diet:

1. Eat more fruits and vegetables: Aim for 5 servings a day. They're low in calories and packed with vitamins, minerals, and fiber.

2. Choose whole grains: Pick whole grain bread, pasta, and rice instead of white, refined versions. Whole grains have more nutrients and can help lower cholesterol.

3. Pick lean proteins: Eat more fish, skinless poultry, beans, and legumes. Limit red meat, as it can be high in saturated fat.

4. Use healthy fats: Cook with olive or canola oil instead of butter or lard. Choose nuts, seeds, and avocados for healthy fat sources.

5. Cut back on salt: Limit processed foods and don't add extra salt to your meals. High salt intake can raise blood pressure.

6. Watch portion sizes: Eating too much, even healthy foods, can lead to weight gain. Keep portion sizes in check.

7. Limit alcohol and avoid smoking: Drink alcohol in moderation, if at all, and quit smoking to improve heart health.

Remember, it's always best to talk to your doctor or a registered dietitian before making major changes to your diet. They can help you create a personalized plan that's right for your specific needs.

***

**How much should I exercise to stay healthy?**

**Answer this question using plain and easy-to-understand language in your response.**

*******

To stay healthy, aim for at least 150 minutes of moderate-intensity aerobic exercise or 75 minutes of vigorous-intensity aerobic exercise each week, spread across at least 3 days. Additionally, do muscle-strengthening activities for all major muscle groups at least 2 days per week. Listen to your body and adjust your routine as needed. Remember, some exercise is better than none, so start slowly and gradually increase intensity and duration.

*******

To stay healthy, aim for at least 150 minutes of moderate-intensity aerobic exercise or 75 minutes of vigorous-intensity aerobic exercise per week, spread over at least three days. Also, include strength training exercises for all major muscle groups at least twice a week. Remember to listen to your body and adjust your activity levels based on your fitness level, age, and any medical conditions. It's always a good idea to consult with a healthcare professional before starting a new exercise routine.

***

To stay healthy, aim for at least 150 minutes of moderate-intensity exercise or 75 minutes of vigorous-intensity exercise per week. You can also mix both types. Additionally, try to include strength training exercises for all major muscle groups at least two days a week. Remember, it's important to find activities you enjoy and spread your workouts throughout the week for the best results.

***

**Should I do cardio or lift weights to prevent heart disease?**

**Answer this question using plain and easy-to-understand language in your response.**

*******

Both cardio and lifting weights can help prevent heart disease, but they work in different ways. Cardio exercises, like running or biking, improve your heart's ability to pump blood and help lower blood pressure. Lifting weights can increase muscle mass, which can boost metabolism and help with weight control, reducing the risk of heart disease. It's a good idea to do a mix of both types of exercise for the best results. Aim for at least 150 minutes of moderate-intensity cardio and 2 days of strength training per week for a well-rounded workout routine. Always check with a doctor before starting a new exercise program, especially if you have any existing health issues.

***

Both cardio and weight lifting can help prevent heart disease. Cardio exercises, like running or swimming, improve heart health by strengthening the heart muscle and increasing blood flow. Weight lifting helps by lowering body fat, reducing blood pressure, and improving cholesterol levels. To get the best results, try to include both types of exercises in your routine. Aim for at least 150 minutes of moderate-intensity cardio or 75 minutes of vigorous-intensity cardio per week, and do strength training exercises for all major muscle groups at least two times per week.

***

Both cardio and weight lifting can help prevent heart disease. Cardio exercises, like running or swimming, are great for improving your heart health because they increase your heart rate and strengthen your heart muscle. Weight lifting, or resistance training, can also benefit your heart by reducing body fat, increasing muscle mass, and improving blood circulation. It's a good idea to include a mix of both types of exercise in your routine for the best results. Talk to your doctor before starting any exercise program to make sure it's safe for you.

***

**How can I lose weight?**

**Answer this question using plain and easy-to-understand language in your response.**

*******

To lose weight, try these simple steps:

1. Eat healthier: Choose fruits, veggies, whole grains, lean proteins, and low-fat dairy. Cut down on sugary, fatty, and processed foods.

2. Watch portion sizes: Eat smaller meals and avoid second helpings. Use smaller plates to help control portions.

3. Drink water: Stay hydrated by drinking plenty of water. It can help control hunger and replace sugary drinks.

4. Exercise regularly: Aim for at least 150 minutes of moderate exercise or 75 minutes of vigorous exercise each week. Mix cardio and strength training for best results.

5. Sleep well: Get 7-9 hours of sleep every night. Good sleep helps control hunger and supports weight loss.

6. Reduce stress: Find ways to manage stress, like meditation or hobbies, as stress can lead to overeating.

7. Stay consistent: Stick to your healthy habits even when progress is slow. Weight loss takes time and patience.

Remember to talk to a doctor before making big changes to your diet or exercise routine.

***

To lose weight, try these simple steps:

1. Eat healthier: Choose whole, unprocessed foods like fruits, vegetables, lean proteins, and whole grains. Cut down on sugary, fatty, and processed foods.

2. Watch portions: Eat smaller portions to control calorie intake. Using smaller plates can help.

3. Drink water: Drink water instead of sugary drinks. Water can also help you feel full.

4. Be active: Exercise regularly, aiming for at least 150 minutes of moderate activity per week. Try walking, swimming, or biking.

5. Sleep well: Get 7-9 hours of sleep every night. Good sleep helps control hunger and supports weight loss.

6. Reduce stress: Manage stress with relaxation techniques like deep breathing, meditation, or yoga.

7. Set goals: Set realistic and achievable weight loss goals. Track your progress to stay motivated.

Remember, it's important to consult a healthcare professional before starting any weight loss plan.

***

To lose weight, try these simple steps:

1. Eat less: Cut back on portion sizes and avoid extra snacking.

2. Choose healthy foods: Eat more vegetables, fruits, lean proteins, and whole grains.

3. Drink water: Replace sugary drinks with water to reduce calorie intake.

4. Move more: Exercise regularly, like walking, running, swimming, or biking.

5. Sleep well: Get enough sleep to help your body function properly.

6. Be patient: Weight loss takes time, so stick to your plan and don't give up.

***

**How can I decrease LDL? Answer this question using plain and easy-to-understand language in your response.**

*******

To lower your LDL (the "bad" cholesterol), try these simple tips:

1. Eat healthy: Choose fruits, vegetables, whole grains, and lean proteins. Cut down on saturated fats found in red meat, full-fat dairy, and fried foods.

2. Exercise: Aim for at least 30 minutes of moderate exercise, like brisk walking, most days of the week.

3. Lose weight: If you're overweight, losing even a few pounds can help lower your LDL.

4. Stop smoking: Quitting smoking can improve your cholesterol levels and overall heart health.

5. Limit alcohol: Stick to one drink per day for women and two drinks per day for men.

6. Add fiber: Eat foods high in fiber, like beans, oats, and whole grains, to help lower your LDL.

7. Consider supplements: Talk to your doctor about supplements, like fish oil or plant sterols, that can help lower LDL levels.

Remember, always consult your doctor before starting any new health regimen.

***

To lower your LDL (or "bad" cholesterol), try these simple steps:

1. Eat healthy: Choose more fruits, vegetables, whole grains, and lean proteins. Cut back on saturated fats found in red meat and full-fat dairy products. Instead, opt for healthier fats like those in fish, nuts, and olive oil.

2. Exercise: Aim for 30 minutes of physical activity most days of the week. This can include walking, swimming, or cycling.

3. Lose weight: If you're overweight, shedding some pounds can help lower your LDL levels.

4. Quit smoking: If you smoke, quitting can improve your cholesterol levels and overall heart health.

5. Limit alcohol: Drink alcohol in moderation, meaning up to one drink a day for women and two for men.

6. Check labels: Look for foods with low amounts of added sugars, trans fats, and sodium.

If lifestyle changes aren't enough, your doctor might suggest medications to help lower your LDL. Always talk to your doctor before starting any new treatments.

***

To lower your LDL (bad cholesterol), follow these simple steps:

1. Eat healthy: Choose whole, unprocessed foods like fruits, vegetables, whole grains, and lean protein. Avoid unhealthy fats, like trans and saturated fats, found in fried and processed foods.

2. Exercise: Aim for at least 150 minutes of moderate exercise each week. Walking, swimming, or biking are good choices.

3. Lose weight: If you're overweight, losing weight can help lower your LDL levels.

4. Stop smoking: If you smoke, quitting can improve your cholesterol levels and overall heart health.

5. Drink less alcohol: Limit your alcohol intake to one drink per day for women and two for men.

6. Talk to your doctor: They may recommend medication or other treatments if lifestyle changes aren't enough.

***

**How can I decrease triglycerides?**

**Answer this question using plain and easy-to-understand language in your response.**

*******

To lower triglycerides, try these simple steps:

1. Eat healthier: Choose more fruits, vegetables, whole grains, and lean proteins. Cut down on sugary foods and drinks.

2. Exercise: Aim for at least 30 minutes of physical activity most days of the week. Walking, swimming, or biking are good options.

3. Lose weight: If you're overweight, losing even a few pounds can help lower triglycerides.

4. Limit alcohol: Drinking less alcohol or none at all can help reduce triglyceride levels.

5. Avoid trans fats: Check food labels and avoid products with "partially hydrogenated oils" or "trans fats."

6. Choose good fats: Eat more foods with healthy fats like avocados, nuts, and olive oil instead of unhealthy fats found in fried foods and junk food.

7. Control blood sugar: If you have diabetes, keep your blood sugar in check by following your doctor's advice.

Remember, always talk to your doctor before making big changes to your diet or exercise routine.

***

To lower triglycerides, try these simple steps:

1. Eat healthier: Pick whole foods like fruits, veggies, and whole grains. Cut down on sugary treats, fast food, and processed items.

2. Less bad fats: Cut back on saturated fats in things like butter and fatty meats. Avoid trans fats found in some packaged foods.

3. More good fats: Add foods with good fats, like fish, nuts, seeds, and olive oil.

4. Exercise: Move your body more. Aim for at least 30 minutes a day, most days of the week.

5. Lose weight: If you're overweight, losing even a little can help lower triglycerides.

6. Limit alcohol: Cut down on alcohol or avoid it completely.

7. Stop smoking: If you smoke, quitting can help improve your heart health.

8. Check with your doctor: They might suggest medication or other treatments to help lower your triglycerides.

***

To lower triglycerides, try these simple steps:

1. Eat less sugar: Cut back on sugary foods and drinks, like candy, soda, and desserts.

2. Choose healthier fats: Pick unsaturated fats, like olive oil, instead of saturated fats, like butter.

3. Eat more fiber: Include foods like fruits, vegetables, and whole grains in your meals.

4. Exercise regularly: Aim for at least 150 minutes of moderate exercise per week, like brisk walking.

5. Lose weight: If you're overweight, try to lose a few pounds by eating healthier and exercising more.

6. Limit alcohol: Drink less alcohol or avoid it altogether, as it can raise triglyceride levels.

7. Eat fish: Include fish rich in omega-3 fatty acids, like salmon, in your diet at least twice a week.

Remember, it's important to talk to your doctor before making big changes to your diet or exercise routine.

***

**What is lipoprotein(a)?**

**Answer this question using plain and easy-to-understand language in your response.**

*******

Lipoprotein(a), also known as Lp(a), is a type of fat and protein particle found in our blood. It's similar to "bad" cholesterol or low-density lipoprotein (LDL), which can build up in blood vessels and cause heart problems. High levels of Lp(a) in our blood can increase the risk of heart disease, stroke, or other health issues. Doctors may check Lp(a) levels with a blood test, especially if someone has a family history of heart problems. Maintaining a healthy lifestyle with a balanced diet and regular exercise can help manage overall heart health.

***

Lipoprotein(a), often called Lp(a), is a kind of particle found in your blood. It's made up of two main parts: a fat-like substance (lipid) and a protein. Lp(a) is related to cholesterol, another fat-like substance in your blood. High levels of Lp(a) can be a problem because they can increase the risk of heart disease or stroke. In simple terms, Lp(a) is like a tiny, fatty blob floating around in your blood that can cause heart-related issues if there's too much of it.

***

Lipoprotein(a), or Lp(a) for short, is a kind of particle found in our blood. It's made up of fats (lipids) and proteins, and its main job is to transport cholesterol around the body. However, high levels of Lp(a) can be a problem because they can lead to the buildup of fatty deposits in our arteries. This can make it harder for blood to flow and increase the risk of heart disease or stroke. It's important to maintain healthy levels of Lp(a) to keep our heart and blood vessels working well.

***

**How can I quit smoking?**

**Answer this question using plain and easy-to-understand language in your response.**

*******

To quit smoking, follow these simple steps:

1. Decide: Make up your mind that you want to quit. Your determination is important.

2. Set a date: Choose a quit date to give yourself time to prepare.

3. Find support: Tell friends, family, or join a support group. They can help you stay strong.

4. Remove triggers: Get rid of cigarettes, lighters, and ashtrays from your home, car, and workplace.

5. Replace habits: Try chewing gum, eating healthy snacks, or drinking water when you feel like smoking.

6. Stay active: Exercise or go for walks to reduce cravings and stress.

7. Avoid temptations: Stay away from places or people that make you want to smoke.

8. Use aids: Nicotine patches, gum, or prescription medications can help with withdrawal symptoms. Talk to your doctor.

9. Be patient: Quitting is tough, and it might take a few tries. Don't give up if you slip.

10. Reward yourself: Celebrate your smoke-free milestones with treats or activities you enjoy.

***

Quitting smoking can be tough, but here are some simple steps to help you:

1. Decide: Make up your mind that you want to quit. Your determination is important.

2. Set a date: Pick a day in the near future to quit. Mark it on your calendar.

3. Tell people: Share your plan with friends and family. They can support and encourage you.

4. Remove cigarettes: Get rid of all smoking materials like cigarettes, ashtrays, and lighters.

5. Make a plan: Think about how you'll handle cravings. Use distractions or find healthier habits.

6. Stay busy: Keep your mind and body active to avoid thinking about smoking.

7. Avoid triggers: Stay away from situations, places, or people that make you want to smoke.

8. Use aids: Nicotine patches, gum, or medications can help. Talk to your doctor for advice.

9. Reward yourself: Celebrate your milestones without smoking. Treat yourself to something special.

10. Get help: Reach out to support groups, friends, family, or a therapist for encouragement.

Remember, quitting takes time and effort. Keep trying and stay positive, even if you slip up.

***

Quitting smoking can be tough, but here are some simple steps to help you:

1. Make a plan: Choose a date to quit and stick to it. Write down your reasons for quitting.

2. Find support: Tell friends, family, or coworkers about your plan. Join a support group or talk to a doctor or counselor.

3. Replace the habit: Find something else to do when you get the urge to smoke. Chew gum, drink water, or go for a walk.

4. Stay busy: Keep your mind off smoking by doing activities you enjoy or learning new things.

5. Avoid triggers: Stay away from places, people, or situations that make you want to smoke. Make a list of your triggers and find ways to avoid them.

6. Use quitting aids: Nicotine gum, patches, or lozenges can help reduce cravings. Talk to a doctor about prescription medications that might help.

7. Stay positive: Remind yourself why you're quitting and celebrate small milestones along the way.

8. Manage stress: Find healthy ways to deal with stress, like exercise, meditation, or deep breathing.

9. Be prepared for slip-ups: If you smoke, don't give up. Learn from your mistakes and keep trying.

10. Reward yourself: Treat yourself to something special with the money you save from not buying cigarettes.

***

**What are the side effects of statins?**

**Answer this question using plain and easy-to-understand language in your response.**

*******

Statins are medicines used to lower cholesterol levels in your blood. They can help prevent heart attacks and strokes. However, like other medicines, they can sometimes cause side effects. Here are some common ones in simple terms:

1. Muscle pain: You might feel soreness, weakness, or tenderness in your muscles. This is usually mild, but in rare cases, it can be severe.

2. Upset stomach: You may have stomach pain, gas, bloating, or nausea. These are usually not too serious.

3. Headaches: Some people get headaches when they start taking statins, but these often go away as your body gets used to the medicine.

4. Sleep problems: You might have trouble falling asleep or staying asleep, or you might have vivid dreams.

5. Dizziness: You could feel lightheaded or off-balance when you stand up or walk around.

6. Mild skin rash: Your skin might be itchy or have a slight rash. This usually isn't serious.

7. Liver issues: In rare cases, statins can cause liver problems. Your doctor might check your liver function with blood tests to make sure everything is okay.

8. Increased blood sugar: Statins can sometimes raise your blood sugar levels, which could be a concern if you have diabetes or are at risk for it.

If you notice any of these side effects, or if they get worse, talk to your doctor. They might adjust your dose or switch you to a different medicine. Remember, it's important to take statins as prescribed to keep your cholesterol in check and protect your heart.

***

Statins are medicines used to lower cholesterol in the blood. They can help prevent heart disease and stroke. But like all medicines, statins may cause side effects in some people. Here are some common side effects in simple language:

1. Muscle pain: Some people may feel sore or weak muscles when taking statins. This is usually mild, but it can be more severe in rare cases.

2. Headache: Statins might cause headaches for some people, but they usually go away after a while.

3. Upset stomach: Taking statins can sometimes lead to stomach problems like gas, bloating, or diarrhea.

4. Sleep problems: Some people may have trouble falling asleep or staying asleep when they take statins.

5. Dizziness: Feeling lightheaded or dizzy can happen to some people who use statins.

6. Skin rash: In rare cases, a person might get a rash or itchiness on their skin from statins.

It's important to remember that not everyone experiences side effects, and for most people, the benefits of taking statins outweigh the risks. If you think you have side effects from your statin, talk to your doctor. They can help you decide if you need to change your medicine or adjust the dose.

***

Statins are medicines often used to lower cholesterol levels in the blood. They can help prevent heart attacks and strokes. However, some people might experience side effects while taking them. Here are some common side effects in simple language:

1. Muscle pain: You might feel soreness or weakness in your muscles.

2. Headaches: You could have a mild to moderate headache.

3. Feeling sick: You might feel nauseous or want to vomit.

4. Stomach problems: You could have diarrhea, constipation, or tummy pain.

5. Sleep issues: You might have trouble sleeping or have nightmares.

6. Dizziness: You could feel lightheaded or dizzy.

7. Skin rash: You might notice red, itchy, or swollen skin.

Most of these side effects are not severe and might go away as your body gets used to the medicine. However, if they become bothersome or don't go away, talk to your doctor. They might adjust your dose or try a different medicine.

***

**I have muscle pain with a statin. What should I do?**

**Answer this question using plain and easy-to-understand language in your response.**

*******

I'm not a doctor, but if you're experiencing muscle pain while taking a statin, you might want to consider the following steps:

1. Talk to your doctor: Let your doctor know about your symptoms, as they can help you figure out if the pain is related to the statin or another issue.

2. Don't stop the medication on your own: It's important not to stop taking your statin without discussing it with your doctor first, as they can guide you on the best course of action.

3. Over-the-counter pain relief: You could try taking over-the-counter pain relievers like acetaminophen or ibuprofen to help with the discomfort. But remember to ask your doctor before taking any new medication.

4. Stay hydrated and stretch: Drinking plenty of water and doing gentle stretches may help alleviate muscle pain.

Always consult your doctor for personalized advice and recommendations.

***

I'm not a doctor, but if you're experiencing muscle pain while taking a statin, you might want to consider the following steps:

1. Talk to your doctor: Let them know about your symptoms. They may adjust your dosage, recommend a different statin, or suggest another treatment option.

2. Take it easy: Rest and avoid overexerting your muscles while you're experiencing pain.

3. Over-the-counter pain relief: You could try taking over-the-counter pain relievers, like ibuprofen, to help with the pain. But remember to ask your doctor first.

Always follow your doctor's advice, as they know your medical history and can give you the best guidance.

***

I'm not a doctor, but if you're experiencing muscle pain while taking a statin, you might consider the following steps:

1. Talk to your doctor: Explain your symptoms and concerns. They can help determine if the pain is related to the statin or another issue.

2. Don't stop taking the medication without consulting your doctor: Stopping suddenly could have negative effects on your health.

3. Track your symptoms: Keep a record of when the pain occurs, its severity, and any other relevant details. This can help your doctor assess the situation.

4. Stay active and hydrated: Regular exercise and proper hydration may help reduce muscle pain.

5. Follow your doctor's advice: They may adjust your medication, dosage, or recommend other treatments to manage the pain.

Remember to always consult with a healthcare professional before making any changes to your medications or health routines.

***

**My cholesterol is still high and I’m already on a statin. What should I do?**

**Answer this question using plain and easy-to-understand language in your response.**

*******

I'm not a doctor, but I can try to offer some general advice in simple terms. If your cholesterol is still high even though you're taking a statin, you might want to consider the following steps:

1. Talk to your doctor: Share your concerns with your healthcare provider. They might adjust your medication, recommend other treatments, or explore possible underlying causes.

2. Improve your diet: Try to eat more fruits, vegetables, whole grains, and lean proteins. Cut down on saturated and trans fats, found in foods like butter, fried foods, and pastries.

3. Exercise regularly: Aim for at least 30 minutes of moderate-intensity exercise most days of the week. This can help increase your "good" cholesterol and lower your "bad" cholesterol.

4. Maintain a healthy weight: Losing excess weight can help lower your cholesterol levels. Talk to your doctor about a weight loss plan that works for you.

5. Stop smoking: If you smoke, quitting can improve your cholesterol levels and overall heart health.

6. Limit alcohol intake: Drink alcohol in moderation or not at all. Too much alcohol can raise your cholesterol levels.

Remember, it's important to consult with your doctor before making any changes to your diet, exercise, or medication regimen.

***

I'm not a doctor, but I can try to provide some general advice in simple terms. If you're already taking a statin and your cholesterol is still high, it might be helpful to consider the following steps:

1. Talk to your doctor: They can assess your situation, adjust your medication if needed, and give personalized advice.

2. Eat a healthy diet: Focus on eating more fruits, vegetables, whole grains, and lean protein. Limit saturated fats, trans fats, and cholesterol.

3. Exercise: Aim for at least 30 minutes of moderate exercise most days of the week. This can help improve your cholesterol levels.

4. Lose weight: If you're overweight, losing even a few pounds can help improve your cholesterol.

5. Stop smoking: If you smoke, quitting can help improve your cholesterol and overall health.

6. Limit alcohol: Drink in moderation or not at all, as excessive alcohol intake can raise cholesterol levels.

Remember, it's important to consult with your healthcare professional for personalized advice and to discuss any changes you want to make in your lifestyle or medication.

***

I'm not a doctor, but I can try to provide some general suggestions. If your cholesterol is still high despite taking a statin, you might want to consider:

1. Talking to your doctor: They can assess your situation, adjust your medication or recommend additional treatments.

2. Lifestyle changes: Adopting a healthier lifestyle can help lower cholesterol. This may include:

a. Eating a heart-healthy diet: Choose more fruits, vegetables, whole grains, lean protein, and low-fat dairy products.

b. Exercising regularly: Aim for at least 150 minutes of moderate-intensity exercise per week.

c. Maintaining a healthy weight: If you're overweight, losing weight can help lower cholesterol levels.

d. Quitting smoking: If you smoke, quitting can improve your cholesterol levels and overall heart health.

e. Limiting alcohol: Drinking alcohol in moderation or not at all can help control cholesterol levels.

Remember, it's important to consult with your doctor about any changes to your treatment plan or lifestyle.

***

**What medications can reduce cholesterol other than statins?**

**Answer this question using plain and easy-to-understand language in your response.**

***

There are several medications that can help lower cholesterol besides statins. Some of these include:

1. Bile acid sequestrants: These medications help your body get rid of cholesterol by binding to bile acids in your intestines. Examples include cholestyramine, colestipol, and colesevelam.

2. Fibrates: These drugs lower the levels of triglycerides, a type of fat in your blood, and can slightly increase good cholesterol (HDL). Examples include gemfibrozil and fenofibrate.

3. Niacin (vitamin B3): This vitamin can help lower bad cholesterol (LDL) and triglycerides while raising good cholesterol (HDL). High doses of niacin are needed for this effect, which can be found in prescription forms like Niaspan.

4. Ezetimibe: This medication works by reducing the amount of cholesterol your body absorbs from food. It can be used alone or with a statin.

5. PCSK9 inhibitors: These injectable drugs help your liver remove more bad cholesterol from your blood. Examples include evolocumab and alirocumab.

Always talk to your doctor before starting any new medication, as they can help you decide which one is best for your specific situation.

***

There are several medications that can help lower cholesterol levels apart from statins. Some common alternatives include:

1. Fibrates: These medicines help lower triglyceride levels and can raise HDL (good) cholesterol. Examples are fenofibrate and gemfibrozil.

2. Niacin: Also known as vitamin B3, niacin can help lower LDL (bad) cholesterol and triglycerides and raise HDL cholesterol. It is available in prescription and over-the-counter forms.

3. Bile acid binding resins: These drugs work by binding to bile acids in your intestines, so your liver uses more cholesterol to make bile, which lowers the amount of cholesterol in your blood. Examples are cholestyramine, colestipol, and colesevelam.

4. Cholesterol absorption inhibitors: These medications, like ezetimibe, reduce the absorption of cholesterol from food in the intestines, which lowers the level of cholesterol in your blood.

5. PCSK9 inhibitors: These injectable drugs, such as evolocumab and alirocumab, can help lower LDL cholesterol by increasing the liver's ability to remove LDL cholesterol from the blood.

Remember that it's important to talk to your doctor before starting any new medication. They will help you find the best treatment plan based on your specific health needs and conditions.

***

There are several medications that can help reduce cholesterol levels other than statins. Here are some of them:

1. Bile acid-binding resins: These drugs, like cholestyramine, colestipol, and colesevelam, work by binding to bile acids in your intestines. This helps your liver use up more cholesterol to make new bile acids, lowering cholesterol levels in your blood.

2. Cholesterol absorption inhibitors: Ezetimibe is an example of this type of drug. It reduces the amount of cholesterol absorbed by your intestines, which helps to lower cholesterol levels in your blood.

3. Fibrates: Medications like gemfibrozil and fenofibrate can help reduce triglycerides, a type of fat in your blood, and may also slightly increase good cholesterol (HDL) levels.

3. Niacin (vitamin B3): Niacin can help lower bad cholesterol (LDL) and triglycerides while increasing good cholesterol (HDL) levels. However, it can have side effects, so it's important to talk to your doctor before taking it.

3. PCSK9 inhibitors: These injectable drugs, such as alirocumab and evolocumab, can help reduce LDL cholesterol by increasing its removal from your blood. They are typically used for people who cannot tolerate statins or need additional cholesterol-lowering help.

Remember, it's essential to talk to your healthcare provider before starting any new medications, as they can provide personalized advice based on your medical history and needs.

***

**What is ezetimibe?**

**Answer this question using plain and easy-to-understand language in your response.**

***

Ezetimibe is a medicine used to help lower bad cholesterol levels in the blood. It works by stopping the body from absorbing too much cholesterol from the food we eat. This helps keep the heart healthy and reduces the risk of heart problems. People often take it along with a healthy diet and exercise to improve their cholesterol levels.

***

Ezetimibe is a medicine used to help lower the amount of bad cholesterol in your blood. It works by stopping your body from absorbing too much cholesterol from the food you eat. Doctors often prescribe this medicine for people with high cholesterol to reduce the risk of heart problems. It's usually taken as a pill, and sometimes it's combined with other medicines to be even more effective.

***

Ezetimibe is a medicine that helps lower the levels of bad cholesterol in your body. Bad cholesterol, also called LDL cholesterol, can build up in your blood vessels and cause problems like heart disease. Ezetimibe works by stopping your body from absorbing as much bad cholesterol from the food you eat. This helps to keep your blood vessels healthier and reduces the risk of heart-related issues. Doctors often prescribe ezetimibe along with a healthy diet and sometimes other medications to help people manage their cholesterol levels.

*******

**What are Repatha and Praluent?**

**Answer this question using plain and easy-to-understand language in your response.**

***

Repatha and Praluent are medicines that help lower bad cholesterol levels in the blood. They belong to a group of drugs called PCSK9 inhibitors. These drugs work by helping the liver to remove more bad cholesterol, called LDL cholesterol, from the blood. This helps reduce the risk of heart disease and stroke. Doctors often prescribe these medicines to people who have high cholesterol that's not well-controlled by other treatments, like diet, exercise, or other cholesterol-lowering drugs.

***

Repatha and Praluent are medicines used to lower bad cholesterol, also known as LDL cholesterol, in the blood. They are mainly given to people who have a high risk of heart disease or who can't lower their cholesterol enough with other treatments, like diet changes or statins. Both medicines work by blocking a protein called PCSK9, which helps the body get rid of more LDL cholesterol. As a result, cholesterol levels in the blood go down, reducing the risk of heart problems.

***

Repatha and Praluent are medications that help lower bad cholesterol levels in your blood. They belong to a class of drugs called PCSK9 inhibitors. These medicines work by blocking a protein called PCSK9, which is involved in controlling cholesterol levels. By blocking this protein, they help your liver remove more bad cholesterol from your blood, reducing the risk of heart problems like heart attack or stroke. Doctors often prescribe Repatha and Praluent to patients who have high cholesterol and are at a higher risk for heart diseases, especially when other cholesterol-lowering medications haven't worked well enough.

*******

**What is inclisiran?**

**Answer this question using plain and easy-to-understand language in your response.**

***

Inclisiran is a medicine used to help lower bad cholesterol levels in the blood. Bad cholesterol, also known as LDL cholesterol, can cause problems in your blood vessels and lead to heart disease. Inclisiran works by blocking a specific molecule in the body, which in turn helps the liver remove more bad cholesterol. This medicine is usually given as an injection and can be used along with other treatments, like a healthy diet and exercise, to improve heart health.

***

Inclisiran is a medicine used to lower cholesterol levels in the blood. It works by blocking a specific substance in the liver that helps make cholesterol. By doing this, it helps reduce the amount of bad cholesterol in the body, which can lower the risk of heart disease and other health problems. Doctors usually prescribe inclisiran to people who have high cholesterol and have tried other treatments without success.

***

Inclisiran is a medication that helps lower the levels of bad cholesterol, or LDL cholesterol, in a person's blood. It does this by blocking a specific substance in the body, which in turn reduces cholesterol production. People with high cholesterol are at a higher risk for heart problems, so lowering their cholesterol with inclisiran can help keep their hearts healthy. This medicine is given through an injection, usually twice a year.

*******

**What are the side effects of Repatha and Praluent?**

**Answer this question using plain and easy-to-understand language in your response.**

***

Repatha and Praluent are medications used to lower bad cholesterol levels in the blood. They both belong to a class of drugs called PCSK9 inhibitors. Here are some possible side effects of these medications in simple language:

1. Pain or redness at the injection site: You might feel some discomfort or see redness where you injected the medicine.

2. Cold or flu-like symptoms: You could experience symptoms like a stuffy nose, sore throat, or sneezing.

3. Allergic reactions: Some people may have an allergic reaction, with symptoms like rash, itching, or swelling. If you have trouble breathing or feel very unwell, contact a doctor right away.

4. Muscle pain or weakness: You might feel sore or weak in your muscles.

5. Back pain: Some people may have pain in their back.

Remember that not everyone will experience these side effects, and some people might not have any at all. If you are concerned about any side effects or have questions, it's important to talk to your healthcare provider.

***

Repatha and Praluent are medications used to lower bad cholesterol levels in the blood. They can have some side effects, but not everyone experiences them. Here are some common and less common side effects of these drugs:

1. Common side effects:

• Redness, swelling, or pain at the injection site: This is where the medicine is injected, and it might be a bit sore or irritated.

• Cold symptoms: You might have a stuffy nose, sneezing, or a sore throat.

• Flu-like symptoms: Feeling tired, feverish, or having body aches can happen too.

2. Less common side effects:

• Allergic reactions: Some people might have a rash, itching, or trouble breathing. If this happens, you should call your doctor right away.

• Muscle pain or weakness: Your muscles might hurt or feel weak, which can be a sign of a more serious problem. Talk to your doctor if this happens.

Remember, it's important to discuss any side effects with your healthcare provider, as they can help you manage them and decide if these medications are right for you.

***

Repatha and Praluent are medications used to lower bad cholesterol levels in the blood. They both belong to a group of drugs called PCSK9 inhibitors. Here are some possible side effects you might experience when taking these medications:

1. Pain or swelling at the injection site: You might feel soreness, redness, or swelling where the medicine was injected.

2. Cold-like symptoms: You could experience a stuffy or runny nose, sneezing, or a sore throat.

3. Flu-like symptoms: You might feel feverish, have body aches, or feel generally unwell.

4. Allergic reactions: In rare cases, some people might develop rashes, itching, or swelling, or have trouble breathing.

Remember, not everyone experiences side effects, and they can vary from person to person. If you have concerns or experience severe side effects, contact your doctor for advice.

*******

**Should I take aspirin to prevent heart disease?**

**Answer this question using plain and easy-to-understand language in your response.**

*******

I'm not a doctor, but I can provide you with some general information. It's important to talk to your healthcare provider before making any decisions about medications.

Aspirin has been known to help prevent heart attacks and strokes in some people by thinning the blood and reducing the risk of blood clots. However, taking aspirin regularly isn't recommended for everyone, as it can have side effects and increase the risk of bleeding.

Your healthcare provider is the best person to help you determine if taking aspirin is right for you. They can consider your individual risk factors, medical history, and any other medications you might be taking to make an informed decision.

***

I'm not a doctor, but I can provide some general information. Aspirin may be helpful for some people in preventing heart disease, but it's not right for everyone. You should talk to your doctor about your specific situation and risk factors to determine if taking aspirin for heart disease prevention is appropriate for you.

***

I'm not a doctor, but I can provide some general information. Some people take low-dose aspirin to help prevent heart attacks, but it's not suitable for everyone. You should talk to your doctor before starting any new medication, as they can consider your individual risk factors and medical history. They'll help determine if taking aspirin is appropriate for you. Remember, always consult a healthcare professional for personalized advice.

***

**My cholesterol panel shows triglycerides 400 mg/dL.**

**How should I interpret this?**

**Answer this question using plain and easy-to-understand language in your response.**

***

Your triglyceride level of 400 mg/dL is considered high. Triglycerides are a type of fat in your blood. High levels can increase the risk of heart disease and other health problems. To improve your levels, consider eating a healthier diet, exercising regularly, and talking to your doctor for further advice.

***

I'm not a doctor, but I can provide some general information. A triglycerides level of 400 mg/dL is considered high. Triglycerides are a type of fat found in your blood. When you eat, your body converts calories it doesn't need into triglycerides and stores them in fat cells. High triglyceride levels may increase your risk of heart disease and other health issues. It's a good idea to talk to your doctor for personalized advice on managing your triglycerides level.

***

I'm not a doctor, but I can help you understand what it might mean if your triglycerides are at 400 mg/dL. Triglycerides are a type of fat found in your blood. A level of 400 mg/dL is considered very high. High triglycerides can be a risk factor for heart disease and other health issues. It's important to talk to your doctor about your results and what steps you can take to lower your triglycerides, which might include changes in your diet, exercise, or medications.

***

**My LDL is 200 mg/dL. How should I interpret this?**

**Answer this question using plain and easy-to-understand language in your response.**

*******

Your LDL level of 200 mg/dL is considered high. LDL stands for low-density lipoprotein, and it's often called "bad" cholesterol. High LDL levels can lead to a buildup of cholesterol in your arteries, which increases your risk for heart disease and stroke. It's a good idea to talk to your doctor about ways to lower your LDL, which may include a healthier diet, exercise, and possibly medication.

***

Your LDL (low-density lipoprotein) level is 200 mg/dL, which is considered high. LDL is often called "bad" cholesterol because it can build up in your arteries and increase your risk of heart disease. A high LDL level means you might need to make lifestyle changes or work with your doctor to manage your cholesterol levels and reduce your risk of heart-related problems.

***

Your LDL cholesterol level is 200 mg/dL, which means you have 200 milligrams of low-density lipoprotein (LDL) cholesterol in every deciliter of blood. LDL is often called "bad" cholesterol because it can build up in your arteries, increasing your risk for heart disease.

An LDL level of 200 mg/dL is considered high. In simple terms, this means that your LDL cholesterol is higher than what is generally recommended for a healthy heart. It's a good idea to talk to your doctor about ways to lower your LDL cholesterol, which may include changes to your diet, exercise habits, or possibly medication.

***

**What does a coronary calcium score of 0 mean?**

**Answer this question using plain and easy-to-understand language in your response.**

*******

A coronary calcium score of 0 means that there is no visible calcium in the arteries of your heart. This is a good thing, because it suggests a low risk of having heart problems or a heart attack in the near future. In simple terms, a score of 0 means your heart arteries are clear and you likely have a healthy heart.

*******

A coronary calcium score of 0 means that there is no visible calcium in the heart arteries. This is good news, as it suggests a low risk of heart problems. In simple terms, a score of 0 means your heart arteries are clear of calcium buildup, which is a sign of healthy blood flow and a lower chance of future heart issues.

*******

A coronary calcium score of 0 means that no calcium deposits were found in the arteries of your heart. This is good news because it suggests a low risk of having a heart attack or other heart problems in the near future. In simple terms, a score of 0 means your heart arteries are clear of calcium buildup, which is a positive sign for your heart health.

*******

**What does a coronary calcium score of 100 mean?**

**Answer this question using plain and easy-to-understand language in your response.**

*******

A coronary calcium score of 100 means that there is a moderate amount of calcium buildup in the arteries around your heart. Calcium buildup can be a sign of plaque in your arteries, which can increase your risk of heart problems. A score of 100 doesn't mean you have heart disease, but it suggests you might be at a higher risk than someone with a lower score. It's important to talk to your doctor about your score and what steps you can take to improve your heart health.

*******

A coronary calcium score of 100 means there is a moderate amount of calcium buildup in the heart arteries. Calcium in the arteries is a sign of plaque, which can narrow or block blood flow. A score of 100 puts a person at a higher risk for heart problems than those with lower scores. It's important for anyone with a score of 100 to talk with their doctor about ways to reduce their risk for heart issues, such as making lifestyle changes or taking medication.

*******

A coronary calcium score of 100 means that there is a moderate amount of calcium in the arteries of your heart. Calcium can build up in your arteries over time, making them harder and narrower. This can slow down or block blood flow to your heart, which may lead to heart problems.

A score of 100 is considered a medium risk for future heart issues. It's important to talk to your doctor about what this score means for you and how to lower your risk of heart problems. They may suggest lifestyle changes, like eating healthier and exercising more, or medications to help keep your heart healthy.

*******

**What does a coronary calcium score of 400 mean?**

**Answer this question using plain and easy-to-understand language in your response.**

*******

A coronary calcium score of 400 means that there is a significant amount of calcium buildup in the arteries of your heart. This buildup can narrow the arteries, making it harder for blood to flow and potentially leading to heart problems. A score of 400 is considered high and suggests a higher risk of having a heart attack or other heart issues. It's important to talk to your doctor about this score, as they can recommend the best ways to lower your risk and improve your heart health.

***

A coronary calcium score of 400 means there is a significant amount of calcium in the heart arteries, which can be a sign of heart disease. Calcium in the arteries can make them harder and narrower, making it difficult for blood to flow to the heart. This can increase the risk of heart attacks and other heart problems. A score of 400 suggests a higher risk, so it is important to talk to a doctor about steps to improve heart health and lower the risk of heart issues.

***

A coronary calcium score of 400 means that there is a significant amount of calcium buildup in the arteries of your heart. This score suggests that you have a higher risk of heart-related problems, like heart attacks or strokes. It's important to talk to your doctor about ways to reduce this risk, which may include lifestyle changes or medications.

***

**What genetic mutations can cause high cholesterol?**

**Answer this question using plain and easy-to-understand language in your response.**

***

High cholesterol can be caused by genetic mutations, which means changes in your genes. Two main genetic conditions can lead to high cholesterol:

1. Familial Hypercholesterolemia (FH): This is a common inherited condition where a person's body has trouble removing "bad" cholesterol (called LDL cholesterol) from the blood. As a result, the LDL cholesterol levels become very high, increasing the risk of heart disease. FH is caused by changes in genes like LDLR, APOB, or PCSK9.

2. Familial Combined Hyperlipidemia (FCH): This is a less common inherited condition that causes both high "bad" cholesterol (LDL) and high triglycerides (another type of fat in the blood). FCH can also increase the risk of heart disease. The exact genes involved in FCH are not well-known, but it is thought to involve multiple genes.

In summary, some people have high cholesterol because of genetic conditions like FH or FCH, which are caused by changes in their genes. These conditions can increase the risk of heart disease, so it's important to manage cholesterol levels with the help of a doctor.

***

High cholesterol can be caused by genetic mutations, which are changes in our DNA. There are a few key mutations that may lead to high cholesterol levels:

1. Familial hypercholesterolemia (FH): This is the most common genetic cause of high cholesterol. In people with FH, a gene responsible for removing bad cholesterol (LDL) from the blood doesn't work properly, leading to high LDL levels.

2. Familial combined hyperlipidemia (FCHL): This mutation affects how your body processes fats. It can cause high levels of both bad cholesterol (LDL) and another type of fat called triglycerides.

3. Polygenic hypercholesterolemia: This is caused by a combination of small genetic changes that together increase cholesterol levels. It's not as severe as FH or FCHL but can still cause high cholesterol.

In all cases, maintaining a healthy lifestyle, like eating well and exercising, can help control cholesterol levels. However, some people may also need medication or other treatments.

***

High cholesterol can be caused by genetic mutations, which means changes in certain genes that we inherit from our parents. Two common genetic mutations linked to high cholesterol are:

1. Familial Hypercholesterolemia (FH): This mutation affects a gene responsible for helping remove "bad" cholesterol (called LDL) from the blood. When this gene doesn't work properly, LDL levels can become very high, increasing the risk of heart problems.

2. Familial Combined Hyperlipidemia (FCH): This mutation affects how the body processes both "bad" cholesterol (LDL) and "good" cholesterol (HDL), as well as triglycerides (a type of fat in the blood). People with FCH often have higher levels of LDL and triglycerides, and lower levels of HDL, which can lead to heart issues.

In both cases, these genetic mutations can cause high cholesterol even if a person has a healthy diet and lifestyle. It's important to talk to a doctor if you think you might have a genetic mutation causing high cholesterol. They can help you figure out the best way to manage it and lower your risk of heart problems.

**Plain Language (One-Shot)**

**How can I prevent heart disease?**

**Answer this question using plain and easy-to-understand language in your response. This text about COVID-19 is a good example of the writing style (not content) you should use.**

**What is COVID-19?**

**• It is a new illness spreading around the world.**

**• It’s nickname is coronavirus.**

**How do you get it?**

**• Someone with COVID-19 gives you their germs.**

**• When they cough or sneeze, their germs get in the air, on you, and on things.**

**• Germs get into your body through your mouth, nose, and your eyes.**

***

How can you prevent heart disease?

What is heart disease?

• It is a health problem affecting your heart.

• It can lead to heart attacks, strokes, and other issues.

How can you stop it?

1. Eat healthy food:

• Choose fruits, veggies, whole grains, and lean meats.

• Limit salt, sugar, and unhealthy fats.

2. Exercise more:

• Move your body for 30 minutes most days.

• Walking, swimming, and dancing are good options.

3. Don't smoke:

• Smoking is bad for your heart.

• If you smoke, try to quit. Ask for help if you need it.

4. Watch your weight:

• Extra weight can hurt your heart.

• A healthy diet and exercise can help you lose weight.

5. Limit alcohol:

• Drinking too much can harm your heart.

• Stick to 1-2 drinks a day for men, 1 for women.

6. Control stress:

• Stress can hurt your heart.

• Try deep breathing, exercise, or talking to a friend.

7. Check your blood pressure:

• High blood pressure is bad for your heart.

• Get it checked regularly and follow your doctor's advice.

Remember to talk to your doctor about your heart health. They can help you make a plan to keep your heart strong and healthy.

***

How can I prevent heart disease?

What is heart disease?

• It is a group of problems that affect your heart and blood vessels.

• It can make your heart weak and not work properly.

How can I lower my risk?

1. Eat healthy food:

• Choose fruits, vegetables, whole grains, and lean proteins.

• Limit salt, sugar, and unhealthy fats.

2. Exercise regularly:

• Aim for at least 30 minutes most days of the week.

• You can walk, run, swim, or do any activity you enjoy.

3. Don't smoke:

• If you smoke, try to quit. It's never too late to stop.

• Ask your doctor for help if you need it.

4. Watch your weight:

• Extra weight makes your heart work harder.

• Eat healthy and exercise to reach a healthy weight.

5. Limit alcohol:

• Drink alcohol in moderation, if at all.

• Too much alcohol can hurt your heart.

6. Manage stress:

• Find ways to relax and handle stress, like deep breathing or meditation.

• Talk to someone if you feel overwhelmed.

7. Check your blood pressure:

• High blood pressure can harm your heart.

• Get it checked and follow your doctor's advice to keep it in a good range.

8. Control diabetes:

• If you have diabetes, work with your doctor to manage it.

• Keep your blood sugar in check to protect your heart.

Remember, small steps can make a big difference in keeping your heart healthy!

***

How can you prevent heart disease?

What is heart disease?

• It is a group of problems that affect your heart.

• It can make your heart work poorly or stop working.

How can you avoid it?

1. Eat healthy food:

• Choose fruits, vegetables, and whole grains.

• Pick lean meats like chicken or fish.

• Cut down on salt, sugar, and bad fats.

2. Exercise often:

• Try to move your body for at least 30 minutes a day.

• You can walk, swim, dance, or play a sport.

3. Don't smoke:

• Smoking is very bad for your heart.

• If you smoke, try to quit. Ask for help if you need it.

4. Watch your weight:

• Extra weight can strain your heart.

• Try to stay at a healthy weight for your body.

5. Limit alcohol:

• Drinking too much alcohol can hurt your heart.

• Stick to one drink a day for women, two for men.

6. Control stress:

• Too much stress can raise your blood pressure.

• Find ways to relax, like deep breathing or meditation.

7. Get regular check-ups:

• See your doctor to check your heart health.

• They can help you catch problems early and make a plan to stay healthy.

***

**What is the best diet for the heart?**

**Answer this question using plain and easy-to-understand language in your response. This text about COVID-19 is a good example of the writing style (not content) you should use.**

**What is COVID-19?**

**• It is a new illness spreading around the world.**

**• It’s nickname is coronavirus.**

**How do you get it?**

**• Someone with COVID-19 gives you their germs.**

**• When they cough or sneeze, their germs get in the air, on you, and on things.**

**• Germs get into your body through your mouth, nose, and your eyes.**

***

What is the best diet for the heart?

• It is a way of eating that helps keep your heart healthy.

• It's called a heart-healthy diet.

What should you eat?

• Eat lots of fruits and vegetables.

• Choose whole grains like brown rice and whole wheat bread.

• Pick lean meats like chicken and fish, and avoid fatty meats.

• Eat nuts, seeds, and legumes like beans and lentils.

• Limit foods high in salt, sugar, and unhealthy fats.

What should you avoid?

• Cut down on processed foods like chips and cookies.

• Stay away from sugary drinks like soda.

• Don't eat too much fast food or fried food.

***

What is the best diet for the heart?

• It is called a heart-healthy diet.

• It helps keep your heart strong and working well.

What foods should you eat?

• Eat lots of fruits and vegetables.

• Choose whole grains like brown rice and whole wheat bread.

• Pick lean protein like fish, chicken, and beans.

• Use healthy fats like olive oil and nuts.

What foods should you avoid?

• Don't eat too much salt.

• Stay away from lots of sugar and sweets.

• Avoid bad fats like those found in fried foods and butter.

• Limit processed foods, like chips and packaged snacks.

How can this diet help your heart?

• It can lower your blood pressure.

• It helps control your weight.

• It can lower your risk of heart problems.

***

What is the best diet for the heart?

• Eat foods that are good for your heart.

• A good diet can help your heart stay healthy.

What should you eat?

• Lots of fruits and vegetables.

• Whole grains like bread, rice, and pasta.

• Healthy proteins like fish, beans, and chicken.

• Good fats like olive oil, nuts, and avocado.

What should you avoid?

• Too much salt.

• Sugary foods and drinks.

• Unhealthy fats like fried foods and butter.

• Red meat like beef and pork.

Remember:

• Drink water instead of sugary drinks.

• Watch your portion sizes.

• Exercise and stay active for a healthy heart.

***

**What is the best diet for high blood pressure and high cholesterol?**

**Answer this question using plain and easy-to-understand language in your response. This text about COVID-19 is a good example of the writing style (not content) you should use.**

**What is COVID-19?**

**• It is a new illness spreading around the world.**

**• It’s nickname is coronavirus.**

**How do you get it?**

**• Someone with COVID-19 gives you their germs.**

**• When they cough or sneeze, their germs get in the air, on you, and on things.**

**• Germs get into your body through your mouth, nose, and your eyes.**

***

What is the best diet for high blood pressure and high cholesterol?

• Eat a diet with lots of fruits, vegetables, and whole grains.

• Choose foods low in salt and added sugars.

• Go for healthy fats like olive oil, nuts, and fish.

• Cut back on red meat and fatty foods.

Why is this diet good?

• Fruits and vegetables have vitamins and minerals that help your heart.

• Whole grains are good for your heart and help you feel full.

• Low-salt foods help keep your blood pressure down.

• Healthy fats can lower bad cholesterol and raise good cholesterol.

What should you avoid?

• Stay away from too much salt, sugar, and unhealthy fats.

• Don't eat a lot of fried foods or fast food.

• Skip processed meats like hot dogs and deli meats.

• Limit your alcohol and avoid smoking.

***

What is the best diet for high blood pressure and high cholesterol?

• Eat a healthy diet to help your body.

• A good diet is called DASH. It stands for Dietary Approaches to Stop Hypertension.

What should you eat?

• Eat lots of fruits and veggies.

• Have whole grains like brown rice and whole wheat bread.

• Pick lean proteins like chicken, fish, and beans.

• Try low-fat dairy products like yogurt and milk.

What should you avoid?

• Don't eat too much salt. Use herbs and spices instead.

• Stay away from fatty foods like fried foods and fatty meats.

• Don't have too much sugar, like candy and sweet drinks.

Remember to drink water, exercise, and talk to your doctor for more help.

***

What is the best diet for high blood pressure and high cholesterol?

• The best diet is called the DASH diet.

• DASH stands for Dietary Approaches to Stop Hypertension.

What does the DASH diet include?

• Eat lots of fruits and vegetables.

• Have whole grains, like brown rice and whole wheat bread.

• Pick lean meats, like chicken and fish.

• Enjoy low-fat dairy, like skim milk and yogurt.

What should you avoid?

• Don't eat too much salt.

• Cut back on fatty foods, like fried stuff and butter.

• Stay away from sweets and sugary drinks.

How can it help?

• This diet can lower your blood pressure and cholesterol.

• It can make your heart healthier and help you feel better.

***

**How much should I exercise to stay healthy?**

**Answer this question using plain and easy-to-understand language in your response. This text about COVID-19 is a good example of the writing style (not content) you should use.**

**What is COVID-19?**

**• It is a new illness spreading around the world.**

**• It’s nickname is coronavirus.**

**How do you get it?**

**• Someone with COVID-19 gives you their germs.**

**• When they cough or sneeze, their germs get in the air, on you, and on things.**

**• Germs get into your body through your mouth, nose, and your eyes.**

*******

How much should you exercise to stay healthy?

• Exercise is important to keep your body strong and happy.

• You should do some exercise every day.

What kind of exercise should you do?

• Mix different types of exercise like walking, running, swimming, or playing sports.

• Don't forget to stretch your muscles and do some exercises to make them stronger.

How long should you exercise?

• Adults should exercise for at least 150 minutes a week, which means about 30 minutes a day, 5 days a week.

• Kids should play and move for at least 60 minutes a day.

Remember:

• It's good to start slow and do a little more each time.

• Have fun and choose activities you enjoy.

• Don't forget to drink water and take breaks when you need them.

*******

How much should you exercise to stay healthy?

• You should exercise a few times each week.

• Aim for about 30 minutes each time.

What types of exercise should you do?

• Mix it up with activities like walking, running, swimming, and biking.

• You can also try yoga, dancing, or playing sports.

Why is exercise good for you?

• It helps your heart, lungs, and muscles get stronger.

• It can make you feel happier and less stressed.

• It can help you sleep better and maintain a healthy weight.

***

How much exercise do you need to stay healthy?

• Try to be active every day.

• Aim for at least 150 minutes a week of moderate exercise or 75 minutes a week of intense exercise.

• You can break it into smaller sessions, like 30 minutes a day, 5 days a week.

What is moderate exercise?

• It's when you move your body and feel your heart beat faster.

• Examples: walking fast, dancing, or riding a bike.

What is intense exercise?

• It's when you move your body and feel your heart beat much faster.

• Examples: running, swimming fast, or playing sports like soccer or basketball.

Don't forget about your muscles!

• Do strength exercises at least 2 days a week.

• Examples: lifting weights, doing push-ups, or using resistance bands.

Remember:

• Exercise can make you feel good and help you stay healthy.

• Start slow and listen to your body.

***

**Should I do cardio or lift weights to prevent heart disease?**

**Answer this question using plain and easy-to-understand language in your response. This text about COVID-19 is a good example of the writing style (not content) you should use.**

**What is COVID-19?**

**• It is a new illness spreading around the world.**

**• It’s nickname is coronavirus.**

**How do you get it?**

**• Someone with COVID-19 gives you their germs.**

**• When they cough or sneeze, their germs get in the air, on you, and on things.**

**• Germs get into your body through your mouth, nose, and your eyes.**

***

Should you do cardio or lift weights to prevent heart disease?

What is heart disease?

• Heart disease affects your heart in a bad way.

• It can make your heart work poorly or stop working.

How can exercise help?

• Exercise keeps your heart strong and healthy.

• It can lower the chance of getting heart disease.

Which exercise is better?

• Cardio: This is when you do activities like running, biking, or swimming.

• Lifting weights: This is when you use weights or your body to build muscles.

What's the best choice?

• Both cardio and lifting weights can help your heart.

• It's a good idea to mix both exercises in your routine.

• Start slow and build up to keep it safe and fun.

***

Should you do cardio or lift weights to prevent heart disease?

What to do?

• Both cardio and weight lifting can help.

• Doing a mix of both is a good idea.

Why do cardio?

• Cardio makes your heart stronger.

• It helps your lungs work better.

• Examples: walking, running, swimming, and biking.

Why lift weights?

• It helps build muscle.

• Stronger muscles can make your heart work better.

• Examples: push-ups, squats, and using weights.

How often should you exercise?

• Aim for 150 minutes of exercise each week.

• You can split this into shorter sessions.

• Make sure to rest and not push too hard.

***

Should I do cardio or lift weights to prevent heart disease?

What is heart disease?

• It's when your heart has problems working right.

• It can make you feel tired, short of breath, or have chest pain.

How can exercise help?

• Exercise makes your heart stronger and healthier.

• It can lower your risk of heart disease.

Cardio or weights?

• Both are good for your heart.

• Cardio is like running, swimming, or dancing.

• Lifting weights is picking up heavy things and putting them down.

What should I do?

• Do both, if you can.

• Cardio helps your heart pump blood better.

• Lifting weights can lower blood pressure and help with cholesterol.

• Talk to a doctor or trainer to find the best plan for you.

***

**How can I lose weight?**

**Answer this question using plain and easy-to-understand language in your response. This text about COVID-19 is a good example of the writing style (not content) you should use.**

**What is COVID-19?**

**• It is a new illness spreading around the world.**

**• It’s nickname is coronavirus.**

**How do you get it?**

**• Someone with COVID-19 gives you their germs.**

**• When they cough or sneeze, their germs get in the air, on you, and on things.**

**• Germs get into your body through your mouth, nose, and your eyes.**

***

How can I lose weight?

• Eat healthier foods.

• Choose fruits, veggies, lean meats, and whole grains.

• Cut down on sugary drinks and snacks.

How can I exercise more?

• Find fun activities like walking, biking, or swimming.

• Try to exercise for 30 minutes a day, most days of the week.

• Invite a friend to join you for motivation.

***

How can you lose weight?

• Eat healthier food:

• Eat more fruits, veggies, and whole grains.

• Cut down on sugary, fatty, and processed foods.

• Control your portions:

• Eat smaller amounts of food.

• Use smaller plates and bowls to help.

• Be more active:

• Walk, run, swim, or dance – find what you like!

• Aim for 30 minutes of exercise most days.

• Drink water:

• Choose water instead of sugary drinks.

• Drink water before meals to help you feel full.

• Get enough sleep:

• Try to sleep 7-9 hours each night.

• Sleep helps your body work well and control hunger.

Remember, it's best to lose weight slowly and steadily. Talk to a doctor or dietitian for more help.

*******

How can I lose weight?

What is losing weight?

• It means making your body lighter by dropping extra fat.

• It helps you feel better and be healthier.

How can you lose weight?

1. Eat right:

• Enjoy more veggies and fruits.

• Choose lean meats like chicken or fish.

• Pick whole grains, like brown rice or whole wheat bread.

• Don't eat too much sugar or junk food.

2. Move more:

• Walk, jog, or dance to burn calories.

• Try fun activities like swimming or biking.

• Do it for at least 30 minutes a day.

3. Sleep well:

• Get enough rest, about 7-9 hours a night.

• Good sleep helps your body work better.

4. Drink water:

• Drink lots of water instead of sugary drinks.

• Water keeps you full and helps your body work well.

Remember, losing weight takes time. Be patient and keep trying. Talk to a doctor or a friend if you need help.

***

**How can I decrease LDL?**

**Answer this question using plain and easy-to-understand language in your response. This text about COVID-19 is a good example of the writing style (not content) you should use.**

**What is COVID-19?**

**• It is a new illness spreading around the world.**

**• It’s nickname is coronavirus.**

**How do you get it?**

**• Someone with COVID-19 gives you their germs.**

**• When they cough or sneeze, their germs get in the air, on you, and on things.**

**• Germs get into your body through your mouth, nose, and your eyes.**

***

How can I decrease LDL?

What is LDL?

• LDL is a type of fat in your blood.

• Its full name is low-density lipoprotein.

• Too much LDL is bad for you because it can clog your arteries.

How can you lower it?

1. Eat healthy foods:

• Choose fruits, vegetables, and whole grains.

• Pick lean meats, like chicken and fish, over red meat.

• Avoid foods high in saturated and trans fats, like fried foods and pastries.

2. Exercise more:

• Aim for 30 minutes of exercise most days.

• You can walk, run, swim, or dance – just get moving!

3. Lose weight if needed:

• Losing even a little weight can help lower LDL.

• Stick to a healthy diet and exercise to shed pounds.

4. Don't smoke:

• Smoking can raise LDL levels.

• If you smoke, try to quit.

5. Drink alcohol in moderation:

• Too much alcohol can raise LDL.

• Limit yourself to 1 drink a day for women, and 2 drinks a day for men.

Remember, always talk to your doctor about the best ways to lower your LDL for your health.

***

What is LDL?

• It is a type of cholesterol in your body.

• LDL stands for "low-density lipoprotein."

• Sometimes called "bad cholesterol."

Why should you decrease LDL?

• High LDL can lead to heart problems.

• Lower LDL helps keep your heart healthy.

How can you decrease LDL?

1. Eat better foods:

• Choose fruits, veggies, and whole grains.

• Eat less fatty meats and dairy.

• Try nuts, fish, and olive oil.

2. Exercise more:

• Aim for 30 minutes a day.

• Walking, jogging, or swimming are good options.

• Find an activity you enjoy.

3. Lose extra weight:

• Losing weight can help lower LDL.

• Eat smaller portions and avoid sugary drinks.

• Stay active and move more.

4. Don't smoke:

• Smoking can raise LDL levels.

• Quitting helps lower LDL and improve heart health.

5. Talk to a doctor:

• They can give you more advice.

• Sometimes, medicine is needed to lower LDL.

• A doctor can help decide what's best for you.

***

What is LDL?

• LDL stands for "low-density lipoprotein."

• It's a type of fat in your blood, often called "bad cholesterol."

Why should you decrease LDL?

• High LDL can lead to heart problems.

• It's important to keep LDL levels low to stay healthy.

How can you decrease LDL?

1. Eat healthier foods:

• Choose fruits, vegetables, and whole grains.

• Pick lean meats like chicken or fish.

• Limit fried foods and high-fat snacks.

2. Exercise more:

• Aim for at least 30 minutes of exercise most days.

• Walking, biking, and swimming are good options.

3. Lose weight if needed:

• Extra weight can raise LDL levels.

• Losing even a little weight can help lower LDL.

4. Stop smoking:

• Smoking can raise LDL levels.

• Quitting smoking is good for your heart and overall health.

5. Limit alcohol:

• Drink alcohol in moderation or not at all.

• Too much alcohol can raise LDL levels.

Remember to talk to your doctor for more advice on lowering LDL and staying healthy.

***

**How can I decrease triglycerides?**

**Answer this question using plain and easy-to-understand language in your response. This text about COVID-19 is a good example of the writing style (not content) you should use.**

**What is COVID-19?**

**• It is a new illness spreading around the world.**

**• It’s nickname is coronavirus.**

**How do you get it?**

**• Someone with COVID-19 gives you their germs.**

**• When they cough or sneeze, their germs get in the air, on you, and on things.**

**• Germs get into your body through your mouth, nose, and your eyes.**

***

What are triglycerides?

• They are a type of fat found in your blood.

• Your body uses them for energy.

Why lower triglycerides?

• High levels can be bad for your heart.

• Lower levels are better for your health.

How to decrease triglycerides?

1. Eat healthier:

• Choose whole foods, like fruits and vegetables.

• Pick lean meats, like chicken and fish.

• Eat less sugary and processed foods.

2. Exercise more:

• Aim for at least 30 minutes a day.

• Walking, biking, and swimming are good choices.

3. Lose weight:

• If you're overweight, try to lose some pounds.

• Small changes can make a big difference.

4. Limit alcohol:

• Drinking too much can raise triglycerides.

• Cut back or stop drinking alcohol.

5 Quit smoking:

• Smoking is bad for your heart.

• Quitting can help lower triglycerides.

Talk to a doctor for more advice on lowering triglycerides and improving your health.

***

How can I decrease triglycerides?

What are triglycerides?

• They are a type of fat found in your blood.

• Your body needs some, but too much is not good.

How can I lower them?

1. Eat healthy foods:

• Choose more fruits, vegetables, and whole grains.

• Pick lean proteins like fish and chicken.

• Limit foods high in sugar and unhealthy fats.

2. Exercise regularly:

• Aim for at least 30 minutes a day.

• Walking, jogging, and swimming are good options.

3. Lose extra weight:

• If you're overweight, losing a few pounds can help.

4. Limit alcohol:

• Drinking too much can raise triglyceride levels.

• Try to drink less or not at all.

5. Don't smoke:

• Smoking is bad for your heart and can raise triglycerides.

• If you smoke, try to quit.

Remember, always talk to your doctor for more advice and to make a plan that's right for you.

***

How can I decrease triglycerides?

What are triglycerides?

• They are a type of fat found in your blood.

• Your body uses them for energy.

Why should you lower them?

• High triglycerides can lead to health problems.

• It can cause heart issues and other troubles.

How can you lower them?

1. Eat healthier foods:

• Choose fruits, vegetables, and whole grains.

• Pick lean meats like chicken or fish.

• Avoid sugary and fatty foods.

2. Exercise more:

• Aim for 30 minutes a day, most days of the week.

• You can walk, swim, bike, or do other fun activities.

3. Lose weight:

• If you're overweight, losing some pounds can help.

• Make small changes, like eating smaller portions.

4. Don't smoke:

• If you smoke, try to quit.

• It's tough, but your doctor can help you find ways to stop.

5. Limit alcohol:

• Drink less alcohol or avoid it altogether.

• Too much alcohol can raise your triglycerides.

6. Talk to your doctor:

• They can give you more tips and check your progress.

• Sometimes, they might give you medicine to help lower triglycerides.

***

**What is lipoprotein(a)?**

**Answer this question using plain and easy-to-understand language in your response. This text about COVID-19 is a good example of the writing style (not content) you should use.**

**What is COVID-19?**

**• It is a new illness spreading around the world.**

**• It’s nickname is coronavirus.**

**How do you get it?**

**• Someone with COVID-19 gives you their germs.**

**• When they cough or sneeze, their germs get in the air, on you, and on things.**

**• Germs get into your body through your mouth, nose, and your eyes.**

***

What is lipoprotein(a)?

• It's a special type of fat in your blood.

• Some people call it Lp(a) for short.

Why is it important?

• High levels of Lp(a) can be bad for your heart.

• It can make it harder for blood to flow, leading to heart problems.

How do you know if you have it?

• A simple blood test can check your Lp(a) levels.

• Your doctor can tell you if it's too high and what to do about it.

***

What is lipoprotein(a)?

• It's a special kind of fat and protein combo in our blood.

• People often call it Lp(a) for short.

Why is it important?

• Some people have too much Lp(a), which can lead to heart problems.

• High levels of Lp(a) can make it harder for blood to flow smoothly.

How do we know if we have too much?

• A blood test can help check your Lp(a) levels.

• If it's high, your doctor can guide you on what to do next.

***

What is lipoprotein(a)?

• It is a particle found in our blood.

• It's also called Lp(a) for short.

Why is it important?

• High levels of Lp(a) can be bad for our health.

• It may cause heart and blood vessel problems.

How do we know if we have high Lp(a)?

• A blood test can measure your Lp(a) levels.

• Your doctor can help you understand the results.

***

**How can I quit smoking?**

**Answer this question using plain and easy-to-understand language in your response. This text about COVID-19 is a good example of the writing style (not content) you should use.**

**What is COVID-19?**

**• It is a new illness spreading around the world.**

**• It’s nickname is coronavirus.**

**How do you get it?**

**• Someone with COVID-19 gives you their germs.**

**• When they cough or sneeze, their germs get in the air, on you, and on things.**

**• Germs get into your body through your mouth, nose, and your eyes.**

*******

How can you quit smoking?

• Decide you want to stop.

• Pick a quit day soon.

• Get rid of cigarettes and lighters.

What can help you quit?

• Talk to a doctor or nurse.

• Use patches, gum, or medicine.

• Join a support group or class.

What else can you do?

• Avoid places where people smoke.

• Keep busy with other things.

• Drink water, chew gum, or eat snacks.

***

How can I quit smoking?

• Decide you want to stop.

• Pick a date to quit.

• Tell friends and family so they can help.

What can you do to quit?

• Stay away from things that make you want to smoke.

• Find other ways to relax or deal with stress.

• Keep your hands and mouth busy with gum or healthy snacks.

Where can you get help?

• Talk to a doctor or nurse about medicine or therapy.

• Join a support group or talk to a quit-smoking counselor.

• Use quit-smoking apps and websites for tips and help.

***

How can I quit smoking?

• Decide you want to stop.

• Set a date to quit smoking.

What can help you quit?

• Get support from friends and family.

• Talk to a doctor or counselor.

What tools can you use?

• Try nicotine gum or patches.

• Consider medicine to help you quit.

• Use apps or websites for quitting tips.

What else can help?

• Stay away from things that make you want to smoke.

• Keep your hands and mouth busy.

• Find new ways to relax and handle stress.

*******

**What are the side effects of statins?**

**Answer this question using plain and easy-to-understand language in your response. This text about COVID-19 is a good example of the writing style (not content) you should use.**

**What is COVID-19?**

**• It is a new illness spreading around the world.**

**• It’s nickname is coronavirus.**

**How do you get it?**

**• Someone with COVID-19 gives you their germs.**

**• When they cough or sneeze, their germs get in the air, on you, and on things.**

**• Germs get into your body through your mouth, nose, and your eyes.**

*******

What are statins?

• Statins are medicines that help lower bad cholesterol.

• They help keep your heart healthy.

What are the side effects?

• Some people might feel muscle pain or weakness.

• They could have stomach problems like nausea or diarrhea.

• Sometimes people feel dizzy or have a headache.

• A few people might get a rash or have trouble sleeping.

Remember, most people take statins without any problems. If you have side effects, talk to your doctor. They can help you find the best solution.

***

What are statins?

• Statins are medicines that help lower cholesterol.

• They are used to keep your heart healthy.

What are the side effects of statins?

• Some people might feel muscle pain or weakness.

• Others may have an upset stomach or feel more tired.

• A few people might get a headache or have trouble sleeping.

Remember:

• Not everyone gets side effects from statins.

• If you have side effects, talk to your doctor. They can help find the best solution for you.

*******

What are statins?

• They are medicines that help lower cholesterol.

• Doctors give them to people with high cholesterol.

What are the side effects of statins?

• Some people may feel muscle pain or weakness.

• Sometimes, people can have upset stomachs or feel dizzy.

• A few people may have headaches or feel more tired than usual.

Remember:

• Not everyone will have these side effects.

• If you feel worried or have questions, talk to your doctor. They will help you understand and can make changes if needed.

***

**I have muscle pain with a statin. What should I do?**

**Answer this question using plain and easy-to-understand language in your response. This text about COVID-19 is a good example of the writing style (not content) you should use.**

**What is COVID-19?**

**• It is a new illness spreading around the world.**

**• It’s nickname is coronavirus.**

**How do you get it?**

**• Someone with COVID-19 gives you their germs.**

**• When they cough or sneeze, their germs get in the air, on you, and on things.**

**• Germs get into your body through your mouth, nose, and your eyes.**

***

What to do when you have muscle pain with a statin?

• Statins are medicines that help lower bad cholesterol.

• Sometimes, they can cause muscle pain or soreness.

What should you do?

1. Talk to your doctor:

• Tell them about your muscle pain.

• They might change your medicine or dose.

2. Take care of yourself:

• Get enough rest.

• Drink lots of water.

• Eat healthy food.

3. Keep track of your pain:

• Write down when it hurts and how much.

• Share this with your doctor.

Remember, always talk to your doctor before making any changes to your medicines.

***

What to do if you have muscle pain with a statin?

• Statins are medicines for your heart.

• They help lower bad stuff in your blood called cholesterol.

What to do if you have muscle pain?

• Talk to your doctor or nurse.

• They will help you figure out what to do next.

• They might change your medicine or the amount you take.

Remember:

• Muscle pain is a common side effect of statins.

• But, it's important to tell your doctor or nurse so they can help you.

***

What to do with muscle pain from statins?

• Muscle pain is a side effect some people feel when they take statins.

• Statins are medicines to help lower bad cholesterol in your blood.

What should you do?

1. Talk to your doctor:

• Tell them about your muscle pain.

• They might change your medicine or dose.

2. Try home remedies:

• Rest, use a heating pad, or take a warm bath.

• Over-the-counter pain medicine might help too.

Remember, don't stop taking your medicine without talking to your doctor first.

***

**My cholesterol is still high and I'm already on a statin. What should I do?**

**Answer this question using plain and easy-to-understand language in your response. This text about COVID-19 is a good example of the writing style (not content) you should use.**

**What is COVID-19?**

**• It is a new illness spreading around the world.**

**• It’s nickname is coronavirus.**

**How do you get it?**

**• Someone with COVID-19 gives you their germs.**

**• When they cough or sneeze, their germs get in the air, on you, and on things.**

**• Germs get into your body through your mouth, nose, and your eyes.**

***

What to do when cholesterol is high and you're already on a statin?

• Talk to your doctor.

• Your doctor knows your health best. They can help you find the right solution.

• Eat healthier.

• Choose foods that are low in saturated fat, like fruits, veggies, whole grains, and lean meats.

• Exercise more.

• Try to be active for at least 30 minutes a day, most days of the week. Walking, swimming, and biking are good options.

• Lose weight.

• If you're overweight, losing even a few pounds can help lower cholesterol.

• Quit smoking.

• Smoking can raise bad cholesterol and lower good cholesterol. Quitting can help improve your numbers.

• Limit alcohol.

• Drinking too much can raise cholesterol. Stick to one drink a day for women and two drinks a day for men.

Remember, it's important to talk to your doctor before making any big changes to your lifestyle or medication.

***

What should I do if my cholesterol is high even though I'm on a statin?

• Talk to your doctor.

• They can help you understand why it's still high and if you need to make changes.

What else can I try?

• Eat healthier foods.

• Choose fruits, veggies, and whole grains.

• Limit fatty meats and full-fat dairy.

• Exercise more.

• Try to be active most days of the week.

• It can help your heart and lower cholesterol.

• Lose weight if needed.

• Extra weight can raise cholesterol.

• Losing even a little can help a lot.

• Stop smoking.

• Smoking is bad for your heart and can make cholesterol worse.

• Talk to your doctor if you need help quitting.

Remember, always talk to your doctor before making changes. They can help you find what's best for you.

***

What to do if cholesterol is high and you're taking a statin?

• Talk to your doctor.

• They know you best and can give you advice.

What else can you try?

• Eat healthier foods.

• Eat more fruits, veggies, and whole grains.

• Cut down on fatty foods, like fried stuff and butter.

• Get moving.

• Exercise more, like walking or swimming.

• Aim for 30 minutes a day, most days of the week.

• Lose extra weight.

• If you're heavy, losing weight can help.

• Work with your doctor to make a plan.

• Stop smoking.

• Smoking is bad for your heart.

• Ask your doctor for help to quit.

• Drink less alcohol.

• Too much alcohol can raise cholesterol.

• Have only a little or none at all.

Remember, always talk to your doctor first. They can help you find the best plan for you.

***

**What medications can reduce cholesterol other than statins?**

**Answer this question using plain and easy-to-understand language in your response. This text about COVID-19 is a good example of the writing style (not content) you should use.**

**What is COVID-19?**

**• It is a new illness spreading around the world.**

**• It’s nickname is coronavirus.**

**How do you get it?**

**• Someone with COVID-19 gives you their germs.**

**• When they cough or sneeze, their germs get in the air, on you, and on things.**

**• Germs get into your body through your mouth, nose, and your eyes.**

***

What are other medicines to lower cholesterol besides statins?

• There are different types of medicines to help lower cholesterol.

• Some common ones are bile acid binders, niacin, and fibric acid.

How do they work?

• Bile acid binders: These medicines help your body get rid of extra cholesterol.

• Niacin: This is a type of vitamin. It helps lower bad cholesterol and raise good cholesterol.

• Fibric acid: These drugs help lower fat in your blood and raise good cholesterol.

When do you need them?

• Sometimes, statins aren't enough, or they don't work well for you.

• Your doctor might suggest other medicines to help keep your cholesterol in check.

***

What are other medications to lower cholesterol?

• Cholesterol is a fat-like substance in your blood.

• Sometimes you have too much, and it can cause problems.

• There are other medications besides statins that can help.

Some examples are:

1. Bile acid sequestrants:

• These are like sponges that soak up cholesterol.

• They help your body get rid of cholesterol.

2. Niacin:

• This is a type of vitamin B.

• It can help lower bad cholesterol and raise good cholesterol.

3. Fibrates:

• These medicines help break down bad cholesterol.

• They can also help raise good cholesterol.

4. Cholesterol absorption inhibitors:

• These medicines stop your body from taking in cholesterol from food.

• They help lower bad cholesterol levels.

5. PCSK9 inhibitors:

• These are newer medicines.

• They help your liver break down bad cholesterol.

Remember, always talk to your doctor before starting any new medicine. They will help you choose the best one for you.

***

What are other medicines to lower cholesterol besides statins?

• There are different kinds to choose from.

• Some names are ezetimibe, bile acid binders, and niacin.

How do they work?

• Each kind has a special way to help.

• Ezetimibe stops your body from taking in cholesterol.

• Bile acid binders help remove cholesterol from your body.

• Niacin can lower bad cholesterol and raise good cholesterol.

Talk to a doctor to know which one is best for you.

***

**What is ezetimibe?**

**Answer this question using plain and easy-to-understand language in your response. This text about COVID-19 is a good example of the writing style (not content) you should use.**

**What is COVID-19?**

**• It is a new illness spreading around the world.**

**• It’s nickname is coronavirus.**

**How do you get it?**

**• Someone with COVID-19 gives you their germs.**

**• When they cough or sneeze, their germs get in the air, on you, and on things.**

**• Germs get into your body through your mouth, nose, and your eyes.**

***

What is ezetimibe?

• It is a medicine used to help lower bad cholesterol.

• It's also called Zetia.

How does it work?

• Ezetimibe stops your body from taking in too much bad cholesterol.

• This helps to lower the amount of bad cholesterol in your blood.

• When there's less bad cholesterol, your heart stays healthier.

***

What is ezetimibe?

• It is a medicine that helps lower bad cholesterol.

• Its nickname is Zetia.

How does it work?

• It stops your body from taking in bad cholesterol.

• When you eat food with cholesterol, ezetimibe blocks it from being absorbed.

• This helps to keep your heart healthy and lowers the risk of heart problems.

***

What is ezetimibe?

• It is a medicine used to lower cholesterol.

• It helps your body to not take in too much bad fat.

Why do we need it?

• High cholesterol can hurt your heart.

• It can lead to heart disease or a heart attack.

How do you use it?

• You take it as a pill, usually once a day.

• Your doctor will tell you the right dose for you.

***

**What are Repatha and Praluent?**

**Answer this question using plain and easy-to-understand language in your response. This text about COVID-19 is a good example of the writing style (not content) you should use.**

**What is COVID-19?**

**• It is a new illness spreading around the world.**

**• It’s nickname is coronavirus.**

**How do you get it?**

**• Someone with COVID-19 gives you their germs.**

**• When they cough or sneeze, their germs get in the air, on you, and on things.**

**• Germs get into your body through your mouth, nose, and your eyes.**

***

What are Repatha and Praluent?

• They are medicines to help lower bad cholesterol.

• Their real names are evolocumab (Repatha) and alirocumab (Praluent).

What do they do?

• They work in your body to stop a protein called PCSK9.

• This helps your liver remove more bad cholesterol from your blood.

• With less bad cholesterol, your risk of heart problems can go down.

***

What are Repatha and Praluent?

• They are medicines that help lower bad cholesterol.

• Their real names are evolocumab (Repatha) and alirocumab (Praluent).

What do they do?

• They help your liver remove bad cholesterol from your blood.

• This can lower your chance of having heart problems.

How do you use them?

• They are given as a shot, usually once or twice a month.

• A doctor or nurse will help you with the shots.

***

What are Repatha and Praluent?

• They are medicines that help lower bad cholesterol.

• Their big names are evolocumab (Repatha) and alirocumab (Praluent).

Why do people take them?

• Some people have too much bad cholesterol in their blood.

• When there's too much bad cholesterol, it can cause heart problems.

How do these medicines work?

• They work with your body to get rid of bad cholesterol.

• They help you have a healthier heart and blood vessels.

***

**What is inclisiran?**

**Answer this question using plain and easy-to-understand language in your response. This text about COVID-19 is a good example of the writing style (not content) you should use.**

**What is COVID-19?**

**• It is a new illness spreading around the world.**

**• It’s nickname is coronavirus.**

**How do you get it?**

**• Someone with COVID-19 gives you their germs.**

**• When they cough or sneeze, their germs get in the air, on you, and on things.**

**• Germs get into your body through your mouth, nose, and your eyes.**

*******

What is inclisiran?

• It is a new medicine for heart health.

• It helps lower bad cholesterol.

How does it work?

• Inclisiran stops a protein called PCSK9.

• This protein usually helps control cholesterol.

• By stopping PCSK9, inclisiran lets the body remove more bad cholesterol.

*******

What is inclisiran?

• It is a new medicine for people with high cholesterol.

• Its main goal is to lower bad cholesterol levels.

How does it work?

• Inclisiran helps your body remove bad cholesterol.

• It stops a special message that makes too much bad cholesterol.

• You take it as a shot, usually two times a year.

*******

What is inclisiran?

• It is a new medicine to help with cholesterol.

• It's also called Leqvio.

How does it work?

• Inclisiran helps lower bad cholesterol.

• It does this by stopping a protein in our body that makes bad cholesterol.

• With less bad cholesterol, our heart stays healthy.

***

**What are the side effects of Repatha and Praluent?**

**Answer this question using plain and easy-to-understand language in your response. This text about COVID-19 is a good example of the writing style (not content) you should use.**

**What is COVID-19?**

**• It is a new illness spreading around the world.**

**• It’s nickname is coronavirus.**

**How do you get it?**

**• Someone with COVID-19 gives you their germs.**

**• When they cough or sneeze, their germs get in the air, on you, and on things.**

**• Germs get into your body through your mouth, nose, and your eyes.**

***

What are Repatha and Praluent?

• They are medicines for treating high cholesterol.

• They help to lower bad cholesterol levels in your body.

What are the side effects of Repatha and Praluent?

• You may feel pain or redness where the shot was given.

• Some people might get a cold, sore throat, or the flu.

• Others could have a runny or stuffy nose.

• A few people may experience itching, rash, or swelling.

• In rare cases, some might feel muscle pain or weakness.

Remember, not everyone will have these side effects. If you're worried, talk to your doctor. They can help you understand more about the medicines and what to do if you have side effects.

***

What are Repatha and Praluent?

• They are medicines that help lower bad cholesterol.

• Doctors use them to help people with heart problems.

What are the side effects of Repatha and Praluent?

• Some people might have reactions at the spot where they get the shot, like redness, itching, or pain.

• Some people might get a stuffy nose or a sore throat.

• Others may have flu-like symptoms, like feeling tired or having a fever.

Remember, not everyone will have these side effects. If you have concerns, it's important to talk to your doctor. They can help you understand if these medicines are right for you.

***

What are Repatha and Praluent?

• They are medicines used to lower bad cholesterol.

• Their fancy names are "PCSK9 inhibitors."

What are some side effects of Repatha and Praluent?

• You might feel pain or redness where you got the shot.

• You could get a cold, sore throat, or the flu.

• Some people have back pain, feel very tired, or have an upset stomach.

Remember, not everyone gets these side effects. If you have questions or concerns, talk to your doctor. They can help you decide if these medicines are right for you.

***

**Should I take aspirin to prevent heart disease?**

**Answer this question using plain and easy-to-understand language in your response. This text about COVID-19 is a good example of the writing style (not content) you should use.**

**What is COVID-19?**

**• It is a new illness spreading around the world.**

**• It’s nickname is coronavirus.**

**How do you get it?**

**• Someone with COVID-19 gives you their germs.**

**• When they cough or sneeze, their germs get in the air, on you, and on things.**

**• Germs get into your body through your mouth, nose, and your eyes.**

***

Should you take aspirin to help your heart?

• Aspirin is a medicine that can help some people.

• It can lower the chance of heart problems.

When should you think about taking aspirin?

• If you're older, like over 40 or 50.

• If a doctor says you might have heart problems.

When should you not take aspirin?

• If you're young and healthy.

• If you have a problem with your stomach, like an ulcer.

What should you do?

• Talk to your doctor. They can help you decide.

• Don't just start taking aspirin on your own.

***

Should I take aspirin to prevent heart disease?

What is aspirin?

• It's a common medicine.

• People use it for pain, fever, and swelling.

Why use aspirin for heart disease?

• It can help make your blood thinner.

• Thinner blood can stop blood clots.

Should you take aspirin?

• Talk to your doctor first.

• They will check if it's good for you.

• It's not safe for everyone.

Remember:

• Always ask your doctor before starting aspirin.

• They know what's best for your health.

***

Should you take aspirin to prevent heart problems?

• Aspirin is a common medicine people use.

• It can help stop blood clots and lower heart attack chances.

Who should think about taking aspirin?

• Talk to your doctor first.

• They know what's best for you.

• Some people may need aspirin, others may not.

How do you take aspirin for your heart?

• Your doctor will tell you the right amount.

• Don't take more or less than they say.

• Always follow their advice.

Remember, talk to your doctor before starting any new medicine. They can help you make the best choice for your health.

***

**My cholesterol panel shows triglycerides 400 mg/dL. How should I interpret this?**

**Answer this question using plain and easy-to-understand language in your response. This text about COVID-19 is a good example of the writing style (not content) you should use.**

**What is COVID-19?**

**• It is a new illness spreading around the world.**

**• It’s nickname is coronavirus.**

**How do you get it?**

**• Someone with COVID-19 gives you their germs.**

**• When they cough or sneeze, their germs get in the air, on you, and on things.**

**• Germs get into your body through your mouth, nose, and your eyes.**

***

What are triglycerides?

• Triglycerides are a kind of fat found in your blood.

• They give your body energy.

• Your body needs some, but too many can be bad.

Your triglycerides are 400 mg/dL. What does this mean?

• It means you have high triglycerides.

• High triglycerides can be a problem.

• They can lead to heart issues and other health problems.

What can you do?

• Eat healthy foods like fruits, vegetables, and whole grains.

• Avoid sugary foods and drinks.

• Exercise and stay active.

• Talk to your doctor for more advice.

***

What are triglycerides?

• They are a type of fat found in your blood.

• Your body uses them for energy.

What does 400 mg/dL mean?

• It's a measure of triglycerides in your blood.

• 400 mg/dL is higher than normal.

Why is it important?

• High triglycerides can lead to health problems.

• They can cause heart issues and other diseases.

How can you lower triglycerides?

• Eat healthier foods like fruits, vegetables, and whole grains.

• Cut down on sugar, unhealthy fats, and alcohol.

• Exercise more and lose weight if needed.

• Your doctor might suggest medication too.

***

What does triglycerides 400 mg/dL mean?

• It means there is too much fat in your blood.

• Your level is high, which can be a problem.

Why is it a problem?

• High triglycerides can lead to heart issues.

• They can make your arteries narrow and harder.

How can you lower it?

• Eat healthier foods like fruits, vegetables, and whole grains.

• Avoid sugary drinks, fried foods, and too much alcohol.

• Exercise regularly, like walking or swimming.

• Your doctor might suggest medicine to help lower it.

Remember to talk to your doctor about your cholesterol levels and ask for advice on how to improve them.

***

**My LDL is 200 mg/dL. How should I interpret this?**

**Answer this question using plain and easy-to-understand language in your response. This text about COVID-19 is a good example of the writing style (not content) you should use.**

**What is COVID-19?**

**• It is a new illness spreading around the world.**

**• It’s nickname is coronavirus.**

**How do you get it?**

**• Someone with COVID-19 gives you their germs.**

**• When they cough or sneeze, their germs get in the air, on you, and on things.**

**• Germs get into your body through your mouth, nose, and your eyes.**

*******

What does LDL 200 mg/dL mean?

• LDL stands for "bad" cholesterol.

• Your number is 200 mg/dL, which is quite high.

Why is high LDL bad?

• Too much bad cholesterol can block blood flow.

• Blocked blood flow can cause heart problems.

What can you do?

• Eat healthier foods like fruits, veggies, and whole grains.

• Exercise regularly to stay active.

• Talk to your doctor for more help and advice.

***

What does an LDL of 200 mg/dL mean?

• It is a measure of the bad cholesterol in your blood.

• The number 200 is high, and that's not good.

Why should you care?

• Too much bad cholesterol can cause problems for your heart.

• It can make it harder for blood to flow and lead to heart disease.

What can you do?

• Talk to your doctor about your cholesterol levels.

• They might give you advice on how to eat healthier and exercise more.

• They could also suggest medicine to help lower your bad cholesterol.

***

What is LDL?

• LDL stands for "bad" cholesterol.

• It can build up in your blood vessels and cause problems.

What does 200 mg/dL mean?

• It means there are 200 milligrams of LDL cholesterol in every deciliter of your blood.

• This number is higher than what doctors usually want.

Is 200 mg/dL good or bad?

• It is not good. It's too high.

• A high LDL can lead to heart disease and other health problems.

What should you do?

• Talk to your doctor about your LDL.

• They may suggest changes in your diet, exercise, or medicines to help lower it.

Remember, always ask your doctor for advice. They know what's best for you.

***

**What does a coronary calcium score of 0 mean?**

**Answer this question using plain and easy-to-understand language in your response. This text about COVID-19 is a good example of the writing style (not content) you should use.**

**What is COVID-19?**

**• It is a new illness spreading around the world.**

**• It’s nickname is coronavirus.**

**How do you get it?**

**• Someone with COVID-19 gives you their germs.**

**• When they cough or sneeze, their germs get in the air, on you, and on things.**

**• Germs get into your body through your mouth, nose, and your eyes.**

***

What is a coronary calcium score of 0?

• It means your heart is very healthy.

• No calcium buildup is found in your heart's blood vessels.

Why is this important?

• Calcium buildup can make it hard for blood to flow.

• Good blood flow is needed for a strong heart.

• A score of 0 means a low chance of heart problems.

***

What is a coronary calcium score of 0?

• It is a test result for your heart.

• The score tells how much calcium is in your heart arteries.

What does a score of 0 mean?

• You have no calcium in your heart arteries.

• This is a good thing, it means your risk of heart problems is low.

How do you get a coronary calcium score?

• A special X-ray called a CT scan takes pictures of your heart.

• The doctor checks these pictures to see the calcium in your heart arteries.

***

What is a coronary calcium score of 0?

• It is a test result for your heart health.

• The score tells you if there's calcium in your heart arteries.

What does a score of 0 mean?

• It means you have no calcium in your heart arteries.

• This is good news! It shows a low risk for heart problems.

How is the score used?

• Doctors use it to check your heart's health.

• It helps them decide if you need more tests or treatments.

***

**What does a coronary calcium score of 100 mean?**

**Answer this question using plain and easy-to-understand language in your response. This text about COVID-19 is a good example of the writing style (not content) you should use.**

**What is COVID-19?**

**• It is a new illness spreading around the world.**

**• It’s nickname is coronavirus.**

**How do you get it?**

**• Someone with COVID-19 gives you their germs.**

**• When they cough or sneeze, their germs get in the air, on you, and on things.**

**• Germs get into your body through your mouth, nose, and your eyes.**

*******

What is a coronary calcium score of 100?

• It's a test result that tells you about your heart health.

• The score comes from a special X-ray called a CT scan.

What does a score of 100 mean?

• The score measures how much calcium is in your heart's arteries.

• A score of 100 means you have a moderate amount of calcium.

• It suggests a higher risk of heart problems compared to lower scores.

What should you do?

• Talk to your doctor about your score.

• They can help you make a plan to keep your heart healthy.

• This may include eating better, exercising, and taking medicine.

*******

What is a coronary calcium score of 100?

• It is a test result that checks your heart health.

• The number "100" tells us about the calcium in your heart arteries.

What does the number mean?

• A score of 100 means there is a medium amount of calcium in your arteries.

• This can lead to a higher risk of heart problems, but not as high as a larger number.

What should you do?

• Talk to your doctor about your score.

• They can help you make a plan to keep your heart healthy, like eating better and exercising more.

*******

What is a coronary calcium score of 100?

• It is a test result that measures the amount of calcium in your heart arteries.

• The number "100" tells you how much calcium is there.

What does it mean?

• A score of 100 means you have a medium amount of calcium in your heart arteries.

• This can be a sign that you have a higher chance of heart problems in the future.

How can you lower your risk?

• Eat healthy foods like fruits, vegetables, and whole grains.

• Exercise regularly to keep your heart strong.

• Don't smoke and avoid too much alcohol.

• Talk to your doctor about other ways to keep your heart healthy.

***

**What does a coronary calcium score of 400 mean?**

**Answer this question using plain and easy-to-understand language in your response. This text about COVID-19 is a good example of the writing style (not content) you should use.**

**What is COVID-19?**

**• It is a new illness spreading around the world.**

**• It’s nickname is coronavirus.**

**How do you get it?**

**• Someone with COVID-19 gives you their germs.**

**• When they cough or sneeze, their germs get in the air, on you, and on things.**

**• Germs get into your body through your mouth, nose, and your eyes.**

***

What is a coronary calcium score of 400?

• It is a test result that tells you about your heart health.

• The score shows how much calcium is in your heart arteries.

What does a score of 400 mean?

• A score of 400 means there is a lot of calcium in your heart arteries.

• This could be a sign that you have a higher chance of having a heart problem in the future.

How can you lower your risk?

• Eat healthier foods, like fruits, veggies, and whole grains.

• Exercise regularly, like going for a walk or a swim.

• Don't smoke, and if you do, try to quit.

• Talk to your doctor about other ways to lower your risk.

***

What is a coronary calcium score of 400?

• It is a test result that measures calcium in your heart arteries.

• The number 400 means there's a high amount of calcium.

Why does it matter?

• Calcium in the arteries can make them hard and narrow.

• This can lead to heart problems, like a heart attack.

What should you do?

• Talk to your doctor about your test result.

• They may suggest lifestyle changes or medicines to help your heart.

***

What is a coronary calcium score of 400?

• It is a test result that looks at your heart.

• The score tells you about calcium in your heart arteries.

What does the score mean?

• A score of 400 means you have a good amount of calcium in your heart arteries.

• This can be a sign that you might have a higher risk of heart problems.

Why is this important?

• Knowing your score helps you and your doctor make a plan to keep your heart healthy.

• You might need to eat better, exercise more, or take medicine to lower your risk of heart problems.

***

**What genetic mutations can cause high cholesterol?**

**Answer this question using plain and easy-to-understand language in your response. This text about COVID-19 is a good example of the writing style (not content) you should use.**

**What is COVID-19?**

**• It is a new illness spreading around the world.**

**• It’s nickname is coronavirus.**

**How do you get it?**

**• Someone with COVID-19 gives you their germs.**

**• When they cough or sneeze, their germs get in the air, on you, and on things.**

**• Germs get into your body through your mouth, nose, and your eyes.**

*******

What are genetic mutations causing high cholesterol?

• They are changes in your DNA that make cholesterol levels go up.

• Cholesterol is a fat-like substance in your blood.

Which genetic mutations cause it?

• Familial Hypercholesterolemia (FH) is one example.

• It happens when a gene called LDLR, APOB, or PCSK9 doesn't work right.

How do these mutations affect you?

• Your body can't remove bad cholesterol (LDL) well.

• Too much bad cholesterol can cause heart problems.

***

What are genetic mutations causing high cholesterol?

• They are changes in your genes that make cholesterol levels go up.

Which gene changes are linked to high cholesterol?

• Two common ones are called FH and FDB.

• FH stands for Familial Hypercholesterolemia.

• FDB stands for Familial Defective ApoB-100.

How do these gene changes cause high cholesterol?

• FH makes it hard for your body to remove bad cholesterol.

• FDB makes it tough for your body to use good cholesterol.

Why is high cholesterol a problem?

• It can lead to heart issues and other health problems.

• It's important to know if you have high cholesterol and talk to a doctor.

***

What is high cholesterol?

• It's when you have too much fat-like stuff in your blood.

• It can lead to heart problems.

What causes high cholesterol from genes?

• Sometimes it's because of changes in your DNA, called mutations.

• These mutations can make your body not handle cholesterol well.

Some common gene mutations causing high cholesterol are:

FH mutation:

• Stands for Familial Hypercholesterolemia.

• Makes your body bad at removing bad cholesterol (LDL).

• Can lead to very high cholesterol levels.

APOE mutation:

• Affects a gene called Apolipoprotein E.

• This gene helps control how your body uses and removes fats.

• Different changes in APOE can raise or lower cholesterol levels.

Remember, not all high cholesterol is from genes. Diet, exercise, and other factors play a big role too. Talk to a doctor if you're worried about your cholesterol levels.

**Plain Language (Rubric)**

**How can I prevent heart disease?**

**Answer this question using plain and easy-to-understand language in your response. Your response should mention diet, exercise, and risk factor control and should also recommend talking with a healthcare professional.**

***

Sure, I'd be happy to give some advice on how to help prevent heart disease!

1. Eat a healthy diet: Try to include lots of fruits, vegetables, whole grains, and lean proteins like fish and chicken in your meals. Limit foods high in saturated fats, trans fats, and sodium. This can help lower your cholesterol levels and blood pressure, which are both important for heart health.

2. Exercise regularly: Regular physical activity is super good for your heart. Aim for at least 30 minutes of moderate exercise most days of the week. That can be anything that gets you moving, like walking, biking, or dancing.

3. Control your risk factors: If you smoke, try to quit. If you have high blood pressure or diabetes, work with your healthcare provider to manage it. Both smoking and uncontrolled health conditions can increase your risk of heart disease.

4. Maintain a healthy weight: Being overweight can put extra stress on your heart. So, eating a balanced diet and exercising regularly can help you maintain a healthy weight.

5. Limit alcohol: Too much alcohol can raise your blood pressure and add extra calories, which might lead to weight gain. Try to limit your drinking to a moderate amount.

Remember, it's always important to talk with a healthcare professional about your health. They can provide you with information specific to your needs and help you make a plan that's right for you.

These are just general guidelines. Everyone's body and health situation is different, so what works best for you might not be the same as what works best for someone else. But these tips are a good place to start for most people!

***

Sure, let's talk about how you can protect your heart in a way that's easy to understand:

1. Eat a Heart-Healthy Diet: Choose foods that are good for your heart. This means lots of fruits, vegetables, whole grains, and lean proteins like chicken or fish. Try to limit things like red meat, sugary foods, and salty snacks. Also, avoid unhealthy fats like trans fat and saturated fat, often found in fried or processed foods.

2. Get Moving: Regular physical activity is very important. Try to get at least 30 minutes of exercise most days of the week. You don't have to run a marathon - even walking, biking, or dancing counts. Find an activity that you enjoy and it'll be easier to stick with it.

3. Stay at a Healthy Weight: If you're overweight, losing even a few pounds can help your heart. Talk to your doctor about what a healthy weight is for you, and how to reach it.

4. Don't Smoke: If you smoke, try to quit. Smoking is really bad for your heart. There are many resources available to help you quit.

5. Limit Alcohol: Drinking too much alcohol can raise your blood pressure and add extra calories, which can lead to weight gain. Try to limit how much you drink.

6. Control Your Blood Pressure: High blood pressure is a major risk factor for heart disease. Regular check-ups with your doctor, a healthy diet, and regular exercise can help keep your blood pressure in check.

7. Manage Your Stress: Too much stress isn't good for your heart. Find ways to relax and unwind, like meditation, yoga, reading, or spending time in nature.

Remember, the best way to know what's right for you is to talk with a healthcare professional. They can give you personalized advice based on your health history and current situation. Prevention is the best medicine, so start taking steps today to keep your heart healthy.

***

Sure, I can help with that! To keep your heart healthy, here's what you can do:

1. Eat Smart: Have lots of fruits, veggies, whole grains, lean meats like chicken or fish, and low-fat dairy products. Try to limit foods high in salt, sugar, and unhealthy fats like trans fat and saturated fat. This means less fast food and more home-cooked meals. It's like putting good quality fuel in a car.

2. Move More: Aim for at least 30 minutes of exercise most days. It can be as simple as a brisk walk, a bike ride, or even dancing. You don't need to run a marathon. Just get your body moving to help keep your heart in shape.

3. Stay Away from Smoking: Smoking is very bad for your heart. If you smoke, try to quit. And if you don't smoke, don't start.

4. Limit Alcohol: Too much alcohol can hurt your heart. Try to have no more than one drink a day for women and two for men.

5. Watch Your Weight: Being overweight can strain your heart. Aim to keep your weight in a healthy range. Your doctor can help figure out what that range is for you.

6. Manage Stress: Too much stress isn't good for your heart. Try activities that help you relax like yoga, meditation, or even just reading a book.

7. Control Risk Factors: If you have high blood pressure, high cholesterol, or diabetes, work with your healthcare provider to manage these conditions. This might involve taking medicine and making lifestyle changes.

Remember, it's always a good idea to talk to a healthcare professional like a doctor or a nurse. They can give you advice that's just right for you. Just like how every car needs a good mechanic, every heart needs a good doctor! They can help you know more about your heart health and what you can do to improve it.

***

**What is the best diet for the heart?**

**Answer this question using plain and easy-to-understand language in your response. Your response should mention the following points: 1) eating more fruits and vegetables, 2) avoiding refined carbohydrates, processed foods, and added sugar, 3) limiting unhealthy fats and red meat, and 4) reducing excess sodium intake. Alternatively, your response can discuss a specific diet recommended by the American Heart Association.**

***

To keep your heart happy and healthy, you need to eat the right stuff. Here are the key things you need to know:

1) Love fruits and vegetables: Make fruits and veggies your best friends. They have a lot of good stuff like vitamins, minerals, and fiber, which your heart loves. Try to eat a variety of them to get the full range of nutrients.

2) Say no to refined carbs, processed foods, and added sugar: Foods like white bread, soda, and candy don't do your heart any favors. They can make you gain weight and can lead to heart disease. So, try to limit them or avoid them if possible.

3) Be smart about fats and meat: Some fats are okay, but others aren't. Try to limit fats like butter and the fat you find in red meat. Speaking of meat, try not to eat too much red meat, like beef or pork. It's better to eat lean meats like chicken or fish, or even plant-based proteins like beans or tofu.

4) Hold the salt: Too much salt (sodium) can raise your blood pressure, which isn't good for your heart. So, try to limit your intake. Watch out for packaged foods, they often have a lot of hidden salt.

Remember, it's not just about one meal, it's about your whole pattern of eating. The American Heart Association recommends the DASH diet (Dietary Approaches to Stop Hypertension), which focuses on all these points. It's a well-balanced diet with lots of fruits, veggies, whole grains, lean proteins, and low-fat dairy, and less of the salty, fatty, and sugary stuff.

In simple words, try to eat more of the good stuff (fruits, veggies, lean proteins), and less of the not-so-good stuff (refined carbs, processed foods, unhealthy fats, too much salt).

***

Sure, I'd be happy to help explain this. So, if you want to take care of your heart, what you eat really matters. Here are some important points to keep in mind:

1) Eating more fruits and vegetables: Fruits and veggies are like the superstars of heart-healthy foods. They're packed full of things that are really good for your heart, like vitamins, minerals, and fiber. Plus, they can help you feel full without a lot of calories, which can help you maintain a healthy weight.

2) Avoiding refined carbohydrates, processed foods, and added sugar: Refined carbs are like the villains of heart health. They're found in things like white bread and sugary drinks. They can cause your blood sugar to spike and may lead to weight gain and other heart problems. Same goes for processed foods and added sugars - they're just not good for your heart. Try to stick with whole grains instead.

3) Limiting unhealthy fats and red meat: Not all fats are bad, but some are worse for your heart than others. Try to limit things like saturated and trans fats, which you find in foods like butter, cheese, and red meats. They can raise your "bad" cholesterol levels. Instead, opt for healthier fats found in foods like avocados, nuts, and fish.

4) Reducing excess sodium intake: While a little bit of sodium is okay, too much can raise your blood pressure, which isn't good for your heart. Try to cut back on high-sodium foods like processed snacks, fast food, and canned soups.

Now, if you're looking for a specific diet that's good for your heart, the American Heart Association recommends the DASH diet (Dietary Approaches to Stop Hypertension). It's all about eating plenty of fruits, veggies, whole grains, and lean proteins, and limiting sodium, sugars, and unhealthy fats. It's a great way to help keep your heart happy and healthy.

***

Taking care of your heart can be as simple as making smart choices about what you put on your plate. Here are some ways you can eat your way to a healthier heart:

1) Eat More Fruits and Veggies: These guys are like superheroes for your heart! They're loaded with vitamins, minerals, and fiber but are low in calories. They help keep your heart happy by helping you maintain a healthy weight and lowering your risk of heart disease.

2) Avoid Refined Carbs, Processed Foods, and Added Sugars: Foods like white bread, cookies, and sugary drinks may taste good, but they aren't great for your heart. They can cause your blood sugar to spike and then crash, which can lead to heart issues over time. Instead, try choosing whole grains and natural sugars found in fruits.

3) Limit Unhealthy Fats and Red Meat: Not all fats are bad, but some, like saturated and trans fats found in things like red meat and fried foods, can raise your cholesterol and risk of heart disease. Instead, try to get your fats from healthier sources like fish, nuts, and avocados.

4) Reduce Excess Sodium Intake: Too much salt can lead to high blood pressure, a big risk factor for heart disease. Try to limit how much you use and be mindful of hidden sodium in processed foods and restaurants.

The American Heart Association recommends a diet that follows these guidelines, such as the DASH diet (Dietary Approaches to Stop Hypertension). This diet emphasizes all the points above and includes plenty of whole grains, lean proteins, and low-fat dairy.

Remember, the best diet is one you can stick to, so find ways to incorporate these healthy habits into meals you enjoy! And, as always, it's a good idea to talk to your doctor or a dietitian before starting any new diet plan.

***

**What is the best diet for high blood pressure and high cholesterol?**

**Answer this question using plain and easy-to-understand language in your response. Your response should mention the following points: 1) eating more fruits and vegetables, 2) avoiding refined carbohydrates, processed foods, and added sugar, 3) limiting unhealthy fats and red meat, and 4) reducing excess sodium intake. Alternatively, your response can discuss a specific diet recommended by the American Heart Association.**

***

Sure, I'd be happy to explain this. When you've got high blood pressure and high cholesterol, what you eat can make a big difference. Here are some simple changes you can make:

1. Eat more fruits and vegetables: They're good for you because they're full of things called nutrients and fiber, but don't have a lot of calories. This helps you stay healthy and can also help you keep your weight down.

2. Avoid refined carbohydrates, processed foods, and added sugar: These are often found in things like white bread, soda, and snacks. They can make your blood sugar go up and down quickly, and are also often high in unhealthy fats and sodium.

3. Limit unhealthy fats and red meat: These are found in things like butter, cheese, and beef. Eating too much of these can raise your cholesterol levels. Instead, try to eat more lean meats like chicken or fish, or plant-based proteins like beans or tofu.

4. Reduce excess sodium intake: Sodium is a fancy word for salt. Eating too much salt can raise your blood pressure. Try to cook at home more often, as restaurant and processed foods often have a lot of salt.

One specific diet that the American Heart Association recommends is called the DASH diet, which stands for "Dietary Approaches to Stop Hypertension." It has all of these ideas in it, plus it tells you to eat lots of whole grains and low-fat dairy products, and to limit alcohol.

But remember, everyone's body is different, so what works best for you might not work best for someone else. It's always a good idea to talk to your doctor or a dietitian before you make big changes to what you eat. They can give you more personalized advice.

***

Sure, I'll keep this simple and straightforward for you!

If you have high blood pressure and high cholesterol, your diet is a great place to start in order to improve these conditions. Here's what you need to do:

1) Eat more fruits and veggies: Fruits and vegetables are powerhouses of nutrition. They are low in calories, rich in dietary fiber, and packed with heart-healthy nutrients. Think of adding more spinach to your meals, munching on carrots as snacks, or adding slices of fruit to your breakfast.

2) Avoid refined carbs, processed foods, and added sugars: Foods like white bread, candy, soda, and chips can cause your blood pressure and cholesterol levels to go up. So, instead of these, try to eat whole grains like brown rice and whole grain bread, and natural sugars from fruits.

3) Limit unhealthy fats and red meat: Saturated and trans fats found in foods like red meat can increase your cholesterol levels. Try to eat lean proteins like chicken, fish, or plant-based proteins like lentils and beans more often. And when you use fats, opt for healthier ones like olive oil.

4) Reduce excess sodium intake: Eating too much salt can increase your blood pressure. So, be mindful of the amount of salt you use in your cooking, and try to limit high-sodium foods like canned soups and fast food.

You might also want to look into the DASH (Dietary Approaches to Stop Hypertension) diet. This is a diet specifically recommended by the American Heart Association. It focuses on all the points above and encourages eating lots of whole grains, fruits, vegetables, and lean proteins, while limiting salt, sugar, and unhealthy fats.

Remember, making changes to your diet can be a big step, so it's okay to start small and make one change at a time. And, it's always a good idea to talk to a healthcare provider or a dietitian before making any major changes to your diet.

***

Sure, I'd be happy to explain this. When you've got high blood pressure and high cholesterol, what you eat can make a big difference. Here are some simple changes you can make:

1. Eat more fruits and vegetables: They're good for you because they're full of things called nutrients and fiber, but don't have a lot of calories. This helps you stay healthy and can also help you keep your weight down.

2. Avoid refined carbohydrates, processed foods, and added sugar: These are often found in things like white bread, soda, and snacks. They can make your blood sugar go up and down quickly, and are also often high in unhealthy fats and sodium.

3. Limit unhealthy fats and red meat: These are found in things like butter, cheese, and beef. Eating too much of these can raise your cholesterol levels. Instead, try to eat more lean meats like chicken or fish, or plant-based proteins like beans or tofu.

4. Reduce excess sodium intake: Sodium is a fancy word for salt. Eating too much salt can raise your blood pressure. Try to cook at home more often, as restaurant and processed foods often have a lot of salt.

One specific diet that the American Heart Association recommends is called the DASH diet, which stands for "Dietary Approaches to Stop Hypertension." It has all of these ideas in it, plus it tells you to eat lots of whole grains and low-fat dairy products, and to limit alcohol.

But remember, everyone's body is different, so what works best for you might not work best for someone else. It's always a good idea to talk to your doctor or a dietitian before you make big changes to what you eat. They can give you more personalized advice.

***

**How much should I exercise to stay healthy?**

**Answer this question using plain and easy-to-understand language in your response. Your response should suggest 150 minutes of moderate intensity or 75 minutes of vigorous intensity exercise per week and should also recommend talking with a healthcare professional.**

***

Sure, let's break it down simply.

If you want to stay healthy, you should try to aim for a certain amount of exercise every week. This could mean doing activities that are "moderate intensity" for 150 minutes. What's "moderate intensity"? This could be something like a brisk walk, where you're moving quicker than a leisurely stroll, but you're not out of breath.

If you prefer more intense workouts, then 75 minutes of "vigorous intensity" exercise could be your target. This is when you're really pushing yourself, like running or playing a high-energy sport.

Remember, these minutes don't have to be done all at once. You can break them down into smaller chunks, like 30 minutes a day for five days a week.

However, before starting any new exercise program, it's always a good idea to chat with a healthcare professional. They can give you guidance that's tailored specifically to you, your health, and your lifestyle.

***

Sure, I can make it simple. You know how there are 60 minutes in an hour, right? Well, to keep your body in tip-top shape, you need to be moving and getting your heart pumping for about 150 minutes each week if you're doing stuff that's not too hard, like a brisk walk. But, if you're doing something tougher that really gets you sweaty and breathless, like running or intense aerobics, then you only need about 75 minutes a week.

So, think of it like this. If you choose the easier exercises, it's like spending a bit over 2 hours in a week, which is not much if you spread it over a few days. And if you pick the tougher ones, it's just a bit over an hour. Both ways are great to keep you healthy and feeling good.

But remember, everyone's different, and what works best for one person might not work as well for another. It's always a good idea to have a chat with your doctor or another health expert before starting any new exercise routine. They can help make sure the exercise you choose is safe for you and suits your body's needs.

***

Alright, let's keep this simple!

For a healthy lifestyle, experts suggest that you need about 150 minutes (that's 2 and a half hours) of moderate-intensity exercise every week. That might sound like a lot, but it can be as easy as going for a brisk 30-minute walk, 5 days a week.

On the other hand, if you're into more intense stuff like jogging or playing sports, then you only need about 75 minutes each week. That's just a little over an hour. This type of workout makes your heart beat faster and makes you breathe harder than normal.

But remember, everyone's different, so what works best for one person might not be the best for another. It's always a good idea to chat with a healthcare professional before starting a new exercise routine to make sure it's safe and right for you.

Also, don't forget to do exercises that strengthen your muscles at least twice a week. These can include lifting weights, doing push-ups, or yoga.

Just keep moving and make sure you're enjoying yourself! Exercise is much easier when it's something fun. Happy exercising!

***

**Should I do cardio or lift weights to prevent heart disease?**

**Answer this question using plain and easy-to-understand language in your response. Your response should specify that the correct answer to this question depends on the individual and should recommend talking with a healthcare professional or exercise physiologist.**

***

Sure, happy to explain this in simple terms!

Heart disease is a common health problem, and regular physical activity is a fantastic way to help prevent it. But the best type of exercise, cardio or lifting weights, really depends on you.

Cardio exercise, like running, biking, or swimming, is a great way to keep your heart strong and healthy. It helps to lower blood pressure and cholesterol levels, which can reduce the risk of heart disease.

Weight lifting, on the other hand, also has benefits. It helps to strengthen your muscles, improve your balance, and can even boost your mood. Some research also suggests that weight lifting could help with heart health, though we generally think of cardio as being more directly connected to heart health.

Now, here's the key point: the best exercise for you depends on your own body, your current health, your goals, and what you enjoy doing. Some people might benefit more from cardio, while others might do better with weight lifting. Or a combination of both might be best! So, it's really a personal thing.

I strongly recommend that you talk to a healthcare professional or exercise physiologist about this. They can help you figure out what type of exercise is best for your individual circumstances, taking into account your current fitness level, health status, age, and other factors. They can guide you in creating a safe and effective exercise plan.

Remember, the most important thing is to stay active and make exercise a part of your regular routine. No matter what type of exercise you choose, it's a great step towards keeping your heart healthy!

***

Sure, I'll break it down in simple terms. Both cardio exercises and lifting weights can be helpful in preventing heart disease. Here's why:

1. Cardio exercises, like running, swimming or cycling, increase your heart rate and make your heart stronger over time. They also help to reduce blood pressure and cholesterol, which are both factors in heart disease.

2. Lifting weights, on the other hand, can also benefit your heart. It helps you build muscle, which burns more calories even when you're not working out. This can help you maintain a healthy weight, which is good for your heart. Plus, it can help control blood sugar levels, reducing the risk of type 2 diabetes, another risk factor for heart disease.

But here's the important bit - it really depends on you as an individual. Your current health status, any existing health conditions, and even your personal likes and dislikes, all matter. Some people might benefit more from cardio, while others from weight lifting, and a balanced mix of both could be the best for many.

To find the best approach for you, it's a good idea to have a chat with a healthcare professional or an exercise physiologist. They can help tailor an exercise routine that suits you and your heart the best!

***

Sure, here's a simple explanation.

Both cardio exercises (like jogging or cycling) and weightlifting can help your heart stay healthy.

Cardio gets your heart rate up and improves your endurance. It can help to lower your blood pressure and cholesterol, which can reduce your risk of heart disease.

Weightlifting, on the other hand, builds muscle and can also be good for your heart. It can help lower body fat, improve your metabolism, and even help control blood sugar levels.

So, both types of exercise have their benefits. But, which one is better for you can depend on a lot of things like your age, current fitness level, or any health conditions you might have.

That's why it's a good idea to chat with a healthcare professional or exercise physiologist. They can look at your personal situation and help you make the best decision.

Remember, any exercise is better than none. And a combo of both cardio and weights can give you the benefits of both. The key is to start slow, listen to your body, and gradually increase your activity level as your fitness improves.

So go ahead, take that first step, and don't forget to enjoy your exercise routine!

***

**How can I lose weight?**

**Answer this question using plain and easy-to-understand language in your response. Your response should mention diet and exercise and should also recommend talking with a healthcare professional.**

***

Absolutely, I can break it down into simple terms.

1. Eat Better: Aim for balanced meals with lean proteins, fruits, vegetables, and whole grains. Cut back on sugar, processed foods, and drinks with lots of calories like soda. It's about eating less but also eating smarter.

2. Move More: Get some form of exercise each day. Walking, swimming, biking, or dancing can all be good choices. Aim for at least 30 minutes per day, but even a little bit helps.

3. Talk to a Pro: A healthcare professional, like a doctor or a dietitian, can give you advice that's personalized for you. They can consider your medical history and lifestyle to help you make safe and effective choices.

4. Patience is Key: Losing weight is often a slow process. It's about changing habits and sticking to them. Even small changes can add up over time.

Remember, the best plan is the one you can stick with. What works for one person may not work for another. It's about finding healthy habits that you enjoy and can keep up for the long run.

***

Sure, here are some straightforward tips to help you lose weight:

1. Eat Healthily: Try to fill your plate with more fruits, vegetables, whole grains, and lean proteins. These foods can make you feel full without many calories. It's also a good idea to reduce intake of sugar, salt, and unhealthy fats found in foods like fast food and sugary drinks.

2. Regular Exercise: Aim to get moving every day. This could be anything from a brisk walk, a dance class, or playing a sport. You don't have to go to the gym, just find something you enjoy that gets you moving. It's suggested to get at least 150 minutes of moderate aerobic activity or 75 minutes of vigorous activity per week.

3. Control Portion Sizes: Pay attention to how much you're eating at one time. This doesn't mean you need to measure and count everything, but use your judgement to make sure your portions are reasonable.

4. Stay Hydrated: Drinking enough water can help control hunger and keep you hydrated.

5. Get Enough Sleep: Lack of sleep can mess with your metabolism and can make it harder to lose weight.

6. Regular Check-ins: Try to weigh yourself at least once a week to keep track of your progress.

Remember, losing weight is different for everyone and what works for one person might not work for another. It's important to talk with a healthcare professional like a doctor or a dietitian before starting any weight loss plan. They can provide advice tailored to your personal health needs and goals.

***

Sure, I can explain this in simple terms. Losing weight often involves a combination of changing your eating habits and becoming more active.

1. Diet: It's important to eat a balanced diet. This means having meals that include proteins (like fish, chicken, beans), carbohydrates (like rice, bread, pasta), fruits, veggies, and a small amount of fats. Try to cut down on foods and drinks that are high in sugar, like sodas and candy, and also limit fast food. Eating smaller portions can also help.

2. Exercise: Moving your body is a key part of losing weight. Aim for around 30 minutes of exercise a day if you can. This could be as simple as a brisk walk, cycling, or even dancing to your favorite songs. You don't need to run a marathon or lift heavy weights to get started!

3. Speak to a healthcare professional: This is very important. Before starting any new diet or exercise routine, it's always best to chat with a doctor or a nutritionist. They can make sure the changes you're planning to make are safe and right for your body.

Remember, everyone's body is different, so what works for one person may not work for another. And losing weight takes time, so try to be patient with yourself. Changes won't happen overnight, but with consistency and determination, you'll see progress.

***

**How can I decrease LDL?**

**Answer this question using plain and easy-to-understand language in your response. Your response should mention lifestyle modifications including diet and exercise and medications such as statins; it should also recommend talking with a healthcare professional.**

***

Sure, I can definitely help with that! LDL, or "bad" cholesterol, can be lowered in a few ways. Here's how:

1. Diet: Eat foods that are good for your heart, like fruits, vegetables, whole grains, and lean proteins. Try to avoid foods high in saturated fats, like red meat and full-fat dairy products, because these can raise your LDL levels. Also, try to limit your intake of foods high in cholesterol and trans fats.

2. Exercise: Regular physical activity can help lower your LDL levels. Aim for at least 30 minutes of exercise most days of the week. This can include things like walking, biking, or swimming.

3. Weight: If you're overweight, losing even a small amount of weight can help reduce your LDL levels. Even a small weight loss can make a big difference.

4. Don't smoke: Smoking can increase your LDL levels. If you smoke, consider quitting. There are many resources available to help you quit.

5. Limit alcohol: Drinking alcohol in moderation or not at all can also help lower your LDL levels.

6. Medications: If lifestyle changes aren't enough, your doctor might recommend medication to lower your LDL. These could include drugs called statins, which help lower LDL levels in your body.

Remember, it's always a good idea to talk with your doctor or another healthcare professional before starting any new diet or exercise program, or before starting any new medication. They can provide you with advice that's tailored to your specific needs and health condition.

***

Sure, here are some simple ways to decrease your LDL (bad) cholesterol levels:

1. Eat heart-healthy foods: Cut back on saturated fats found in red meat and full-fat dairy products and trans fats found in many processed foods. Include more foods high in omega-3 fatty acids like fish, walnuts, and flaxseeds. Add more soluble fiber to your diet (found in oats, fruits, beans, lentils) and eat more fruits and vegetables.

2. Exercise: Aim to be physically active for at least 30 minutes a day. This could be brisk walking, swimming, cycling, or any exercise you enjoy. Regular exercise can help raise your HDL (good) cholesterol and lower your LDL cholesterol.

3. Lose excess weight: If you're overweight, losing even a few pounds can help lower LDL.

4. Don't smoke and limit alcohol: Smoking lowers your good cholesterol, increases your bad cholesterol, and damages your blood vessels. Similarly, too much alcohol can raise cholesterol levels.

5. Manage stress: High levels of stress over a long time can raise your cholesterol levels. Try stress-reducing activities like yoga, meditation, and spending time with loved ones.

6. Medications: If lifestyle changes aren't enough, your doctor may prescribe medications to help lower your LDL. One common type of medication is statins.

Remember, everyone is different, and what works best for you will depend on your overall health, lifestyle, and genetics. Always consult with a healthcare professional before making significant changes to your diet or exercise routine, or starting new medications. They can give you personalized advice and monitor your progress.

***

Sure, here are some simple ways to decrease your LDL (bad) cholesterol levels:

1. Eat heart-healthy foods: Cut back on saturated fats found in red meat and full-fat dairy products and trans fats found in many processed foods. Include more foods high in omega-3 fatty acids like fish, walnuts, and flaxseeds. Add more soluble fiber to your diet (found in oats, fruits, beans, lentils) and eat more fruits and vegetables.

2. Exercise: Aim to be physically active for at least 30 minutes a day. This could be brisk walking, swimming, cycling, or any exercise you enjoy. Regular exercise can help raise your HDL (good) cholesterol and lower your LDL cholesterol.

3. Lose excess weight: If you're overweight, losing even a few pounds can help lower LDL.

4. Don't smoke and limit alcohol: Smoking lowers your good cholesterol, increases your bad cholesterol, and damages your blood vessels. Similarly, too much alcohol can raise cholesterol levels.

5. Manage stress: High levels of stress over a long time can raise your cholesterol levels. Try stress-reducing activities like yoga, meditation, and spending time with loved ones.

6. Medications: If lifestyle changes aren't enough, your doctor may prescribe medications to help lower your LDL. One common type of medication is statins.

Remember, everyone is different, and what works best for you will depend on your overall health, lifestyle, and genetics. Always consult with a healthcare professional before making significant changes to your diet or exercise routine, or starting new medications. They can give you personalized advice and monitor your progress.

***

**How can I decrease triglycerides?**

**Answer this question using plain and easy-to-understand language in your response. Your response should mention lifestyle modifications including diet and exercise, limiting alcohol use, and losing weight; it should also recommend talking with a healthcare professional.**

***

Sure, I can help with that! Here's a simple explanation:

Triglycerides are a type of fat found in your blood. When you eat, your body converts any calories it doesn’t need right away into triglycerides. If you regularly eat more calories than you burn, especially from high-carbohydrate foods, you may have high triglycerides (hypertriglyceridemia). Here's what you can do to lower them:

1. Eat Healthily: Aim to have a balanced diet full of fruits, vegetables, whole grains, lean proteins, and healthy fats. Avoid foods high in added sugars and unhealthy fats, such as processed foods, sweetened drinks, and fried foods.

2. Limit Bad Fats: Try to limit saturated fat found in meat and dairy, and avoid trans fat altogether, which is often in processed foods. Swap them for healthier fats found in foods like nuts, avocados, and fish.

3. Limit Alcohol: Even small amounts of alcohol can raise triglyceride levels. It's best to limit or avoid alcohol if you can.

4. Exercise Regularly: Try to get at least 30 minutes of exercise on most days of the week. It could be a brisk walk, bike ride, or anything that gets you moving.

5. Lose Weight: If you're overweight, losing even 5-10% of your total body weight can significantly lower your triglycerides.

6. Limit Simple Sugars: These are found in things like candy, soda, and desserts. Even fruit juice, honey, and maple syrup can raise your triglyceride levels.

Remember, it's always a good idea to talk to your doctor or a dietitian about what's best for you. They can give you personalized advice and check your progress. Sometimes, if lifestyle changes aren't enough, you might need medication to lower your triglycerides. You and your healthcare professional can decide on the best course of action.

***

Sure, I can definitely help with that. Triglycerides are a type of fat in your blood, and having high levels can increase the risk of heart disease. Here are some simple ways to decrease your triglyceride levels:

1. Change Your Diet: Foods high in sugars and unhealthy fats can raise your triglyceride levels. Try to eat more fruits, vegetables, whole grains, lean proteins (like chicken or fish), and healthy fats (like those found in avocados and olive oil). Cut down on foods like pastries, candies, sodas, and fried foods.

2. Exercise More: Regular physical activity can help lower your triglyceride levels. Try to do some sort of physical activity most days of the week, like walking, biking, or swimming. If you're new to exercising, it's good to start slow and gradually increase your activity level.

3. Limit Alcohol: Alcohol can significantly raise triglyceride levels. If you drink, try to do so in moderation.

4. Lose Weight: If you're overweight, losing even a small amount of weight can help lower your triglyceride levels. Even a 5-10% decrease in body weight can make a big difference.

5. Quit Smoking: If you smoke, quitting can help lower your triglyceride levels, among many other health benefits.

6. Medication: In some cases, you may need medication to help lower your triglyceride levels. This is usually considered if lifestyle changes aren't enough.

Remember, it's always a good idea to talk to a healthcare professional before making major changes to your lifestyle, especially if you have other health conditions. They can give you more personalized advice based on your specific situation.

***

Sure, here's a simple breakdown of how you can lower your triglycerides:

1. Healthy Eating: Choose healthier foods. Eat more fruits, veggies, lean meats (like chicken or fish) instead of red meat, and whole grains. Limit foods high in sugar, like sweets and sugary drinks, as well as saturated fats found in fatty meats and high-fat dairy products.

2. Exercise: Get moving! Regular physical activity can help lower your triglyceride levels. Aim for at least 30 minutes of exercise, like walking, cycling, or swimming, most days of the week.

3. Watch your Weight: If you're overweight, shedding even a few pounds can help lower your triglycerides. Even a modest weight loss can make a big difference.

4. Limit Alcohol: Alcohol can raise triglyceride levels. If you drink, do it in moderation or, better yet, not at all.

5. Don't Smoke: Smoking can increase your triglycerides and harm your heart. If you smoke, seek help to quit.

6. Control Your Conditions: If you have health problems like diabetes or high blood pressure, make sure you're keeping them under control. These conditions can affect your triglyceride levels.

Remember, these steps should be part of a plan you make with your healthcare professional. They know your personal health situation best, and can help guide you through these changes or prescribe medications if needed.

***

**What is lipoprotein(a)?**

**Answer this question using plain and easy-to-understand language in your response. Your response should mention transport of lipids or cholesterol, association with atherosclerotic cardiovascular disease, and heritability.**

***

Sure, let's break this down in simple terms.

Lipoprotein(a), often shortened to Lp(a), is a tiny particle in your blood that carries fats, or lipids, and cholesterol around your body. Think of it like a little delivery truck that moves these substances where they need to go.

Now, everyone needs some fat and cholesterol in their bodies for things like making hormones and protecting nerves. But, too much can be a problem, especially if it's the wrong type. This is where Lp(a) comes into play.

While Lp(a) is part of the normal set of particles in our blood, having high levels of it can increase the risk of atherosclerotic cardiovascular disease. This long phrase basically means disease in your heart and blood vessels that's caused by a buildup of fatty substances, cholesterol, cellular waste products, calcium, and fibrin (a clotting material in the blood) in the artery walls. Over time, these deposits harden and narrow the arteries, which is not a good thing because it can limit the flow of oxygen-rich blood to your organs and other parts of your body.

So, when there's too much Lp(a) in the blood, it can contribute to this buildup, leading to heart disease, stroke, and blockages in your blood vessels.

Lastly, let's talk about heritability. This means that the levels of Lp(a) in your blood are largely determined by the genes you inherit from your parents. It's not something that can be easily changed with diet or lifestyle, which is different from other types of cholesterol in your body.

I hope this helps make Lipoprotein(a) a bit clearer. It's one of those things that can play a big role in heart disease, but many people may not even know it exists!

***

Sure, I'd be happy to explain that.

Lipoprotein(a), or Lp(a) for short, is a kind of particle in your blood that carries fats, or lipids, and cholesterol around your body. Just like how a delivery truck carries packages to different places, Lp(a) takes these lipids and cholesterol to wherever they're needed.

But here's the catch. Sometimes, having too much Lp(a) in your blood isn't good for your health. It's kind of like how too many trucks on a road can lead to traffic jams. In the case of Lp(a), high levels can lead to a condition called atherosclerotic cardiovascular disease. This is a long name for a disease where your blood vessels get hard and narrow because of fat and cholesterol build-up. When this happens, it's harder for blood to flow around your body, which can lead to problems like heart attacks and strokes.

Now, you might be wondering, "Why do some people have more Lp(a) than others?" Well, that's mostly down to your genes, which you inherit from your parents. These genes decide a lot of things about you, including the level of Lp(a) in your blood. So if your parents had high levels of Lp(a), there's a chance you could too. This is what we mean when we say Lp(a) is heritable.

So in summary, Lp(a) is like a delivery truck for lipids and cholesterol in your blood. Too many of these trucks can lead to problems like heart disease, and the number of trucks you have is largely decided by your genes.

***

Lipoprotein(a) - let's call it Lp(a) for short - is like a little delivery truck in your body. Its job is to transport fats, including cholesterol, around in your bloodstream.

You might think of cholesterol as a necessary building block your body uses to make certain things it needs. Just like with a building project, if you have too many blocks in one place, it can cause problems. That's where Lp(a) comes in. It helps to keep these fats or 'building blocks' moving so they don't pile up and cause blockages.

But, sometimes, Lp(a) can actually contribute to the problem. If you have too much Lp(a), it can lead to a condition called atherosclerosis, which is a fancy way of saying 'hardening of the arteries'. This happens when fats and cholesterol build up in your artery walls, making them narrow and stiff. That makes it harder for blood to flow and can lead to heart disease.

An interesting thing about Lp(a) is that its levels in your body are largely controlled by your genes - the instructions passed down from your parents. That means if your mom or dad had high Lp(a) levels, there's a good chance you might, too. So, unlike other risk factors for heart disease, like diet or exercise, you can't really change your Lp(a) levels just by living a healthier lifestyle.

So, in short, Lp(a) is a little delivery truck in your body, moving fats and cholesterol around. But if you have too much of it, it might contribute to clogged arteries and heart disease. And, unfortunately, its levels in your body are largely decided by your genes.

***

**How can I quit smoking?**

**Answer this question using plain and easy-to-understand language in your response. Your response should suggest making a quitting plan and mention at least one specific medication that can help.**

***

Sure, I can help with that. Here are some steps you can take to quit smoking.

1. Decide a Quit Day: Set a date within the next two weeks. This gives you enough time to get ready, but not enough time to change your mind.

2. Tell People About It: Let your friends, family, and co-workers know you're planning to quit. They can offer support.

3. Understand Your Triggers: What situations make you want to smoke? Avoid these as much as possible, especially during the first few weeks.

4. Get Rid of Smokes: On your quit day, make sure you don't have any cigarettes, lighters, or ashtrays around.

5. Stay Active: Physical activity can help distract you from tobacco cravings and reduce their intensity.

6. Eat Healthy Snacks: When you get the urge to smoke, distract yourself by eating something healthy like a piece of fruit.

7. Reward Yourself: Quitting smoking is a big deal. Reward yourself for reaching milestones.

It's also worth noting that some people find medications can help them quit smoking. One commonly used medicine is nicotine replacement therapy (NRT). It comes in several forms like gum, patches, nasal sprays, inhalers, and lozenges. These products give you a small dose of nicotine which can help curb your cravings and ease withdrawal symptoms.

Before starting any medication, it's best to talk to your healthcare provider as they can provide guidance based on your health history and current situation.

Remember, it's okay if quitting takes a few tries. It's not easy, but with a solid plan and support, you can do it. Good luck!

***

Sure, quitting smoking can be a tough journey, but I'll break it down into steps to make it simpler.

1. Make a Plan: First, decide on a "Quit Day". This is the day when you'll stop smoking completely. It can be in a week or a month, but don't push it too far away. Pick a day that's not too stressful for you.

2. Identify Your Triggers: Figure out when you smoke or what makes you want to smoke. This could be when you're stressed, after a meal, or when you're with certain friends. Knowing your triggers can help you avoid them or prepare for them.

3. Find Healthy Alternatives: When you feel the urge to smoke, do something else instead. You could drink water, eat a healthy snack, exercise, or call a friend. The goal is to distract yourself until the craving passes.

4. Get Support: Let your friends and family know that you're quitting so they can help you. You can also join a support group or speak with a counselor. They can provide encouragement and tips to stay on track.

5. Consider Medication: There are medications that can help you quit. Nicotine replacement therapy, for example, is available in forms like patches, gum, and lozenges. They help to reduce withdrawal symptoms and cravings. One specific medication you could consider is "varenicline" (also known as Chantix). It's a prescription medication that can reduce cravings and block the pleasurable effects of smoking. Always speak with your doctor before starting any new medication.

6. Stay Active: Regular physical activity can help curb cravings and reduce withdrawal symptoms. Choose an activity you enjoy and aim for 30 minutes of moderate exercise most days of the week.

7. Reward Yourself: You're doing something great for your health, so reward yourself! Use the money you save from not buying cigarettes to treat yourself to something special.

Remember, if you slip up and smoke, don't beat yourself up. It happens. Just get back on track and keep going. And always consult with your doctor or healthcare professional for personalized advice. You can do it!

***

Quitting smoking can be a big challenge, but it is totally doable. Here are some steps to help you create a plan to quit smoking:

1. Decide a 'Quit Date': Pick a date within the next two weeks when you'll completely stop smoking. It gives you enough time to prepare. On that day, get rid of all cigarettes, lighters, and ashtrays.

2. List your reasons to quit: Write down all the reasons why you want to quit. It might be for your health, your family, saving money, or something else. Keep this list in a place where you can see it every day.

3. Identify your smoking triggers: These are situations, feelings, or people that make you want to smoke. Common triggers include stress, drinking alcohol, or after a meal. Once you identify them, think about ways to avoid or deal with these triggers without smoking.

4. Choose a support team: Quitting can be easier when you have people to support you. Tell your friends and family about your plan and ask for their help. You could also join a stop-smoking group or talk to a counselor.

5. Stay active: Physical activity can help you manage stress and reduce cravings. Choose activities that you enjoy. It can be as simple as walking, dancing, or cycling.

6. Use quit smoking aids: These are tools that can help you deal with the physical withdrawal symptoms of quitting smoking. One of these tools is a medication called "nicotine replacement therapy" (NRT). NRT comes in several forms like patches, gum, nasal spray, inhalers, and lozenges. This medication gives you a bit of nicotine, but without all the harmful chemicals found in cigarettes. NRT can help reduce your cravings and make it easier to quit. You can get some forms of NRT over the counter at a pharmacy, but it's best to talk to your doctor first.

7. Prepare for challenges: There might be moments when you feel a strong urge to smoke. Make a plan on how you will handle these moments. It can be as simple as taking deep breaths, calling a friend, or doing something to distract yourself.

8. Celebrate your victories: Each day, week, or month you go without smoking is a big deal. Celebrate these milestones and treat yourself with something you enjoy.

Remember, quitting smoking is a journey. If you slip and smoke a cigarette, don't be too hard on yourself. Think about what led to it, and how you can avoid that trigger in the future. The important thing is to keep trying. Every attempt brings you one step closer to quitting for good.

Good luck with your journey to become smoke-free! It's one of the best things you can do for your health.

***

**What are the side effects of statins?**

**Answer this question using plain and easy-to-understand language in your response. Your response should mention muscle symptoms and non-muscle symptoms and should also state that side effects tend to be individualized. Finally, the response should recommend talking with a healthcare professional.**

***

Statins are medicines that doctors often prescribe to lower cholesterol levels in your blood, which can help prevent heart diseases and stroke. However, like all medications, statins can have side effects.

One common side effect that some people might experience is muscle symptoms. This can include things like feeling muscle pain, tiredness, or weakness that doesn't go away. In very rare cases, statins can cause severe muscle damage.

Non-muscle symptoms can also occur. Some people might have stomach problems like constipation, nausea, or diarrhea. Others might feel dizzy, have trouble sleeping, or get a rash or flush. Some studies also suggest that statins might slightly increase the risk of type 2 diabetes.

It's really important to remember, though, that everyone is different. Some people might not have any side effects at all, while others might have side effects that are more noticeable.

If you're taking statins and you're worried about side effects, the best thing to do is talk to your doctor or another healthcare professional. They can help you weigh the benefits and risks of taking statins, and they can suggest alternatives if needed.

Remember, it's never a good idea to stop taking a prescribed medication without first discussing it with your doctor. It's always best to get advice from a healthcare professional if you have any concerns.

***

Sure, I'd be happy to explain that in simple terms!

Statins are a type of medication that doctors often prescribe to help lower bad cholesterol levels in your blood. They're really helpful for many people, but like all medicines, they can sometimes cause side effects. It's important to remember that not everyone will experience these and they can vary from person to person.

Muscle Symptoms:

The most common issue some people might feel is muscle-related. This might mean soreness, tiredness, or weakness in your muscles. In rare cases, statins can lead to a severe muscle condition called rhabdomyolysis, which is very serious and needs urgent medical attention.

Non-Muscle Symptoms:

Apart from the muscles, there could be other side effects. These might include upset stomach, constipation, or diarrhea. Some people might feel a bit drowsy or dizzy, or experience a rash or flushing. Statins might even impact your liver function, but this is rare.

Now, it's important to note that these side effects can be individualized. This means what one person experiences might be different from what another person does. Some folks might have one or two mild side effects, others may have none at all, while a few might have more serious ones. It really depends on the person.

If you're taking statins and notice any of these side effects or anything else that doesn't feel right, it's a good idea to have a chat with your healthcare professional. They can provide advice that is specific to you, and may be able to adjust your dose or try a different medication if needed. Remember, you should never stop taking a prescribed medication without first discussing it with your healthcare provider.

Health is a complex thing, and it's always best to talk over any concerns or questions you have with the people who know you and your body best – your healthcare team!

***

Statins are a type of medicine used to lower cholesterol levels in your body. Sometimes, people taking statins might experience side effects, but remember, not everyone will have them and they can be very different from person to person.

One common side effect is muscle problems. Some people might feel soreness, tiredness, or weakness in their muscles. In rare cases, people might get a condition called rhabdomyolysis where the muscle breaks down and releases a substance that can harm your kidneys. However, this is extremely rare.

There are also non-muscle side effects. Some people might feel upset stomach, gas, constipation, or diarrhea. Others might experience a rash or flushing. Some people have reported memory loss or confusion, but this seems to be pretty rare. A few studies have also suggested that statins might increase the risk of developing type 2 diabetes, but more research is needed to understand this fully.

Finally, and very importantly, if you're on statins and you're worried about side effects, it's a good idea to talk with your doctor or another healthcare professional. They can help figure out whether your symptoms are due to the statin or something else, and they can help you weigh the benefits of lowering your cholesterol against the possible risks of side effects. Don't stop taking your medication without talking to your doctor first.

***

**I have muscle pain with a statin. What should I do?**

**Answer this question using plain and easy-to-understand language in your response. Your response should discuss options for stopping use and switching to a lower dose or different agent under the supervision of a healthcare professional; it should also recommend talking with a healthcare professional to rule out dangerous side effects such as rhabdomyolysis.**

***

I understand that you're having muscle pain while taking a statin. Here's what you should know, in a straightforward way:

1. Talk to your doctor: If you're experiencing muscle pain, it's important to let your doctor know right away. They can check to see if it's a serious problem, like rhabdomyolysis, which is a rare but dangerous side effect of statins that can cause severe muscle damage.

2. Stopping or changing your medication: Your doctor might recommend stopping the statin for a short while to see if your muscle pain gets better. If it does, they might suggest that you restart the same statin but at a lower dose, or switch to a different statin.

3. Switching to a different type of medication: If lowering the dose or switching to a different statin doesn't help, your doctor might suggest trying a different type of cholesterol-lowering medicine.

Remember, it's very important that you don't stop taking your statin or change your dose without talking to your doctor. Even if you're experiencing side effects, your statin might still be helping to lower your risk of heart disease and stroke. Your doctor can help you weigh the benefits and risks to decide what's best for you.

***

Sorry to hear you're having muscle pain with a statin, that can be tough. So, statins are a type of medicine to help lower your cholesterol. But sometimes, these can cause side effects, like muscle pain.

If this is happening, it's important to talk with your doctor or a healthcare professional. They need to know what's going on so they can help you figure out the best course of action.

You should never just stop taking your medicine on your own, because this might cause other problems. Instead, your doctor might suggest a few things. They might recommend taking a lower dose of the same statin, which could help to lessen your muscle pain.

Or, they could suggest switching to a different statin. There are many different types, and you might find that you don't have the same side effects with a different one.

But it's also important to make sure that the muscle pain isn't because of something more serious. For instance, a condition called rhabdomyolysis can happen in rare cases when you're taking a statin. This is a serious condition where the muscle tissue breaks down and can lead to kidney damage.

So, to sum it up: don't stop taking your statin on your own. Instead, chat with your doctor about what's going on. They can help you figure out the best way forward, whether that's lowering your dose, switching to a different statin, or looking into other causes for your muscle pain.

***

Alright, it sounds like you're having muscle pain while you're taking a medicine called a statin. Statins are often used to lower cholesterol levels, but they can sometimes cause side effects like the one you're experiencing.

First things first, it's important that you talk to your doctor or a healthcare professional about this. They can help figure out if the muscle pain is indeed due to the statin or if it's being caused by something else. They can also check if you have a severe side effect called rhabdomyolysis, which is a rare condition where muscle fibers break down and can potentially harm your kidneys.

Now, if it turns out that the statin is causing your muscle pain, your doctor might suggest a few things:

1. Lowering your dose: If you're taking a high dose of the statin, your doctor might suggest lowering it to see if that helps with the muscle pain.

2. Switching medications: There are many types of statins, and they all work a little bit differently. If one is causing you problems, another might work better for you. Your doctor could suggest trying a different statin to see if that one doesn't cause muscle pain.

3. Stopping the medication: In some cases, your doctor might suggest you stop taking the statin completely. But, this is usually a last resort because statins can be very helpful in managing cholesterol levels and reducing the risk of heart disease.

Remember, only your doctor can advise you on the best course of action based on your personal medical history and current health. So, it's really important that you get in touch with them and discuss your symptoms.

***

**My cholesterol is still high and I’m already on a statin. What should I do?**

**Answer this question using plain and easy-to-understand language in your response. Your response should mention specific non-statin agents such as ezetimibe, PCSK9 inhibitors, and inclisiran, as well as lifestyle modifications; it should also recommend talking with a healthcare professional.**

***

Sure, I'd be happy to help with this! It's important to remember that I'm an AI, so while I can give you some general suggestions, you should definitely have a chat with your healthcare professional for advice tailored to your specific situation.

If your cholesterol is still high even though you're already taking a statin, there are several things you could consider:

1. Lifestyle Modifications: It's always a good idea to look at your diet and exercise habits. Eating a diet low in saturated and trans fats can help lower your cholesterol. Regular physical activity, like walking or cycling, can also help. If you smoke, quitting would be very beneficial. Lastly, maintaining a healthy weight can make a big difference.

2. Ezetimibe (Zetia): This is a medication that can help lower your cholesterol by reducing the amount it absorbs from your diet. It can be used on its own or alongside a statin if one isn't enough.

3. PCSK9 Inhibitors: These are newer drugs, like evolocumab (Repatha) and alirocumab (Praluent), that can help lower LDL ("bad") cholesterol by making your liver more effective at removing it from your blood. These are often used for people who have a genetic condition that causes high cholesterol, or for those who haven't had success with other treatments.

4. Inclisiran (Leqvio): This is another newer type of cholesterol-lowering drug. It works in a different way from statins or PCSK9 inhibitors by targeting a specific gene in the liver. It's often used when other treatments haven't worked or can't be tolerated.

Again, it's really important that you talk with your doctor or another healthcare professional about these options. They can help you understand the potential benefits and risks, and decide what's best for your specific situation. They may also suggest further tests to better understand why your cholesterol is still high. And remember, it's okay to ask questions and seek a second opinion if you're unsure about anything.

***

Sure, let's talk about this in a simple way. First off, remember that only a healthcare professional can give you specific advice for your situation. You should definitely have a chat with them about this.

Now, here's the basic idea. Cholesterol is a type of fat in your blood. Too much of it can cause problems like heart disease. You're already taking a type of medication called a statin, which helps lower cholesterol. But, your cholesterol is still high, so you're wondering what else you can do.

There are other medications that you can take along with statins to help reduce your cholesterol levels. Here are a few examples:

1. Ezetimibe: This medication blocks the absorption of cholesterol from your diet. It can lower the amount of cholesterol in your blood.

2. PCSK9 inhibitors: These are a group of medications that work by increasing the ability of your liver to remove cholesterol from your blood. They are often given by injection.

3. Inclisiran: This is a newer drug that can help lower cholesterol levels. It works in a different way from statins and can be used alongside them.

Keep in mind that these are not the only options, and what's best for you will depend on your specific situation.

Aside from medications, there are also lifestyle changes you can make:

1. Healthy Diet: Try to eat less saturated and trans fats. These are often found in foods like red meat and full-fat dairy products. You should also eat more fruits, vegetables, whole grains and lean proteins.

2. Exercise: Aim for at least 30 minutes of exercise most days of the week. Even a brisk walk can help.

3. Quit smoking: If you smoke, quitting can significantly reduce your cholesterol levels and improve your heart health.

4. Limit Alcohol: Try to limit the amount of alcohol you drink. Too much alcohol can raise your cholesterol levels and also harm your heart in other ways.

5. Maintain a healthy weight: Losing excess weight can help lower your cholesterol.

Remember, this is all general advice. For a plan tailored to you, talk to your doctor or another healthcare professional. They can give you advice based on your specific health situation.

***

Sure, let me explain it in a simple way.

You're already taking a statin, which is a medicine to help lower your cholesterol. But if it's still high, there are other steps you could consider:

1. Lifestyle Changes: Start with the basics. Eat healthy foods that are low in fat and cholesterol. Get more exercise, and if you smoke, try to quit. If you're overweight, losing some pounds can also help lower your cholesterol.

2. Medicines: There are other drugs, besides statins, that can help.

- Ezetimibe: This is a drug that lowers cholesterol. It blocks the absorption of cholesterol in your gut.
- PCSK9 Inhibitors: These are drugs like evolocumab and alirocumab. They work by helping your liver get rid of more LDL (or "bad") cholesterol.
- Inclisiran: This is a newer drug that can help lower cholesterol. It works by decreasing the production of LDL cholesterol in your liver.

Remember, you shouldn't start or stop taking any medicines without talking to your healthcare professional first. These are options that can help, but they need to be used under the right supervision. It's always a good idea to have a conversation with your healthcare provider to determine what will work best for you.

***

**What medications can reduce cholesterol other than statins?**

**Answer this question using plain and easy-to-understand language in your response. Your response should mention at least two different specific agents such as ezetimibe, PCSK9 inhibitors, bempedoic acid, and inclisiran; it should also recommend talking with a healthcare professional.**

***

Sure, I can help with that. There are several types of medications your doctor might prescribe if statins are not suitable for you. Here are a couple of examples:

1. Ezetimibe: This drug works by reducing the amount of cholesterol your body absorbs from the food you eat. So, it can help lower your cholesterol levels.

2. PCSK9 Inhibitors: These are a new type of drugs that can significantly lower your bad cholesterol levels. Some examples include evolocumab and alirocumab. They work by helping your liver break down more bad cholesterol.

3. Bempedoic Acid: This is a newer medication that works by blocking an enzyme your liver needs to produce cholesterol. This can help lower your cholesterol levels.

4. Inclisiran: It's a novel kind of medication that works differently from the others. It reduces the production of a protein in the liver that influences the amount of cholesterol in the body. By reducing this protein, inclisiran can help lower your bad cholesterol levels.

Remember, these are all prescription medications. It's important to discuss with your healthcare professional about the best option for you, considering your overall health, lifestyle, and other medications you might be taking. They can guide you on the right track to manage your cholesterol levels.

***

Sure, I can explain that for you. If statins aren't the right fit for you, there are other types of medication your doctor might suggest to help lower your cholesterol levels.

1. Ezetimibe: This medicine works by reducing the amount of cholesterol your body absorbs from the food you eat. It can be used on its own or with statins if you need a little extra help to get your cholesterol levels down.

2. PCSK9 inhibitors: These are a newer type of medication. They work by helping your liver remove more LDL (or "bad") cholesterol from your blood. Examples include drugs like evolocumab and alirocumab. They're usually given as injections every 2-4 weeks.

3. Bempedoic acid: This one blocks an enzyme in the liver that's needed to make cholesterol. It's an oral medication that can be used on its own or with other cholesterol-lowering medications.

4. Inclisiran: This is a unique type of medication that reduces the production of LDL cholesterol in the liver by silencing a specific gene. It is usually given as an injection two times a year.

Remember, each of these medications has its own benefits and potential side effects, and some may be more suitable for you depending on your specific health condition and needs. It's really important to talk with your healthcare provider about which treatment might be the best option for you. They'll be able to help you weigh the pros and cons of each one based on your unique health history and goals.

***

There are several types of medications that can help lower cholesterol levels, besides statins. Here are a few examples:

1. Ezetimibe (Zetia): This medicine works by blocking the substance your body needs to make cholesterol, which means your body makes less of it. It's not as strong as statins, but it's often used in combination with them if statins alone are not enough.

2. PCSK9 inhibitors (like Praluent and Repatha): These are newer medications that work by helping your liver break down more LDL ("bad") cholesterol. They're usually used for people who can't take statins or need extra help lowering their cholesterol. These are given by an injection.

3. Bempedoic Acid (Nexletol): This is another newer type of medication that blocks an enzyme your body needs to make cholesterol. This can be used on its own or with a statin.

4. Inclisiran (Leqvio): This medication is also one of the newest players. It works by blocking the production of PCSK9 protein, helping the liver to remove more LDL cholesterol from the bloodstream. It's also given by injection.

Remember, while these medicines can be effective, they are not suitable for everyone due to potential side effects or interactions with other medications. It's important to talk to your doctor or healthcare professional before starting any new medication. They can help determine which one is right for you based on your overall health, lifestyle, and specific medical history.

***

**What is ezetimibe?**

**Answer this question using plain and easy-to-understand language in your response. Your response should mention that ezetimibe lowers cholesterol and that it is prescribed with or after statins; it should also describe the mechanism and location of action briefly.**

***

Ezetimibe is a type of medicine used to help lower the amount of cholesterol in your body. If you think of cholesterol as a type of fat in your blood, this medicine is like a tiny vacuum cleaner that helps to scoop up some of that fat and remove it from your system.

This medicine is often used together with another type of cholesterol-lowering drug known as a statin, or it can be used on its own if statins aren't suitable for some reason. Statins work in your liver, reducing the amount of cholesterol that it produces. Ezetimibe works differently. It goes to work in your gut, where it stops your body from absorbing cholesterol that comes from the food you eat.

So, by using ezetimibe and statins together, you're tackling the cholesterol problem from two different angles: reducing production in your liver and absorption in your gut. This can be a really helpful way to get cholesterol levels under control.

***

Ezetimibe is a type of medicine that is used to help lower "bad" cholesterol levels in your body. Cholesterol is a type of fat that our bodies need, but too much of the wrong kind can build up in our blood vessels and cause problems like heart disease.

You can think of ezetimibe as a little security guard in your intestines, which is the part of your body that takes in nutrients from the food you eat. When you eat something that has cholesterol in it, the cholesterol tries to enter your body through the walls of your intestines. But ezetimibe, the little security guard, blocks some of the cholesterol from getting in.

Ezetimibe can work on its own, but often it's given along with another type of medicine called statins, or after a patient has already tried statins. Statins are another type of cholesterol-lowering medicine that works in the liver, another part of your body that's important for managing cholesterol. Sometimes, using both ezetimibe and statins together can help lower cholesterol levels even more effectively.

Remember, it's always important to talk to your doctor about the best ways to manage your cholesterol, as medicines are usually just one part of a plan that also includes diet and exercise.

***

Ezetimibe is a type of medicine that is often used to help lower the amount of cholesterol in your body. Cholesterol is a fatty substance that your body needs, but having too much of it in your blood can lead to health problems, like heart disease.

Most of the time, doctors prescribe ezetimibe to people who are already taking another type of cholesterol-lowering medicine called a statin. Sometimes, if a statin isn't working well enough on its own, or if someone can't take a statin due to side effects, a doctor might prescribe ezetimibe instead or as an addition.

Now, let's talk about how ezetimibe works. Your body gets cholesterol in two ways: by making it itself and by absorbing it from the food you eat. Statins mainly work by reducing the cholesterol your body makes, while ezetimibe works a little differently. It specifically works in your digestive tract, which is where your body absorbs cholesterol from your food. Ezetimibe helps by blocking a special 'gateway' that your body uses to take in cholesterol from your food. This means less cholesterol ends up in your bloodstream, which can help keep your cholesterol levels healthy.

*******

**What are Repatha and Praluent?**

**Answer this question using plain and easy-to-understand language in your response. Your response should mention that Repatha and Praluent lower cholesterol, are prescribed with or after statins, and are injectable; it should also describe the mechanism of action briefly.**

***

Repatha and Praluent are medicines that doctors prescribe to help lower cholesterol. Cholesterol is a type of fat in your blood, and having too much of it can cause health problems, like heart disease. These drugs are usually given when other medicines, like statins, don't work well enough or cause unwanted side effects. Statins are the most commonly used medicines for lowering cholesterol.

Repatha and Praluent are a bit different from pills because you don't swallow them. They are injectable, which means they are given by shots, typically once or twice a month.

So, how do these drugs work? They target a protein in your body called PCSK9. PCSK9 is like a troublemaker for your cholesterol because it lowers the number of receptors on your liver cells that grab LDL (the "bad" cholesterol) out of your blood to break it down. When PCSK9 is around, fewer LDL gets taken out of your blood and broken down, and that can lead to high cholesterol levels. Repatha and Praluent act like PCSK9's nemesis — they bind to it and stop it from causing trouble, leaving more receptors free to remove the LDL cholesterol from your bloodstream. So, in the end, they help to reduce the amount of bad cholesterol in your blood.

***

Repatha and Praluent are medicines that doctors use to help lower cholesterol levels in the blood. Cholesterol is a type of fat that our bodies need, but too much of it can stick to the walls of our blood vessels and cause problems, like heart disease.

Sometimes, doctors first give patients a group of drugs called statins to lower cholesterol. If those don't work well enough, or if the patient can't take them for some reason, then doctors might prescribe Repatha or Praluent.

Unlike some other medicines that come in pills, you take Repatha and Praluent by injecting them into your body with a needle, similar to how some people with diabetes take their insulin.

Now, about how they work: our bodies have a neat way of getting rid of extra cholesterol. We have these things called LDL receptors that can "catch" cholesterol in the blood and pull it inside our cells to be broken down. But there's a protein in our bodies called PCSK9 that can get in the way and stop these LDL receptors from doing their job. What Repatha and Praluent do is they stick to this PCSK9 protein and stop it from blocking the LDL receptors. With the PCSK9 out of the way, the LDL receptors can catch more cholesterol and help lower the amount of it in our blood. So, in simple words, these drugs help our bodies' natural system for lowering cholesterol work better.

***

Repatha and Praluent are medicines that doctors often prescribe to help lower cholesterol. They're usually used when statin medications, another common type of cholesterol-lowering drug, aren't enough or when someone can't take statins due to side effects. Both Repatha and Praluent are given by an injection, not in pill form.

So, how do these drugs work? They operate a bit differently than statins. Both Repatha and Praluent target something called PCSK9, a protein in your body. When PCSK9 is active, it stops your liver from getting rid of bad cholesterol, also known as LDL cholesterol, in your blood. But when Repatha or Praluent blocks PCSK9, your liver can do a better job of removing that bad cholesterol. So, these medicines help your body get rid of more bad cholesterol, and that helps to lower your overall cholesterol levels.

***

**What is inclisiran?**

**Answer this question using plain and easy-to-understand language in your response. Your response should mention that inclisiran lowers cholesterol, is prescribed with or after statins, and is injectable; it should also describe the mechanism of action briefly.**

***

Inclisiran is a type of medicine that doctors use to help lower the amount of "bad" cholesterol (also known as LDL cholesterol) in your body. It's often used alongside or after other cholesterol-lowering medicines called statins.

The special thing about inclisiran is that it's not a pill you take every day, but an injection you get from your doctor. This might be a good option for people who have trouble remembering to take daily medication, or for those who can't take pills for some reason.

Here's how it works: Our bodies make cholesterol, and a big part of this process involves a protein called PCSK9. PCSK9 increases the amount of bad cholesterol in our blood. Inclisiran works by blocking the PCSK9 protein, which leads to less bad cholesterol in your blood.

So, in simple words, inclisiran is like a traffic cop that tells PCSK9 to stop, and this helps lower the amount of bad cholesterol in your blood.

***

Inclisiran is a medicine used to help lower cholesterol, which is a type of fat in your blood. Too much cholesterol can lead to health problems, like heart disease. This medicine is often prescribed to people who are already taking other cholesterol-lowering medicines called statins, or for those who tried statins but they didn't work well enough or caused side effects.

Unlike some medicines that you might take every day as a pill, inclisiran is given by an injection, typically a couple of times a year.

Now, let's talk about how inclisiran works. Inside our bodies, there's a molecule called PCSK9 that plays a role in managing our cholesterol levels. When there's too much PCSK9, cholesterol can build up in the blood. Inclisiran works by telling the body to make less PCSK9. With less PCSK9 around, the body can remove more cholesterol from the blood, which helps to lower the overall cholesterol levels.

To sum up, inclisiran is an injectable medicine that works to lower cholesterol, often prescribed along with or after other cholesterol-lowering medicines called statins. It works by reducing the amount of a specific molecule in the body, which allows the body to remove more cholesterol from the blood.

***

Inclisiran is a medicine that doctors use to help lower the level of "bad" cholesterol, also known as LDL cholesterol, in the blood. It's often given to people who are already taking other cholesterol-lowering drugs called statins, or to those who have tried statins but need more help to get their cholesterol down. The medicine is given as an injection, not a pill, usually twice a year.

Now, how does inclisiran work? It's actually pretty neat. Our bodies have a system for making cholesterol, and a big part of that system is controlled by a molecule called PCSK9. Inclisiran works by blocking the production of PCSK9. When there's less PCSK9, our bodies can remove more cholesterol from the bloodstream. This helps lower our overall cholesterol levels, which can help protect against heart disease.

***

**What are the side effects of Repatha and Praluent?**

**Answer this question using plain and easy-to-understand language in your response. Your response should mention injection site reactions, nasopharyngitis and flu-like symptoms, and allergic reactions.**

***

Sure, I can explain that for you. Repatha and Praluent are medicines that help lower bad cholesterol in your blood. But like most medicines, they can sometimes cause side effects.

1. Injection site reactions: This means you might feel some discomfort where you get the injection. This could be redness, itchiness, pain, or swelling right around where the needle went in.

2. Nasopharyngitis: This is just a fancy term for a common cold. It means you could experience symptoms like a stuffy or runny nose, a sore throat, or sneezing more than usual.

3. Flu-like symptoms: You might feel like you're coming down with the flu. This could include things like feeling tired, having a fever, body aches, or even chills.

4. Allergic reactions: Sometimes your body might react to the medicine in a way it shouldn't. This can cause symptoms like rashes, itching, or swelling (especially of the face, tongue, or throat), severe dizziness, or trouble breathing.

Remember, not everyone will experience these side effects, and they might go away on their own as your body gets used to the medicine. But if you do notice any of these symptoms, or if they get worse, you should contact your doctor right away. They can help decide if these side effects are something to worry about and what you should do about them.

***

Sure, I'd be happy to explain.

Repatha and Praluent are drugs used to lower bad cholesterol levels. Like any medicine, they can cause side effects, and while not everyone gets them, it's still important to know what they might be.

1. Injection site reactions: This is when the area of skin where the medicine is injected gets red, sore, itchy, or swollen. Think of how your skin might react if you get a bug bite.

2. Nasopharyngitis: This is a fancy name for the common cold. So, you might get symptoms like a stuffy or runny nose, sore throat, or sneezing.

3. Flu-like symptoms: These can feel like when you catch the flu. You might get a fever, feel tired, have muscle aches, or a headache.

4. Allergic reactions: This can happen if your body doesn't like the medicine. Symptoms can include skin rashes, difficulty breathing, swelling of the face, lips, tongue, or throat, and sudden feeling of being sick. This is more serious and you should seek immediate medical help if you notice these.

Remember, it's always important to tell your doctor about any side effects you're having. They can help you figure out how to deal with them and whether you should continue taking the medicine.

***

Sure, I'd be happy to help.

Repatha and Praluent are drugs that are used to lower cholesterol levels. They are injected into the body, so sometimes they can cause a reaction right where you get the shot. This reaction can include things like redness, itching, swelling, or pain at the injection site.

These medicines might also give you a stuffy or runny nose, or a sore throat. This is known as nasopharyngitis.

In addition, you might get flu-like symptoms from these drugs. This can feel similar to having the flu, with symptoms like fever, chills, tiredness, and muscle aches.

Lastly, some people might have an allergic reaction to these drugs. This can cause symptoms like itching, a rash, swelling of your face or throat, and difficulty breathing. If you have any of these symptoms, you should seek medical help right away because it can be serious.

Remember, it's important to tell your doctor about any side effects you're experiencing. They can help figure out what's going on and how to best manage it.

***

**Should I take aspirin to prevent heart disease?**

**Answer this question using plain and easy-to-understand language in your response. Your response should specify that the correct answer to this question depends on the individual and should recommend talking with a healthcare professional.**

***

Sure, I'd be happy to help explain this in simple terms!

Aspirin is a common medicine that people sometimes take to help prevent heart disease. It can help stop blood clots, which can lead to heart attacks or strokes. But, it's not a one-size-fits-all kind of deal.

Some people might benefit from taking aspirin regularly, while for others, it might not be the best choice. It all depends on your own health and medical history. Some people might have a high risk of bleeding, which could make taking aspirin risky.

So, if you're thinking about taking aspirin to prevent heart disease, it's really important to talk to a doctor or a healthcare professional first. They can help you understand your individual risks and benefits and decide if it's the right choice for you.

***

Sure, I'd be happy to explain this for you!

Aspirin is a type of medicine that can help some people lower their risk of heart disease. It does this by thinning your blood and making it less likely to form clots, which can cause heart attacks.

However, it's not a one-size-fits-all solution. For some people, taking aspirin can have drawbacks. This includes a higher risk of bleeding, especially in the stomach. So, whether you should take aspirin to prevent heart disease really depends on you, your health, and your risk factors for heart disease.

Even though it's tempting to make a decision based on what you read or hear, this is definitely one of those things you should chat about with a doctor or another healthcare professional. They know your health situation best, and they can help you weigh the potential benefits and risks of taking aspirin.

So, if you're thinking about taking aspirin to prevent heart disease, schedule a time to talk to your healthcare provider about it. They can give you advice that's tailored to your unique health needs.

***

Sure, let's break this down simply:

Aspirin is a type of medicine that can sometimes be used to help prevent heart problems. This can be because aspirin can help make your blood less sticky, which makes it less likely to form clots. Clots in your blood can cause heart attacks, so that's why some people might use aspirin for heart disease prevention.

But here's the thing: aspirin isn't right for everyone. It can cause side effects like stomach upset and bleeding. These side effects might be more risky for some people than the potential benefits.

So, if you're thinking about taking aspirin for heart disease, it's super important to talk to a doctor or healthcare professional first. They'll be able to consider your personal health history and tell you if aspirin would be a good idea for you, or if it might do more harm than good. It's all about what's best for your specific situation.

***

**My cholesterol panel shows triglycerides 400 mg/dL. How should I interpret this?**

**Answer this question using plain and easy-to-understand language in your response. Your response should state that 400 mg/dL is considered high and that lifestyle modification and medication may be recommended depending on individual circumstances; it should also recommend talking with a healthcare professional.**

***

Sure, I can help with that.

Triglycerides are a type of fat found in your blood. Your body uses them for energy. But too much of them isn't good. When we talk about your triglycerides level, the number 400 mg/dL is considered pretty high.

This could mean that there is too much fat in your blood, which can sometimes lead to problems like heart disease. So, it's something you should take seriously. But don't worry, there are things you can do to help bring this number down.

A good first step is to look at your lifestyle. This includes things like what you eat, whether you smoke, how much you move or exercise, and even how much alcohol you drink. These are all things you can change to help lower your triglycerides.

In some cases, your doctor might also recommend medication to help. This would depend on your individual situation, like your overall health and other risk factors.

Remember, the best thing to do now is to talk with a healthcare professional, like your doctor or a nurse. They can give you the best advice for your situation. They can help you understand this better and guide you on the right path to improve your health.

***

Absolutely, let me break this down in simple terms for you.

Cholesterol panels are tests that doctors use to look at the types of fats in your blood. One of these fats is called "triglycerides". When your triglycerides level is at 400 mg/dL, it's quite high. This might be important because high triglycerides can sometimes increase the risk of heart disease.

To help get your triglycerides down, your doctor might suggest changes in your lifestyle. This could mean eating healthier, exercising more, quitting smoking if you smoke, or cutting back on alcohol. Sometimes, lifestyle changes aren't enough on their own, so your doctor might also recommend medication.

However, everyone's health is unique and your doctor is the best person to guide you. So, make sure to talk with them about these results. They can help you understand what this means for your health and what you can do about it.

***

Alright, here's what this means in simple terms:

Your triglyceride level, which is a type of fat in your blood, is 400 mg/dL. This number is considered high. High levels can increase your risk for heart disease, so it's something you'd want to pay attention to.

So what can you do about it? Depending on your personal health and other factors, your doctor might suggest making some changes to your daily habits - things like eating healthier, getting more exercise, losing weight if you need to, or cutting down on alcohol. They may also consider prescribing you some medication to help bring the levels down.

But everyone's health needs are different. So it's best to have a talk with a healthcare professional to figure out what the right plan for you might be. They'll help you understand the results better and guide you on what to do next.

***

**My LDL is 200 mg/dL. How should I interpret this?**

**Answer this question using plain and easy-to-understand language in your response. Your response should state that 200 mg/dL is considered high, that it may indicate a genetic condition called familial hypercholesterolemia or a secondary cause, and that lifestyle modification and medication are likely to be recommended; the response should also recommend talking with a healthcare professional.**

***

Sure, let's break it down. LDL stands for low-density lipoprotein, and it's often called "bad" cholesterol. This is because when there's too much LDL in your blood, it can build up on the walls of your arteries and form plaques, which can lead to heart disease.

Your LDL level of 200 mg/dL is considered high. This could indicate that you might have a genetic condition called familial hypercholesterolemia. This means that high cholesterol runs in your family. It could also be due to secondary causes like unhealthy diet, lack of exercise, or certain health conditions.

Typically, when your LDL levels are this high, your doctor might recommend some changes to your lifestyle. These could include eating a healthier diet with less saturated fats, increasing physical activity, losing weight if you're overweight, and quitting smoking if you smoke. In some cases, medication might also be needed to help bring down these levels.

It's really important to talk to a healthcare professional about your LDL level. They can give you the best advice for your specific situation and help you make a plan to lower your LDL. You should not try to manage this on your own or based on information you read online, as everyone's situation can be different. Please reach out to your doctor as soon as possible to discuss this.

***

Alright, let's break it down in simple terms.

LDL stands for low-density lipoprotein. It's often referred to as "bad cholesterol" because it can build up in your arteries and cause problems. Your LDL level is 200 mg/dL, which is considered high.

This high number could mean a few things. It may suggest you have a genetic condition called familial hypercholesterolemia, which makes your body unable to remove LDL cholesterol properly. It might also be a sign of some other secondary cause, like a diet high in certain fats or a lack of physical activity.

Usually, if your LDL is this high, it's likely your doctor or healthcare professional will recommend changes in your lifestyle. This could be stuff like eating healthier, exercising more, quitting smoking, or reducing alcohol intake. They might also suggest medication to help lower your LDL levels.

Remember, though, it's really important to talk with a healthcare professional to understand what these results mean for you and to work out the best plan to manage it. I'm an AI trained by OpenAI and while I can provide some general information, I can't give personalized health advice. Hope that helps!

***

Okay, let's break this down simply. LDL, or "low-density lipoprotein," is often called "bad cholesterol" because high levels can lead to health issues, like heart disease.

Your LDL level is 200 mg/dL, which is quite high. Normally, an LDL level below 100 mg/dL is considered optimal for healthy individuals. This high LDL level might be due to a genetic condition called familial hypercholesterolemia or possibly a secondary cause like diet, lack of exercise, or certain health conditions.

This doesn't mean you should panic, but it's important to talk with a healthcare professional about this. They might recommend some lifestyle changes like eating healthier foods, getting more physical activity, or perhaps even taking medication to help lower your LDL levels.

Always remember, even though 200 mg/dL is high, it's not a life sentence - there are many ways to help reduce your LDL and improve your heart health. But it's definitely worth chatting with a doctor or other healthcare provider to figure out the best steps for you.

***

**What does a coronary calcium score of 0 mean?**

**Answer this question using plain and easy-to-understand language in your response. Your response should state that it means no calcium is seen in the region of the coronary arteries on a specific CT scan of the heart but that it does not imply zero plaque or zero risk of heart disease; the response should also recommend talking with a healthcare professional about preventative measures such as lifestyle modifications.**

***

Sure, I can explain that. A coronary calcium score of 0 means that no calcium was spotted in your heart's arteries when you had a special kind of CT scan. Calcium in these arteries can be a sign of heart disease because it's often found in fatty deposits or plaques that can build up there.

However, even though a score of 0 is a good thing because it means no calcium was seen, it doesn't mean there's absolutely no chance of having plaques or heart disease. Some plaques don't have calcium in them, so they might not show up on this type of scan.

It's always a good idea to talk with your doctor or other healthcare professional about how to keep your heart healthy. They can give you advice tailored to you about things like eating a balanced diet, getting regular exercise, and other ways to lower your risk of heart disease. Even if your calcium score is 0, there may still be ways you can improve your heart health.

***

Sure, I can explain that in simple terms.

A coronary calcium score of 0 means that no calcium was found in the arteries around your heart when you had a special kind of X-ray called a CT scan. This is generally a good sign because calcium in these arteries can suggest that there's some hardening or blockage going on, which can lead to heart problems.

However, having a score of 0 doesn't mean that there's absolutely no chance you have or could get heart disease. This is because not all plaques in the arteries have calcium in them. Some plaques are soft and don't have calcium, so they won't show up on this type of scan.

So, even with a score of 0, it's still important to live a healthy lifestyle to keep your heart in good shape. This includes eating a balanced diet, exercising regularly, not smoking, and managing stress. And it's always a good idea to chat with your doctor or another health professional about your personal risks and what you can do to prevent heart disease.

***

Sure, I'd be happy to explain this in a simple way.

A coronary calcium score of 0 means that the CT scan, a type of special X-ray test, did not pick up any calcium deposits in the blood vessels of your heart, which are called coronary arteries. Calcium can sometimes build up there as part of a process called atherosclerosis, where plaque forms on the inner walls of the arteries.

However, even if your score is 0, it doesn't mean there is absolutely no plaque at all. Sometimes, plaque doesn't contain calcium and therefore isn't picked up by this particular test. And it definitely doesn't mean there is zero chance of getting heart disease. There are many other factors like high blood pressure, smoking, diabetes, and high cholesterol that can increase your risk.

So even with a score of 0, it's still important to look after your heart health. Healthy lifestyle choices like eating a balanced diet, regular exercise, maintaining a healthy weight, not smoking, and managing stress can help a lot.

Remember, this score is just one piece of the puzzle. It's always a good idea to have a chat with your doctor or another healthcare professional about what you can do to prevent heart disease. They can help guide you on what measures are right for you.

***

**What does a coronary calcium score of 100 mean?**

**Answer this question using plain and easy-to-understand language in your response. Your response should state that it means calcium is seen in the region of the coronary arteries, that it may indicate plaque in the coronary arteries, and that lifestyle modifications and medication may be recommended; the response should also recommend talking with a healthcare professional.**

***

Sure, let's break this down into simple terms. A coronary calcium score of 100 is like a warning sign. It means that some calcium is spotted in the areas around the heart's arteries, which are the pipes carrying blood to your heart.

This could be a sign of plaque, a sort of gunk, building up in those arteries. This plaque is not a good thing because it can make it harder for blood to flow, which could potentially lead to heart problems.

However, don't panic! This score doesn't mean you're definitely going to have a heart problem. It's just an indication that you might need to make some changes to help your heart out. This could mean exercising more, eating healthier, quitting smoking, or other lifestyle modifications. Sometimes, a doctor might also suggest certain medications to help keep your heart healthy.

Remember, it's super important to chat with your doctor or another healthcare professional about this. They're the best ones to guide you through what this score means specifically for you and what you should do next. They can give you more detailed advice based on your overall health and personal circumstances.

***

A coronary calcium score of 100 is a measurement taken from a special kind of heart scan. This score tells us that there's some calcium seen in the areas around your heart arteries, which are important blood vessels that supply your heart muscle with blood. Calcium is not supposed to be there normally, and its presence can be a clue that there's plaque building up in these arteries, like gunk in a pipe.

Plaque is a mix of fat, cholesterol, calcium, and other substances, and its build-up can narrow or block these heart arteries over time. This is often what leads to heart disease and can increase the risk of heart attacks.

Now, a score of 100 is considered moderate, meaning you have a moderate amount of calcium in your arteries. It doesn't mean you'll definitely have a heart problem, but it does mean you're at higher risk compared to someone with a lower score.

Because of this, doctors may suggest some changes in your lifestyle - like healthier eating, more exercise, quitting smoking, or reducing stress. They might also recommend certain medications to manage cholesterol levels or blood pressure to help reduce the risk of further plaque build-up.

However, it's very important to talk with your healthcare professional about what this score means for you. Everyone is different and the best plan will depend on other factors like your age, overall health, family history, and whether or not you have other risk factors for heart disease.

***

A coronary calcium score of 100 is like a warning sign. It tells us that there's some calcium in the areas around the heart's blood vessels, which we call the coronary arteries. Now, you might be wondering why we care about calcium in the heart area. Well, the calcium itself isn't the problem - it's more like a clue. When we see calcium, it often means there's plaque build-up in these arteries.

Imagine a river flowing freely. But then, over time, rocks and debris start to pile up and slow down the water. Similarly, in your body, the plaque is like those rocks and debris, and it can slow down or block the blood flow in your heart. This could lead to heart disease.

Because of this, if your score is 100, your doctor might suggest you make some changes to your lifestyle. This could include eating healthier, doing more exercise, or quitting smoking if you smoke. In some cases, you might also need to take certain medications.

It's important to remember that this is just a general explanation. The best thing to do is have a chat with your healthcare professional. They can give you advice that's tailored to you and help you understand what this score means for your health.

***

**What does a coronary calcium score of 400 mean?**

**Answer this question using plain and easy-to-understand language in your response. Your response should state that it means calcium is seen in the region of the coronary arteries, that it likely indicates plaque in the coronary arteries, and that lifestyle modifications and medication may be recommended; the response should also recommend talking with a healthcare professional.**

***

A coronary calcium score of 400 is like a warning sign. This score comes from a test that looks for calcium in your coronary arteries, the blood vessels that supply your heart. The presence of calcium in these arteries is not a good thing because it typically means there's plaque buildup there.

Plaque is a mix of fat, cholesterol, calcium, and other substances found in your blood. When it builds up in your arteries, it can harden and narrow them, a condition we often call "hardening of the arteries" or more formally, atherosclerosis. This can make it harder for blood to flow to your heart, and that's not good. It can sometimes lead to heart disease or even a heart attack.

A score of 400 suggests that there's a significant amount of plaque. This might mean you have a higher risk of having heart problems in the future, compared to someone with a lower score.

After getting a score like this, it's really important to chat with your doctor or another healthcare professional. They can help you understand what this means for you specifically. They might also recommend lifestyle changes like eating healthier, exercising more, quitting smoking if you smoke, or cutting down on alcohol. Additionally, they might prescribe some medication to help reduce your risk of heart problems.

Remember, this score is not a diagnosis, but it's a sign that you need to take care of your heart health. It's crucial to discuss these results and the next steps with your healthcare provider.

***

Sure, I'd be happy to help explain what a coronary calcium score of 400 means in an easy-to-understand way.

Firstly, the coronary calcium score is a number that doctors use to check for signs of heart disease. This score comes from a special heart scan that looks for calcium, a sign of plaque, in the coronary arteries. These arteries are the ones that supply blood to your heart.

A score of 400 is quite high. This means that there's a good amount of calcium seen in your coronary arteries. In plain terms, you can think of calcium in this case like a signpost for plaque - if there's a lot of calcium, there's likely a lot of plaque too. Plaque is a build-up of fats and other substances, which can narrow or even block the arteries. This can increase the risk of heart disease, including heart attacks.

If you have a coronary calcium score of 400, your doctor may recommend some changes to your lifestyle, like eating a healthier diet, getting more exercise, or quitting smoking if you're a smoker. They might also recommend taking certain medications to help lower your risk of heart disease.

Remember, it's important to discuss your coronary calcium score and what it means for your health with a healthcare professional. They can give you personalized advice based on your overall health, your medical history, and other factors.

***

Sure, I can explain that.

A coronary calcium score of 400 means there's quite a bit of calcium in the area around your heart's arteries. This is important because it usually means there's plaque there too. Plaque is a sort of gunk made from fat, cholesterol, calcium, and other substances in the blood that can build up in your arteries over time. When it gets too thick, it can block blood flow, and that can be dangerous for your heart.

Having a score of 400 doesn't mean you're about to have a heart attack right now, but it does mean you're at a higher risk for heart problems in the future. This is something you should take seriously, but don't panic. It's a wake-up call to take care of your heart health.

Usually, doctors would suggest making changes to your lifestyle. This could mean eating healthier, exercising more, quitting smoking if you do, and cutting down on alcohol. They might also suggest medications to help control things like high cholesterol or high blood pressure.

But remember, this is a general explanation. Your situation could be different, so it's really important to talk with a healthcare professional. They can look at your specific case, explain what your score means for you, and help you figure out the best plan.

***

**What genetic mutations can cause high cholesterol?**

**Answer this question using plain and easy-to-understand language in your response. Your response should mention mutations in the LDL receptor, APOB, and PCSK9, as well as other causes such as specific mutations in LDLRAP1. Alternatively, your response can discuss familial hypercholesterolemia.**

***

Sure, I'd be happy to explain that in simple terms.

Your body has cholesterol, a type of fat that's actually pretty important for your body. But, like most things, too much can cause problems. Some people have higher cholesterol because of their genes – that means it's in their DNA, which is passed down from their parents.

Here are some examples:

1. LDL Receptor mutations: Think of your body's cells as rooms and cholesterol as visitors. The LDL receptor is like the room's door – it lets cholesterol in. But if there's a mutation, or change, in the gene that makes this door, it might not work well. That means cholesterol can't get in and ends up building up in your blood.
2. APOB mutations: Now, the APOB gene is like the ID card for cholesterol. When it works properly, it helps the LDL receptor recognize and let cholesterol in. If there's a change in the APOB gene, the ID card might not work. So again, cholesterol can't get into the cells and builds up in the blood.
3. PCSK9 mutations: The PCSK9 gene helps control the number of doors (LDL receptors). If there's a mutation here, it might make too many doors disappear. So, even if there's plenty of cholesterol ready to get in, they can't because there aren't enough doors.
4. LDLRAP1 mutations: This one's a bit more complex. LDLRAP1 is like the door's manager. If there's a change in this gene, the manager may not do their job properly, and the doors (LDL receptors) won't work as they should. So, yet again, cholesterol builds up in your blood.

Familial hypercholesterolemia (FH) is a specific condition where mutations in LDL receptor, APOB, or PCSK9 genes often occur. People with FH have very high cholesterol levels from birth, which can cause health issues like heart disease at a young age.

Remember, genes aren't the whole story – diet, exercise, and other factors can also affect your cholesterol. But understanding these gene changes can help scientists find treatments for high cholesterol.

I hope that's helpful! Let me know if you have any other questions.

***

High cholesterol, especially when it's present from a young age, can sometimes be caused by specific changes in certain genes. These genes play a crucial role in how your body processes cholesterol. Here are the four key genes you mentioned:

1. LDL receptor (LDLR): LDLR is like a doorway on the surface of your body's cells that lets "bad" cholesterol, known as LDL cholesterol, inside the cells to be used. If there's a mutation or a problem with this gene, these doorways don't work properly. That means LDL cholesterol can't get inside the cells and instead builds up in your bloodstream.
2. APOB: The APOB gene helps create a part of the LDL cholesterol particle that recognizes and docks with the LDL receptor. It's like a key that opens the LDLR door. If you have a mutation in this gene, the key might not fit the lock properly, making it harder for LDL cholesterol to enter cells. This causes more LDL cholesterol to stay in your blood.
3. PCSK9: The PCSK9 gene helps control the number of LDL receptors your body makes. If this gene has a mutation, your body might end up making too few LDL receptors. With fewer doors available for LDL cholesterol to get into cells, more of this cholesterol stays in your blood.
4. LDLRAP1: This gene is like the helper that assists LDL receptors in doing their job. If there's a mutation here, the LDLR may not work well, leading to more LDL cholesterol remaining in your blood.

Familial hypercholesterolemia (FH) is a condition often caused by mutations in one of these four genes. It's a genetic disorder that makes your body unable to remove LDL cholesterol from your blood efficiently, leading to very high cholesterol levels. People with FH have a higher risk of developing heart disease at a young age.

***

Sure, I can help with that. Let's imagine your body as a city and cholesterol as a group of tourists trying to get around. To get to where they need to go, they need taxis (these are like your LDL receptors).

A genetic mutation in the LDL receptor is like if all the taxis in your city stopped working properly. Normally, the LDL receptors (taxis) are supposed to pick up cholesterol (tourists) from the blood and carry it into cells where it's needed. But, if there's a mutation in the LDL receptor, it's like the taxis aren't doing their job properly. This means more cholesterol (tourists) is left wandering around in the blood, which can increase the risk of heart disease.

A mutation in the APOB gene is like if the tourists can't find the taxis. The APOB gene provides instructions for making a protein that attaches to cholesterol, helping it connect with the LDL receptor (taxi). If there's a mutation in this gene, the connection doesn't happen as efficiently, and again, more cholesterol is left in the blood.

A mutation in the PCSK9 gene is like if there are too many taxis being sent to the junkyard prematurely. PCSK9 is involved in controlling the number of LDL receptors (taxis) available. If it's overactive due to a mutation, too many LDL receptors are removed from the surface of cells, meaning not enough are left to remove cholesterol from the blood.

Finally, LDLRAP1 is another gene that can cause problems if it's mutated. This gene plays a support role in helping LDL receptors (taxis) pick up cholesterol. It's like the taxi dispatcher. If there's a mutation in this gene, the dispatcher doesn't work properly, and again, cholesterol can build up in the blood.

All of these situations can cause something called familial hypercholesterolemia. This is a fancy term for "your family has a history of high cholesterol". It means that if your parents, grandparents, or other close relatives have had high cholesterol, you might be at risk too. It's like if a taxi company was run by a family, and the whole family didn't do a great job of running the taxis.

Remember, these are just some of the genetic reasons why someone might have high cholesterol. Diet, exercise, and other lifestyle factors can also play a big role.
