# Supplementary material for: Evaluation of Prompts to Simplify Cardiovascular Disease Information Generated Using a Large Language Model: Cross-Sectional Study
Source: J Med Internet Res. 2024 Apr 22;26:e55388. doi: 10.2196/55388 (PMC11074888; doi:10.2196/55388)
Supplement: Multimedia Appendix 3 [file jmir_v26i1e55388_app3.docx]

**Multimedia Appendix 3: Custom Scoring Rubric**

1. **How can I prevent heart disease?**
2. Diet
3. Exercise
4. Risk factor control (mention at least one example such as cholesterol, smoking, weight, sleep, glucose/diabetes, blood pressure)
5. Discussion with a healthcare professional

**Complete score: 4/4**

1. **What is the best diet for the heart?**
2. Promote vegetable/fruit/plant-based intake
3. Avoid “refined” carbohydrates and/or processed foods
4. Avoid “unhealthy” fats and/or red meat, or promote “healthy” fats
5. Avoid excess sodium

OR

Mention of an ACC/AHA-recommended diet such as the Mediterranean diet or DASH diet

**Complete score: 4/4 or 1/1 (complete)**

1. **What is the best diet for high blood pressure and high cholesterol?**
2. Promote vegetable/fruit/plant-based intake
3. Avoid “refined” carbohydrates and/or processed foods
4. Avoid “unhealthy” fats and/or red meat, or promote “healthy” fats
5. Avoid excess sodium

OR

Mention of an ACC/AHA-recommended diet such as the Mediterranean diet or DASH diet

**Complete score: 4/4 or 1/1**

1. **How much should I exercise to stay healthy?**
2. 150 minutes of moderate intensity or 75 minutes of vigorous intensity exercise in accordance with ACC/AHA guidelines
3. Recommendation to discuss with a professional

**Complete score: 2/2**

1. **Should I do cardio or lift weights to prevent heart disease?**

The correct response to this question depends on the individual. The model should recommend visiting with a healthcare professional or exercise physiologist to discuss these considerations.

**Complete score: 1/1**

1. **How can I lose weight?**
2. Lifestyle modification (diet)
3. Lifestyle modification (exercise)
4. Discussion with a healthcare professional (to ensure safe, effective approaches, which can include mention of medications in this context)

**Complete score: 3/3**

1. **How can I decrease LDL?**
2. Lifestyle modification (diet and exercise)
3. Medication with at least one specific example given (e.g., statins)
4. Discussion with a healthcare professional

**Complete score: 3/3**

1. **How can I decrease triglycerides?**
2. Lifestyle modification (diet and exercise)
3. Limit alcohol use
4. Weight and glucose control
5. Discussion with a healthcare professional

**Complete score: 4/4**

1. **What is lipoprotein (a)?**
2. Mention of “lipid” or “cholesterol”
3. Mention of association with atherosclerotic cardiovascular disease (including aortic stenosis)
4. Mention of heritability

**Complete score: 3/3**

1. **How can I quit smoking?**
2. Make a quitting plan
3. Medication support and options, including at least 1 example

**Complete score: 2/2**

1. **What are the side effects of statins?**
2. Mention of muscle symptoms
3. Mention of non-muscle symptoms
4. Mention that symptoms are individualized and/or uncommon
5. Recommend discussion with healthcare professionals if experiencing symptoms

**Complete score: 4/4**

1. **I have muscle pain with a statin. What should I do?**
2. Discuss options for challenging, including stopping and resuming a lower dose or different agent
3. Discuss with healthcare professional to rule out a dangerous side effect such as rhabdomyolysis

**Complete score: 2/2**

1. **My cholesterol is still high and I’m already on a statin. What should I do?**
2. Discuss options for non-statin agents, with specific mention of at least one example (e.g., ezetimibe, PCSK9 inhibitors, inclisiran, bile acid sequestrants)
3. Lifestyle modification (diet and exercise)
4. Discussion with healthcare professional

**Complete score: 3/3**

1. **What medications can reduce cholesterol other than statins?**
2. Ezetimibe
3. PCSK9 inhibitors
4. Other agents

**Complete score: 3/3**

1. **What is ezetimibe?**
2. Lowers “cholesterol” or “LDL”
3. Prescribed with statins or after statins

**Complete score: 2/2**

1. **What are Repatha and Praluent?**
2. Lower “cholesterol” or “LDL”
3. Prescribed with statins or after statins
4. Injectables
5. Act on PCSK9

**Complete score: 4/4**

1. **What is inclisiran?**
2. Lowers “cholesterol” or “LDL”
3. Prescribed with statins or after statins
4. Injectable
5. A comment on mechanism (e.g., mention of siRNA or PCSK9)

**Complete score: 4/4**

1. **What are the side effects of Repatha and Praluent?**
2. Injection site reactions
3. Nasopharyngitis and/or flu-like reactions
4. Allergic reactions

**Complete score: 3/3**

1. **Should I take aspirin to prevent heart disease?**

The correct response to this question depends on the individual. The model should recommend discussion with a healthcare professional before making this decision.

**Complete score 1/1**

1. **My cholesterol panel shows triglycerides 400 mg/dL. How should I interpret this?**
2. Considered high
3. Lifestyle modification and/or medications may be recommended (depending on individual situation)
4. Discussion with a healthcare professional

**Complete score 3/3**

1. **My LDL is 200 mg/dL. How should I interpret this?**
2. Considered high
3. Could indicate a genetic condition called familial hypercholesterolemia or a “secondary” cause
4. Lifestyle modification
5. Medications are likely to be recommended
6. Discussion with a healthcare professional

**Complete score 5/5**

1. **What does a coronary calcium score of 0 mean?**
2. No calcium is seen in the region of the coronary arteries on a specific CT scan of the heart
3. Does not mean that there is zero risk or zero plaque
4. Discussion of preventive measures (including lifestyle) with a healthcare professional

**Complete score 3/3**

1. **What does a coronary calcium score of 100 mean?**
2. There is calcium seen in the region of the coronary arteries
3. Could indicate plaque in the coronary arteries
4. If qualifier is given, “moderate” to “severe” should be used
5. Lifestyle modification
6. Medications may be recommended (such as statins or aspirin)
7. Discuss with a healthcare professional

**Complete score 6/6**

1. **What does a coronary calcium score of 400 mean?**
   1. There is calcium seen in the region of the coronary arteries
   2. Likely indicates at least some plaque in the coronary arteries
   3. If qualifier is used, “severe” should be used
   4. Lifestyle modification
   5. Medications may be recommended (such as statins or aspirin)
   6. Discussion with a healthcare professional

**Complete score 6/6**

1. **What genetic mutations can cause high cholesterol?**
2. LDL receptor
3. *APOB*
4. *PCSK9*
5. Others (*LDLRAP1*, etc)

**Complete score: 4/4**
